# Unified short syntheses of oxygenated tricyclic aromatic diterpenes by radical cyclization with a photoredox catalyst

**DOI:** 10.1038/s42004-023-00979-2

**Published:** 2023-08-21

**Authors:** Riichi Hashimoto, Kengo Hanaya, Takeshi Sugai, Shuhei Higashibayashi

**Affiliations:** Faculty of Pharmacy, Keio University, 1-5-30 Shibakoen, Minato-ku, Tokyo, 105-8512 Japan

**Keywords:** Natural product synthesis, Synthetic chemistry methodology, Photocatalysis

## Abstract

The biomimetic two-phase strategy employing polyene cyclization and subsequent oxidation/substitution is an effective approach for divergent syntheses of [6-6-6]-tricyclic diterpenes. However, this strategy requires lengthy sequences for syntheses of oxygenated tricyclic aromatic abietane/podocarpane diterpenes owing to the many linear oxidation/substitution steps after cyclization. Here, we present a new synthetic route based on a convergent reverse two-phase strategy employing a reverse radical cyclization approach, which enabled the unified short syntheses of four aromatic abietane/podocarpane diterpenes and the divergent short syntheses of other related diterpenes. Oxygenated and substituted precursors for cyclization were convergently prepared through Friedel-Crafts acylation and rhodium-catalyzed site-selective iodination. Radical redox cyclization using an iridium photoredox catalyst involving neophyl rearrangement furnished the thermodynamically favored 6-membered ring preferentially. (±)-5,6-Dehydrosugiol, salvinolone, crossogumerin A, and Δ^5^-nimbidiol were synthesized in only 8 steps. An oxygenated cyclized intermediate was also useful for divergent derivatization to sugiol, ferruginol, saprorthoquinone, cryptomeriololide, and salvinolone.

## Abstract (web summary)

Synthesis of the bioactive [6-6-6]-tricyclic diterpene skeleton has been developed using a biomimetic cyclization-oxidation strategy, however, the long oxidation steps hinder the total efficiency of the synthesis. Here, the authors develop a radical cyclization initiated from the C-ring using an oxidation-cyclization strategy in order to shorten the synthesis.

## Introduction

**Fig. 1:**
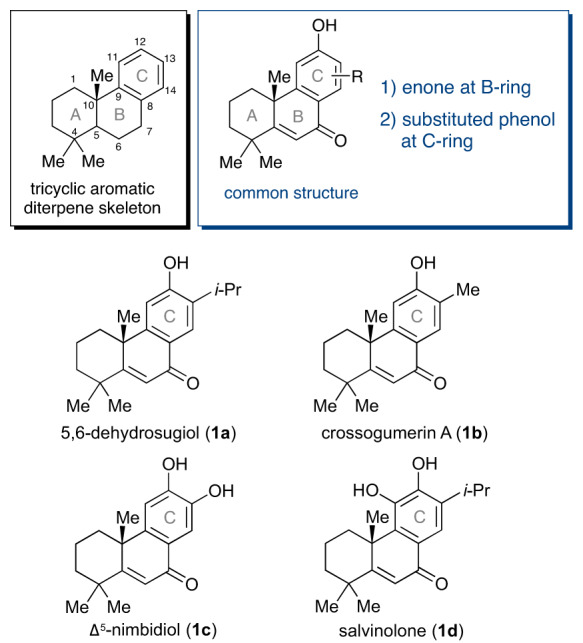
Naturally occurring tricyclic diterpenes possessing enone group in B-ring and substituted phenols in C-ring. Tricyclic aromatic diterpene skeleton and a common structure (blue) of 5,6-dehydrosugiol (**1a**), crossogumerin A (**1b**), ∆^5^-nimbidiol (**1c**) and salvinolone (**1d**).

Many diterpenes having a tricyclic [6-6-6]-fused skeleton with an aromatic C-ring (Fig. 1) such as aromatic abietanes have been isolated from natural resources^1,2^. Due to their unique carbon skeletons and attractive biological activities^2–5^, chemists have devoted themselves to developing syntheses of these tricyclic diterpenes^6,7^. In these synthetic approaches, a biomimetic strategy employing polyene cyclization followed by the introduction of functional groups by oxidation and substitution has been successful to produce many cyclic aromatic diterpenes^8–15^. This type of approach can be classified as a “two-phase strategy” consisting of the first, a cyclase phase, and the second, an oxidase phase, as recently described by Baran for terpenoid syntheses^16–18^.

**Fig. 2:**
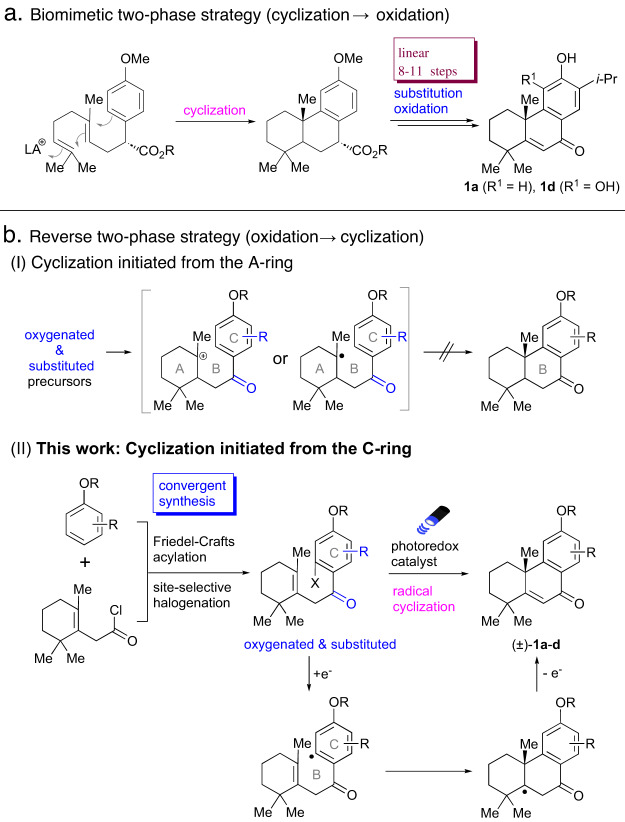
Two-phase strategy and reverse two-phase strategy for syntheses of tricyclic diterpenes. **a** Syntheses of 5,6-dehydrosugiol (**1a**) and salvinolone (**1d**) using two-phase strategy by Tada et al. **b** (I) Problem in the cationic or radical cyclization initiated from the A-ring in reverse two-phase strategy; (II) This work: Cyclization initiated from the C-ring in convergent reverse two-phase strategy.

Among the family of tricyclic aromatic diterpenes, a certain number of diterpenoids including 5,6-dehydrosugiol (**1a**)^19–21^, crossogumerin A (**1b**)^22^, ∆^5^-nimbidiol (**1c**)^23^, and salvinolone (**1d**)^24–26^ possess a common oxygenated structure: 1) an enone group in the B-ring and 2) a substituted phenol in the C-ring (Fig. 1). These diterpenes exhibit a variety of bioactivities such as antitumor^27–29^, antibacterial^30^, antitermitic^31^, and antioxidant activities^32^. One total synthesis of **1a** and **1d** has been reported as a result of the above-mentioned two-phase strategy^30^. Two total syntheses also constructed the B-ring by cyclization between A- and C-rings followed by the formation of the enone goup^33,34^. Tada et al. synthesized abietane diterpenoids including **1a** and **1d** by cationic polyene cyclization forming a common tricyclic intermediate and its divergent functionalization (Fig. 2a), which was successful in producing various derivatives for evaluation of the antibacterial activities^30,35^. However, focusing on the total efficiency of the syntheses of **1a** and **1d**, the functionalization after cyclization required 8-11 linear steps, for a total of 11-14 steps, respectively. Salvinolone (**1d**) has also been prepared semisynthetically from dehydroabietic acid^36^.

A strong candidate for the realization of shorter syntheses of the family of **1a**–**d** would be the convergent preparation of oxygenated and substituted precursors possessing a carbonyl group at the B-ring moiety and subsequent cyclization in a “reverse two-phase strategy”^37–39^ (Fig. 2b (I)). The carbonyl group works as an electron-withdrawing group for the C-ring, while the C-ring possesses electron-donating alkoxyl and alkyl substituents. MacMillan et al. reported that radical cyclization of precursors with electron-withdrawing cyano, ester, or ketone groups on the aromatic ring proceeded^40^. On the other hand, Vanderwal et al. reported that electron-poor aromatic rings with cyano, trifluromethyl, or ester groups were not suitable in their radical cyclization^41^. Zhao and co-workers reported that cationic cyclization of substrates with strong electron-withdrawing cyano or nitro group did not take place^42^. Chandrasekhar et al. attempted a cationic cyclization of a similar skeleton to our substrates with a carbonyl group at the corresponding B-ring moiety and alkoxy groups at the aromatic ring^43^. However, cyclization did not proceed, while cyclization took place in the absence of the carbonyl group. Although we also initially attempted cationic or radical cyclization with precursors with a carbonyl group at the B-ring moiety and alkoxyl/alkyl substituents at the C-ring, all attempts failed to afford desired cyclized products (Fig. 2b (I)).

To solve this problem, we envisioned a reverse cyclization approach for oxygenated tricyclic aromatic diterpenes using a reverse two-phase strategy, in which radical cyclization is initiated from the C-ring (Fig. 2b (II)) in contrast to the biomimetic polyene cyclization initiated from the A-ring moiety. Oxygenated and substituted cyclization precursors with a halogen atom are prepared convergently from substituted phenol derivatives and acid chlorides followed by site-selective halogenation. Employing a photoredox catalyst, the carbon radical generated by scission of the halogen atom of the aromatic ring reacts with the alkene. The cyclization is then expected to directly furnish the tricyclic skeleton of **1a**–**d** with an enone group in the B-ring and substituents on the C-ring. By changing the substituted phenols, **1a**–**d** could be synthesized by a unified synthetic route. Herein, we report the unified eight-step synthesis of oxygenated tricyclic aromatic diterpenes (±)-**1a**–**d** by the reverse radical cyclization with the reverse two-phase strategy. The usefulness of the cyclized intermediate for divergent derivatization to other diterpenes is also reported.

## Results and discussion

### Synthesis of 5,6-dehydrosugiol (1a)

**Fig. 3:**
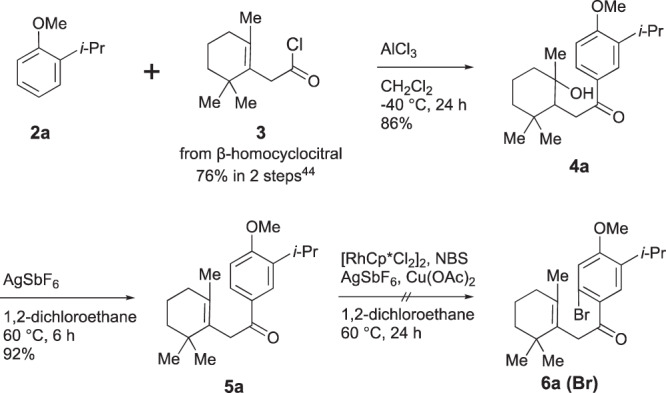
Convergent synthesis of ketone 5a and attempted Rh-catalyzed bromination. Cp*: pentamethylcyclopentadienyl, NBS: *N*-bromosuccinimide.

Our first target was the abietane diterpene, 5,6-dehydrosugiol (**1a**). Common A-ring segment **3** for **1a**–**d** was prepared from β-homocyclocitral in 76% yield through oxidation and chlorination by following a known procedure^44^. Methyl ether **2a** of the C-ring segment was prepared by methylation^45^ of commercially available 2-isopropylphenol in 96% yield (Fig. 3). Friedel-Crafts acylation between **2a** and **3** in the presence of AlCl_3_ at −40 °C, accompanied by hydration, furnished alcohol **4a** as a single diastereomer. The stereochemistry of **4a** was not determined. The alcohol **4a** was dehydrated by a catalytic amount of AgSbF_6_ (0.1 eq.) in 1,2-dichloroethane at 60 °C, giving alkene **5a**. Site-selective halogenation of **5a** was attempted under rhodium-catalyzed conditions reported by Glorius and co-workers^46^, but desired **6a (Br)** was not obtained. Investigation of the conditions and products indicated that the double bond of **6a** reacted with NBS, causing decomposition.

**Fig. 4:**
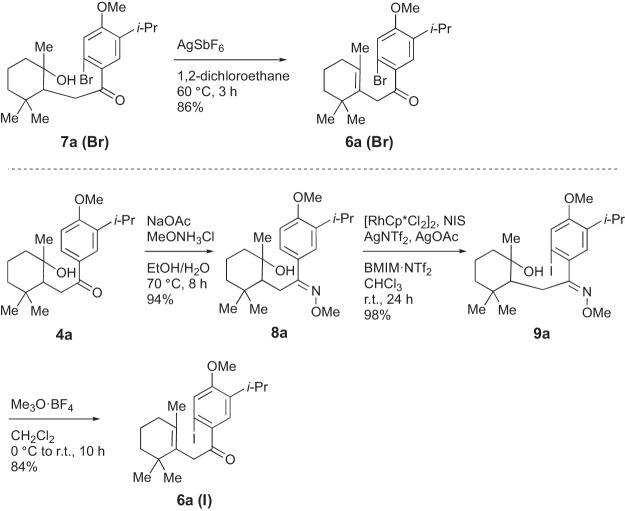
Syntheses of precursors 6a for radical cyclization. Cp*: pentamethylcyclopentadienyl, NIS: *N*-iodosuccinimide. AgNTf_2_: silver bis(trifluoromethanesulfonyl)imide, BMIM · NTf_2_: 1-butyl-3-methylimidazolium bis(trifluoromethanesulfonyl)imide.

To avoid degradation, we screened conditions for the halogenation of alcohol **4a** (Table 1). The conditions of Glorius using [RhCp*Cl_2_]_2_ (2.5 mol%), AgSbF_6_ (10 mol%), and Cu(OAc)_2_ (1.2 eq.) in dichloroethane gave a similar result to the case of **5a** (Table 1, entry 1). In the presence of AgSbF_6_ at 60 °C, dehydration from **4a** to **5a** occurred preferentially. Reported transition metal-catalyzed halogenations using a carbonyl group as a directing group required temperatures higher than 60 °C^47,48^. However, Wu et al. and Du et al. reported that an ionic liquid promoted rhodium-catalyzed C-H cyanation and alkenylation with other directing groups at room temperature^49,50^. Thus, we applied the mixture of an ionic liquid BMIM•NTf_2_ and chloroform as the solvent to the halogenation of **4a** (entry 2), in which the low solubility of **4a** in BMIM•NTf_2_ was improved by chloroform. Under this condition, degradation was not observed, and desired **7a (Br)** was obtained in a 24% yield. Bromination proceeded only at the C9 position, and other regioisomers were not observed. Through examination of brominating reagents, silver catalysts, and carboxylates (entries 2–8), the combination of NBS, AgNTf_2_, and AgOAc was the best, furnishing **7a (Br)** in 55% yield (entry 4). Dehydration of **7a (Br)** under the same conditions as **4a** gave **6a (Br)** an 86% yield (Fig. 4).

**Table 1:** Screening of rhodium-catalyzed halogenation of 4a^a^.


Entry	NXS	Silver cat.	Carboxylate	Yield (%)
1^b^	NBS	AgSbF_6_	Cu(OAc)_2_	-
2	NBS	AgNTf_2_	Cu(OAc)_2_	24
3	NBS	AgNTf_2_	PivOH	-
4	NBS	AgNTf_2_	AgOAc	55
5	NBS	AgSbF_6_	AgOAc	51
6	NBS	AgOTf	AgOAc	38
7	NBP	AgNTf_2_	AgOAc	45
8	DBH	AgNTf_2_	AgOAc	-
9	NIS	AgNTf_2_	AgOAc	26

^a^Reaction conditions: **11a** (0.20 mmol), [RhCp*Cl_2_]_2_ (2.5 mol%), NXS (1.5 eq.), silver cat. (10 mol%), carboxylate (1.2 eq.), BMIM•NTf_2_/CHCl_3_ = 1/2 (0.33 M).

^b^1,2-Dichloroethane was used instead of BMIM•NTf2/CHCl3 at 60 °C.

Iodination with NIS under similar conditions as bromination afforded **7a (I)**, but the yield was decreased to 26% (Table 1, entry 9). To improve the yield, the directing group was altered from a carbonyl group to an oxime ether, which is known to form a stronger interaction with a rhodium catalyst (Fig. 4)^51^. Thus, oxime ether **8a** was obtained from **4a** in 94% yield through condensation with methoxy amine. Under the same reaction conditions as bromination, Rh-catalyzed iodination of **8a** with NIS proceeded smoothly, giving only desired **9a** in 98% yield without regioisomers. Palladium-catalyzed halogenation using an oxime ether as a directing group was also reported but did not work well for this substrate **8a**^52–54^. We also attempted to reuse the rhodium and silver catalysts in the ionic liquid and it was found they could be recycled up to three times with almost the same yields (Supplementary Information). The reaction mechanism of rhodium-catalyzed halogenation was examined by DFT calculations by Glorius and co-workers^55^, and we assume that this halogenation proceeded by the same mechanism.

Deprotection of oxime ether **9a** was first attempted by hydrochloric acid, but the reaction resulted in a complex mixture. The conversion was achieved by treatment with Meerwein reagent^56,57^. Methylation to the oxime salt, deprotection, and dehydration took place in one pot under mild conditions, giving **6a (I)** in 84% yield. The reaction possibly proceeded through intramolecular addition of the hydroxy group to the oxime salt followed by elimination (Supplementary Fig. 1).

With the two precursors **6a (Br)** and **6a (I)** in hand, we investigated their radical cyclization to enone **10a**. Cyclization of **6a** could afford a six-membered ring **10a** and a five-membered ring **11a** depending on the bond-forming position of the double bond (Table 2). Generally, 5-*exo* radical cyclization is kinetically favored over 6-*endo* radical cyclization^58^, but the equilibrium between five- and six-membered rings through neophyl rearrangement is known to alter their ratio^59–61^. Blakey and co-workers reported that the ratio of five- and six-membered rings was controlled by the concentration of hydrogen atom donors that quench radical species after the cyclization process, which is known as reductive radical cyclization^62^. In contrast, we investigated the formation of a six-membered ring over a 5-membered ring under the conditions of redox radical cyclization using photoredox catalysts without hydrogen atom donors, in which oxidative quenching of radical species would furnish the enone **10a** directly as the product.

**Table 2:** Screening of radical cyclization of 6a^a^.


Entry	PC	Base	Solvent	Yield (%)^b^	
				10a	11a
1^c^	Ir-1	KH_2_PO_4_	MeCN	0	0
2	Ir-1	KH_2_PO_4_	MeCN	21	51
3	Ir-1	2,6-lutidine	MeCN	22	66
4	Ir-1	TMEDA	MeCN	16	69
5	Ir-1	DBU	MeCN	33	64
6	Ir-1	TMEDA/DBU	MeCN	0	88
7	Ru	DBU	MeCN	0	0
8	Ir-2	DBU^d^	MeCN	42	46
9	Ir-2	DBU^d^	Benezene	11	trace
10	Ir-2	DBU^d^	MeOH	33	64
11	Ir-2	DBU^d^	DMF	0	0
12	Ir-2	DBU^d^	DMSO	45	28
13	Ir-2	DBU^e^	DMSO	55 (50)	29
14	Ir-2	DBU^f^	DMSO	52	29
15^g^	Ir-2	DBU^d^	DMSO	0	0
16	Ir-2	KH_2_PO_4_	DMSO	0	0

^a^Reaction conditions: **6a** (0.10 mmol), PC (5.0 mol%), base (2 eq.), solvent (0.05 M).

^b^Yields were estimated by ^1^H NMR using an internal standard.

^c^**6a (Br)**.

^d^3 eq.

^e^9 eq.

^f^15 eq.

^g^In the dark.

First, **6a (Br)** was treated with *fac*-Ir(ppy)_3_ (Ir-1, 0.05 eq.)^63,64^ and K_2_HPO_4_ (2 eq.) in acetonitrile under irradiation of blue LED. However, radical cyclization did not proceed at all (Table 2, entry 1). It was estimated that the reduction potential of Ir-1 [Ir(III)*/Ir(IV) = − 1.73 V vs. SCE]^63,64^ was insufficient for the reduction with bromoarene **6a (Br)** (Br-Ph, −2.07 V)^65^. Therefore, the substrate was changed to **6a (I)** (I-Ph, −1. 59 V)^65^. The reaction of **6a (I)** with Ir-1 and K_2_HPO_4_ furnished desired six-membered **10a** in 21% yield as well as five-membered **11a** (the mixture of regioisomers of a double bond) in 51% yield as by-products (entry 2). Since desired **10a** was a minor product, the reaction conditions were screened further. Among the investigated bases, KH_2_PO_4_, 2,6-lutidine, TMEDA, DBU, and TMEDA/DBU (entries 2–6), the best yield (33%) was obtained using DBU (entry 5). With DBU, we then examined photoredox catalysts. The reaction did not proceed at all with Ru(bpy)_3_Cl_2_ possessing relatively high redox potentials [Ru(II)*/Ru(III) = − 0.81 V, Ru(II)*/Ru(I) = +0.77 V]^63,64^ (entry 7). Using Ir[dF(CF_3_)ppy]_2_(dtbpy)PF_6_ (Ir-2) with a reduction potential (−0.89 V) close to Ru(bpy)_3_Cl_2_ but with a lower oxidation potential (+1.21 V)^64^, **10a** and **11a** were furnished in almost equal amounts, 42% and 46% yields (entry 8). Next, with Ir-2, solvents such as benzene, methanol, DMF, and DMSO (entries 8-12) were examined. The reaction in DMSO afforded the highest 45% yield of **10a** and a 28% yield of **11a**. When the amount of DBU was increased to 9 eq., the yield of **10a** was further improved to 55% (50% isolated yield) (entry 13). With 15 eq. of DBU, the yield was similar to that with 9 eq. (entry 14). In the dark without irradiation of light, the reaction did not proceed (entry 15). No reaction with K_2_HPO_4_ (entry 16) implied that DBU was not only a base but was involved in the redox reaction (*vide infra*).

**Fig. 5:**
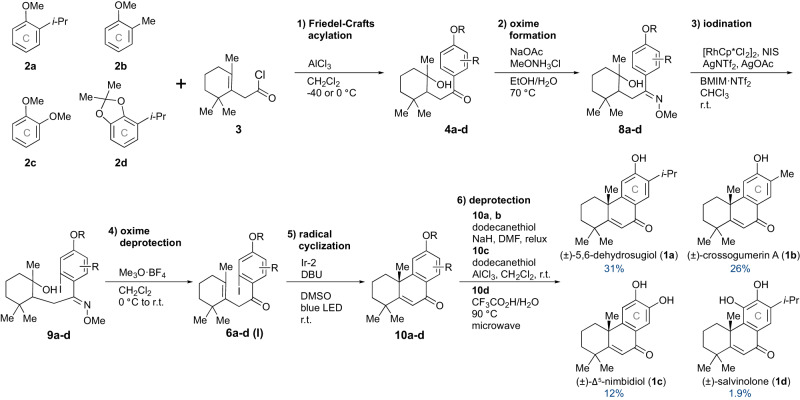
Syntheses of tricyclic aromatic diterpenes (±)-1a–d with different substitution patterns on C-ring. Cp*: pentamethylcyclopentadienyl, NIS: *N*-iodosuccinimide. AgNTf_2_: silver bis(trifluoromethanesulfonyl)imide, BMIM∙NTf_2_: 1-butyl-3-methylimidazolium bis(trifluoromethanesulfonyl)imide, Ir-2: (4,4’-Di-*tert*-butyl-2,2’-bipyridine)bis[3,5-difluoro-2-[5-trifluoromethyl-2-pyridinyl-*κN*)phenyl-*κC*]iridium(III) hexafluorophosphate.

Finally, (±)-5,6-dehydrosugiol (**1a**) was synthesized by deprotection of **10a** using dodecanethiol according to Tada’s method^30,66,67^. The total yield of **1a** was 31% in 6 steps from **2a** (Fig. 5) and 24% in 8 steps from β-homocyclocitral, which was much improved from the reported 13% overall yield in 11 steps^30,35^.

### Syntheses of 1b–d

The established synthetic method for (±)-**1a** was applied to the syntheses of (±)-**1b**–**d** with different substitution patterns on the C-ring (Fig. 5 and Table 3). (±)-Crossogumerin A (**1b**), a podocarpane diterpene, was synthesized by the same synthetic route in 26% overall yield from known methyl ether **2b**^68^, in which the yield of each step was similar to that of **1a**. In the synthesis of (±)-Δ^5^-nimbidiol (**1c**), Friedel-Crafts acylation of commercially available **2c** with **3** was carried out at 0 °C owing to the lower reactivity of **2c**. The yield of **4c** was lowered to 51% accompanied by the formation of by-product **5c** without hydration (alkene **5** in Fig. 3). The yields of steps 2 to 5 were similar to those of **1a**. Our rhodium-catalyzed conditions allowed the site-selective iodination of **9c** in 97% yield, although a methoxy group was reported to be a sterically insufficient substituent to control the reaction site in the rhodium-catalyzed halogenation at high temperature^46^. Deprotection of **10c** was performed by the combination of AlCl_3_ and dodecanethiol reported by Matsumoto et al.^36^, giving **1c** in 60% yield. The total yield of Δ^5^-nimbidiol (**1c**) was 12% from **2c**. (±)-Salvinolone (**1d**), an abietane diterpene, was also synthesized in 1.9% overall yield from our previously synthesized **2d**^68,69^. In the synthesis of **1d**, the yields of steps 1 to 4 were similar to those of **1a**. It is worth noting that our rhodium-catalyzed iodination conditions could distinguish the sterically close C4 and C6 positions completely and iodination proceeded only at the less sterically hindered C4 position. The yield of radical cyclization was slightly lowered because cyclic precursor **6d (I)** underwent deiodination due to the increased steric hindrance on the C-ring. The lower total yield of **1d** compared to those of **1a**–**c** was due to the low yield of deprotection of the acetal on the C-ring. The enone group of the B-ring was reported to be sensitive to acidic conditions^70^. Even after screening various conditions, the deprotection yield of **1d** by trifluoroacetic acid and H_2_O at 90 °C with microwave heating was only 11% with unidentified polar by-products.

**Table 3:** Yields for each step from 2a–d to 1a–d.

	1st	2nd	3rd	4th	5th	6th
	4	8	9	6 (I)	10	1
**1a**	86	94	98	84	50	94
**1b**	77	96	88	84	51	94
**1c**	51	97	97	89	46	60
**1d**	81	93	93	84	36	11

### Derivatization to other diterpenes

**Fig. 6:**
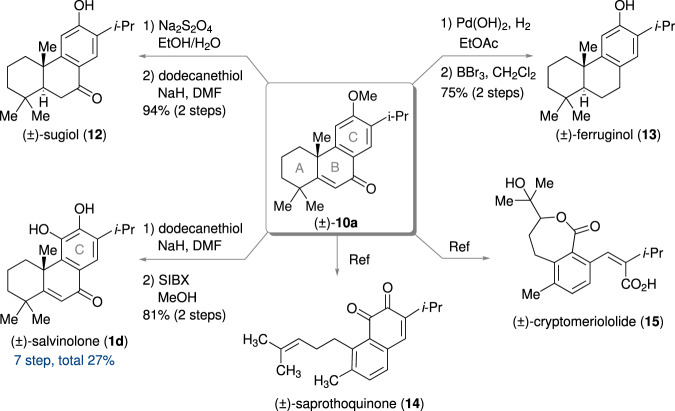
Derivatization of intermediate (±)-10a to other diterpenes. SIBX: stabilized 2-iodoxybenzoic acid with benzoic acid and isophthalic acid.

Tricyclic intermediate (±)-**10a** for **1a** was also useful for divergent syntheses of other diterpenes (Fig. 6) due to its easily convertible structure. Modification of the B-ring afforded (±)-sugiol (**12**)^21,71^ and (±)-ferruginol (**13**)^72^. Using Na_2_S_2_O_4_ in H_2_O/EtOH at reflux^73^, 1,4-reduction of the enone group in the B-ring proceeded selectively. When Pd(OH)_2_ was used under a hydrogen atmosphere in ethyl acetate, reduction of the carbonyl group proceeded in addition to 1,4-reduction^74,75^. These reductions proceeded diastereoselectively. Sugiol (**12**) and ferruginol (**13**) were obtained after demethylation in 94% and 75% yields in 2 steps, respectively. **10a** was also reported as a precursor for saprorthoquinone (**14**)^24,36^ and cryptomeriololide (**15**)^76^. We succeeded in the site-selective hydroxylation on the C-ring of **1a** using stabilized IBX in methanol^41,68,77^, giving (±)-salvinolone (**1d**) in 84% yield from **1a**. While the total yield of **1d** was 1.9% in 6 steps from **2d** in the above synthesis owing to the low yield of deprotection, the total yield of **1d** was much improved to 27% in 7 steps from **2a**.

### Mechanistic consideration of cyclization

**Fig. 7:**
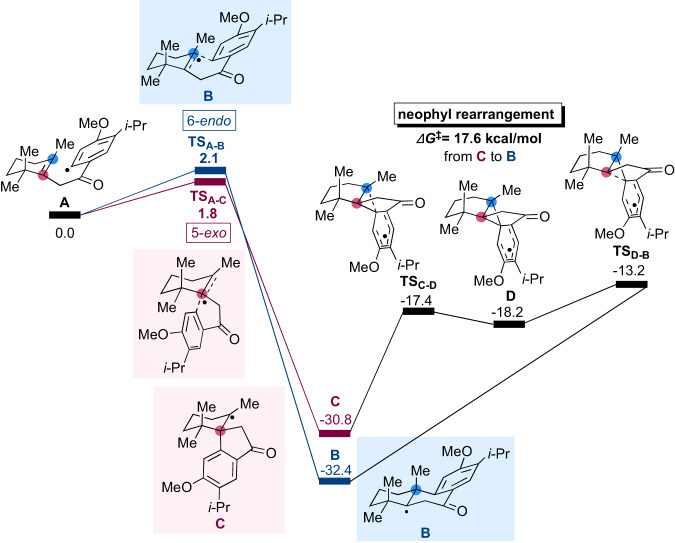
Calculated energy profile of 6-*endo*, 5-*exo* cyclizations, and neophyl rearrangement. UM06-2X/6-311 + + G(d,p) with SMD (DMSO).

To understand the reaction profile of radical cyclization, DFT calculations at the UM06-2X/6-311 + + G(d,p) level of theory with SMD (DMSO) were performed (Fig. 7). The activation barrier (**TS**_**A-B**_) of 6-*endo* cyclization from radical intermediate **A** to six-membered **B** was 2.1 kcal/mol, and the energy of intermediate **B** was −32.4 kcal/mol. The activation barrier [**TS**_**A-C**)_] of 5-*exo* cyclization from **A** to five-membered **C** was 1.8 kcal/mol, which was 0.3 kcal/mol lower than that of 6-*endo* cyclization. The energy of **C** was −30.8 kcal/mol, which was 1.6 kcal/mol higher than that of six-membered **B**. These calculations indicated that 5-*exo* cyclization was kinetically favored over 6-*endo* cyclization, while six-membered **B** was thermodynamically favored over five-membered **C**. Neophyl rearrangement from **C** to **B** was calculated to go through **TS**_**C-D**_, intermediate **D**, and **TS**_**D-B**_. The activation barrier from **C** to **B** was 17.6 kcal/mol, which was reasonable for the rearrangement to occur at the experimental temperature of cyclization (room temperature).

**Fig. 8:**
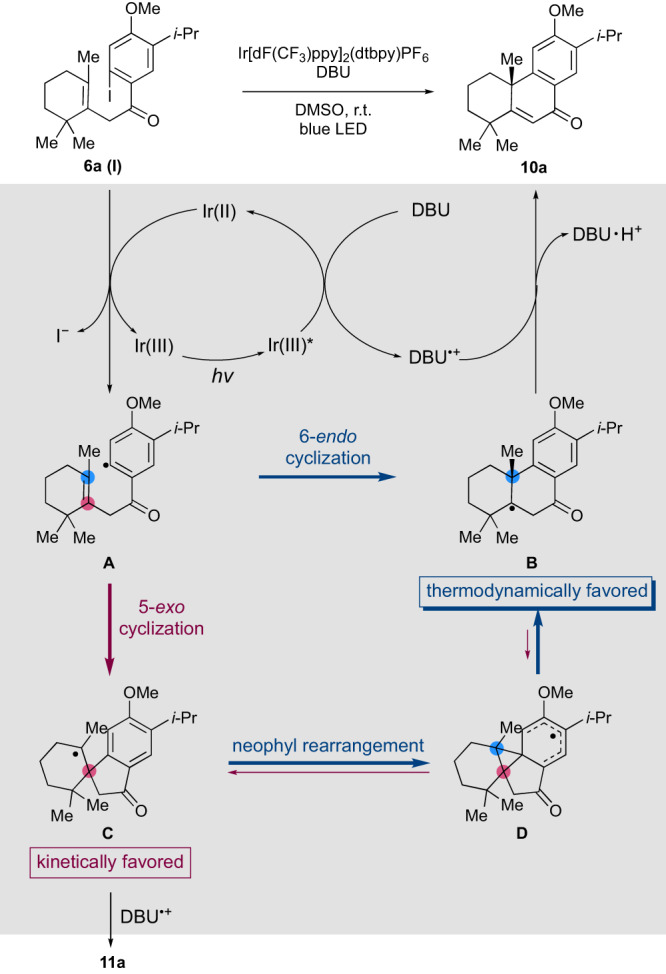
Proposed reaction mechanism of radical cyclization of 6a (I). Ir[dF(CF_3_)ppy]_2_(dtbpy)PF_6_: (4,4’-Di-*tert*-butyl-2,2’-bipyridine)bis[3,5-difluoro-2-[5-trifluoromethyl-2-pyridinyl-*κN*)phenyl-*κC*]iridium(III) hexafluorophosphate, DBU: 1,8-diazabicyclo[5.4.0]undec-7-ene, LED: light emitting diode.

Based on these experimental and calculated results, a mechanism of radical cyclization from **6a (I)** to **10a** and **11a** with Ir[dF(CF_3_)ppy]_2_(dtbpy)PF_6_ and DBU is proposed (Fig. 8). Judging from the redox potentials of excited Ir[dF(CF_3_)ppy]_2_(dtbpy)PF_6_ [Ir(III)*/Ir(IV) = − 0.89 V, Ir(III)*/Ir(II) = +1.21 V vs. SCE]^64^ by light, I-Ph (−1.59 V)^65^, and DBU (+1.28 V)^63^, Ir(III)* does not reduce **6a (I)** but oxidizes DBU to form Ir(II) and DBU^•+^. Iodide **6a (I)** is reduced by Ir(II) to give radical intermediate **A** with regeneration of Ir(III). The lack of reaction with K_2_HPO_4_ (Table 2, entry 16) also supports that Ir(III)* does not directly reduce **6a (I)**. Cyclization of intermediate **A** gives kinetically favored 5-membered **C** as the major product and 6-membered **B** as the minor product. The equilibrium between **C** and **B** through neophyl rearrangement is shifted to the thermodynamically favored 6-membered **B**. Finally, the abstraction of hydrogen from intermediates **B** and **C** by DBU^•+^ ^78,79^ gives **10a** and **11a**. The ratio of **10a** and **11a** would be determined by the combination of the first kinetic ratio of **B** and **C**, the rate of neophyl rearrangement from **C** to **B**, and the rate of oxidation from **B** or **C** to **10a** or **11a** depending on the reaction conditions. Although the details are not clear, it is supposed that the slow oxidative quench from **C** to **11a** and the shift from **C** to **B** allowed the preferential formation of **10a** over **11a** under the optimized conditions.

## Conclusion

We established a new synthetic route to oxygenated tricyclic aromatic diterpenes, (±)-5,6-dehydrosugiol (**1a**), (±)-crossogumerin A (**1b**), (±)-∆^5^-nimbidiol (**1c**), and (±)-salvinolone (**1d**), possessing an enone group in the B-ring and different substitution patterns of the C-ring, based on the reverse cyclization approach from the arene side in a convergent reverse two-phase strategy. The oxygenated and substituted precursors for cyclization were convergently prepared through Friedel-Crafts acylation between A- and C-ring moieties and site-selective iodination. The iodination with exclusive site selectivity was achieved using a rhodium catalyst on an oxime ether in an ionic liquid at room temperature under mild conditions. Radical redox cyclization using an iridium photoredox catalyst and DBU succeeded in furnishing the thermodynamically favored 6-membered product with an enone group in the B-ring preferentially through neophyl rearrangement over the kinetically favored 5-membered product, whose reaction profile was supported by DFT calculations. (±)-5,6-Dehydrosugiol (**1a**), (±)-crossogumerin A (**1b**), (±)-Δ^5^-nimbidiol (**1c**), and (±)-salvinolone (**1d**) were synthesized in only 8 steps from β-homocyclocitral by this synthetic route including deprotection. The syntheses of **1a** and **1d** were much improved from those of previous reports, and these are the first syntheses for **1b** and **1c**. The oxygenated cyclized intermediate **10a** was also useful for divergent derivatization to diterpenes, (±)-sugiol (**12**), (±)-ferruginol (**13**), (±)-saprorthoquinone (**14**), (±)-cryptomeriololide (**15**), and (±)-salvinolone (**1d**). In addition to the biomimetic polyene cyclization in the two-phase strategy, this reverses radical cyclization in the convergent reverse two-phase strategy is expected to become a strong approach for the efficient syntheses of bioactive oxygenated tricyclic aromatic diterpenoids and derivatives.

## Methods

### General procedure for iodination

Oxime ether **8** was dissolved in BMIM∙NTf_2_ and anhydrous dichloromethane (1/1, 0.14 M), and [RhCp*Cl_2_]_2_ (2.5 mol%), silver bis(trifluoromethanesulfonyl)imide (0.1 eq.), silver acetate (1.1 eq.) and NIS (1.2 eq.) were added to the solution at room temperature under argon atmosphere. After stirring at room temperature for 24 h, the reaction mixture was extracted six times with ethyl acetate. The combined extracts were washed with saturated aqueous sodium thiosulfate, saturated aqueous sodium hydrogen carbonate, and brine, dried over anhydrous sodium sulfate, filtered through a cotton plug, and concentrated under reduced pressure. The residue was purified by silica gel column chromatography with hexane/ethyl acetate to afford iodide **9**.

### General procedure for radical cyclization

Iodide **6** was dissolved in anhydrous DMSO (0.05 M) in a J. Young test tube, and Ir[dF(CF_3_)ppy]_2_(dtbpy)PF_6_ (5 mol%) and 1,8-diazabicyclo[5.4.0]undec-7ene (DBU, 9.0 eq.) were added to the solution. The mixture was degassed by three freeze-pump-thaw cycles. The tube was placed 6 cm away from 40 W blue LED lamps (Kessil A160WE Tuna Blue) with a cooling fan blowing air at room temperature to keep the reaction vessel at room temperature. After stirring for 24 h, the reaction was quenched by saturated aqueous ammonium chloride solution and ethyl acetate. The organic layer was separated, and the aqueous layer was extracted twice with ethyl acetate. The combined extracts were washed with water and brine, dried over anhydrous sodium sulfate, filtered through a cotton plug, and concentrated under reduced pressure. The residue was purified by silica gel column chromatography with hexane/ethyl acetate to afford tricyclic compound **10**.

Other experimental procedures, characterization data of compounds, NMR spectra, and reaction coordinates of calculations are included in Supplementary Methods in the Supplementary Information, and Supplementary Data 1 and 2.

### Supplementary information

Supplementary Information

Description of Additional Supplementary Files

Supplementary Data 1

Supplementry Data 2

## Data Availability

All data are included in this article, Supplementary Information, Supplementary Data 1 (NMR spectra), and Supplementary Data 2 (DFT calculations).
