# Supplementary material for: Unified short syntheses of oxygenated tricyclic aromatic diterpenes by radical cyclization with a photoredox catalyst
Source: Commun Chem. 2023 Aug 21;6:169. doi: 10.1038/s42004-023-00979-2 (PMC10442340; doi:10.1038/s42004-023-00979-2)
Supplement: Supplementary file 1 — Supplementary Information [file 42004_2023_979_MOESM1_ESM.pdf]

Supplementary Information

Unified short syntheses of oxygenated tricyclic aromatic diterpenes by radical cyclization with a photoredox catalyst

Riichi Hashimoto,[1]* Kengo Hanaya,[1] Takeshi Sugai,[1] and Shuhei Higashibayashi[1]*

[1]Faculty of Pharmacy, Keio University, 1-5-30 Shibakoen, Minato-ku, Tokyo 105-8512, Japan
E-mail: riichi8222hashimoto@keio.jp, higashibayashi-sh@pha.keio.ac.jp

Table of Contents

Supplementary Figure S1

Supplementary Methods

1. General Information S1
2. Synthesis and Characterization of Compounds S2

Supplementary References S15

Supplementary Figure

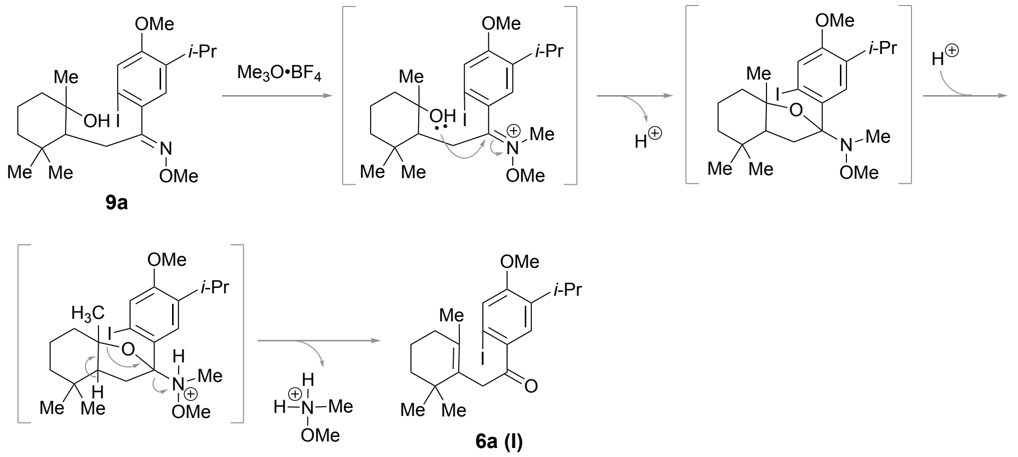

Supplementary Fig. 1 Proposed reaction mechanism of deprotection of oxime ether and dehydration.

Supplementary Methods

1. General Information

Reagents and solvents for syntheses were commercially purchased and air- and/or moisture-sensitive reactions were carried out using dry solvents under an argon atmosphere. Stabilized SIBX (*ca.* 40% IBX with benzoic and isophthalic acid) was purchased from ASTA technologies (United Kingdom). β-Homocyclocitral was purchased from Sigma-Aldrich (W347418). TLC analysis was performed using Merck TLC Silica gel 60 F_{254}. Preparative thin-layer chromatography (PTLC) was conducted using Merck PLC Silica gel 60 F_{254} 0.5 mm. Flash silica gel column chromatography was performed on Wako Wakosil® C-300 or Biotage SNAP Ultra cartridge. Heating reactions were performed with Organic Synthesizer, EYELA ChemiStation, using aluminum heating block with cooling circulator or Biotage Initiator for the microwave condition. Melting points were measured on a Mitamura Riken Kogyo MELTEMP and uncorrected. IR spectra were recorded on a Jasco FT/IR-4700 spectrometer with ATR PRO ONE in ATR mode using diamond prism. ^{1}H and ^{13}C NMR spectra were measured at 500 and 126 MHz on a Bruker spectrometer. $CDCl_3$, DMSO-d_6 and acetone-d_6 were used as solvents and the residual peaks were used as internal standards (^{1}H NMR: $CDCl_3$ 7.26 ppm, acetone-d_6 2.05 ppm; ^{13}C NMR: $CDCl_3$ 77.0 ppm, acetone-d_6 29.8 ppm). High resolution mass spectra were recorded on JEOL JMS-T100LP using electrospray ionization (ESI).

2. Synthesis and Characterization of Compounds

2,6,6-Trimethyl-1-cyclohexenyl-1-acetyl chloride (3)

Acid chloride **3** was prepared in 2 steps from β-homocyclocitral according to the literature.[1] β-homocyclocitral (8.30 g, 50 mmol) and 2-methyl-2-butene (15 mL) were dissolved in *tert*-butyl alcohol (120 mL), and a solution of sodium chlorite (9.05 g, 100 mmol) and sodium dihydrogen phosphate (13.3 g, 85 mmol) in water (50 mL) was added dropwise to the solution at 0 °C over 30 min. After stirring for 3 h at room temperature, the reaction mixture was concentrated *in vacuo* and diluted with ethyl acetate and water. The organic layer was separated, and the aqueous layer was extracted with ethyl acetate three times. The combined extract was washed with water and brine, dried over anhydrous sodium sulfate and filtered through a cotton plug. The filtrate was concentrated *in vacuo* to afford carboxylic acid. The carboxylic acid (9.41 g, 50 mmol) was dissolved in anhydrous dichloromethane (100 mL), and oxalyl chloride (8.6 mL, 100 mmol) was added dropwise to the solution at 0 °C. After stirring for 3 h at room temperature, the solution was concentrated *in vacuo* and the residue was distilled under reduced pressure to give acid chloride **3** (6.47 g, 76% yield in 2 steps) as yellow oil.

^{1}H NMR ($CDCl_3$): δ 3.63 (s, 2H), 2.00-1.98 (m, 2H), 1.60-1.56 (m, 2H), 1.57 (s, 3H), 1.47-1.43 (m, 2H), 0.96 (s, 6H) ppm. ^{13}C NMR ($CDCl_3$): δ 172.6, 134.1, 129.5, 46.9, 38.9, 34.5, 32.6, 27.9 (2C), 20.4, 19.1 ppm. The ^{1}H and ^{13}C NMR spectra were identical to those in the literature.[1]

General procedure A for Friedel-Crafts acylation

Ether **2** and acid chloride **3** were dissolved in anhydrous dichloromethane, and aluminum chloride was added to the solution. After stirring, the reaction was quenched by water and chloroform. The organic layer was separated, and the aqueous layer was extracted with chloroform three times. The combined extract was washed with saturated aqueous sodium hydrogen carbonate solution and brine, dried over anhydrous sodium sulfate, filtered through a cotton plug, and concentrated *in vacuo*. The residue was purified by silica gel column chromatography with hexane/ethyl acetate to afford alcohol **4**.

2-(2-Hydroxy-2,6,6-trimethylcyclohexyl)-1-(3-isopropyl-4-methoxyphenyl)ethan-1-one (4a)

Crude product obtained by General Procedure A at −40 °C for 24 h from ether **2a** (3.32 g, 22 mmol)[2] and acid chloride **3** (3.99 g, 20 mmol) with aluminum chloride (2.92 g, 22 mmol) in dichloromethane (60 mL) was purified by silica gel chromatography with hexane/ethyl acetate (98/2 to 7/3) to afford alcohol **4a** (5.71 g, 86% yield) as yellow prism.

M.p.: 98.1-98.5 °C (recrystallized from diisopropyl ether). IR: ν 3444, 2928, 1667, 1596, 1253, 1170, 814, 756 cm^{-1}. ^{1}H NMR ($CDCl_3$): δ 7.89 (d, *J* = 2.3 Hz, 1H), 7.89 (dd, *J* = 2.3. 8.4 Hz, 1H), 6.87 (d, *J* = 8.4 Hz, 1H), 3.90 (s, 3H), 3.32 (sep, *J* = 6.9 Hz, 1H), 3.09 (dd, *J* = 17.2, 4.2 Hz, 1H), 3.00 (d, *J* = 17.2, 6.3 Hz, 1H), 2.21-2.19 (m, 1H), 2.16-2.04 (m, 1H), 1.87-1.83 (m, 1H), 1.61-1.43 (m, 4H), 1.30 (ddd, *J* = 12.8, 12.8, 3.5 Hz, 1H), 1.24 (d, *J* = 7.0 Hz, 3H), 1.23 (d, *J* = 7.0 Hz, 3H), 1.21 (s, 3H), 0.91 (s, 3H), 0.89 (s, 3H) ppm. ^{13}C NMR ($CDCl_3$): δ 201.1, 160.8, 137.1, 130.0, 128.0, 126.6, 109.5, 73.2, 55.5, 52.0, 43.5, 41.3, 35.4, 34.7, 32.6, 26.9, 22.6, 22.5 (2C), 21.5, 20.6 ppm. HRMS (ESI)(*m/z*) for $C_{21}H_{32}NaO_3$ ($M+Na^+$): Calculated 355.2244, found 355.2225.

2-(2-Hydroxy-2,6,6-trimethylcyclohexyl)-1-(4-methoxy-3-methylphenyl)ethan-1-one (4b)

Crude product obtained by General Procedure A at −40 °C for 18 h from ether **2b** (728 mg, 6.0 mmol)[3] and acid chloride **3** (1.06 g, 5.5 mmol) with aluminum chloride (798 mg, 6.0 mmol) in anhydrous dichloromethane (25 mL) was purified by silica gel chromatography with hexane/ethyl acetate (9/1 to 8/2) to afford alcohol **4b** (1.19 g, 74% yield) as colorless needle. M.p.: 100.5-101.1 °C (recrystallized from diisopropyl ether/hexane = 1/2). IR: ν 3427, 2926, 1666, 1600, 1504, 1257, 1139, 1026, 915, 816, 733 cm^{-1}. ^{1}H NMR ($CDCl_3$): δ 7.87 (dd, *J* = 7.5, 1.5 Hz, 1H), 7.80 (d, *J* = 1.5 Hz, 1H), 6.83 (d, *J* = 7.5 Hz, 1H), 3.87 (s, 3H), 3.05 (dd, *J* = 14.4, 3.7 Hz, 1H), 2.97 (dd, *J* = 14.4, 5.0 Hz, 1H), 2.23 (s, 3H), 2.19 (t, *J* = 4.9 Hz, 1H), 2.14 (br, 1H), 1.82 (brd, *J* = 10.4 Hz, 1H), 1.56-1.39 (m, 4H), 1.27 (ddd, *J* = 10.9, 10.9, 3.1 Hz, 1H), 1.18 (s, 3H), 0.89 (s, 3H), 0.85 (s, 3H) ppm. ^{13}C NMR ($CDCl_3$): δ 200.8, 161.7, 130.8, 129.8, 128.3, 126.7, 109.2, 73.1, 55.5, 51.9, 43.5, 41.3, 35.4, 34.7, 32.6, 22.6, 21.5, 20.6, 16.3 ppm. HRMS (ESI)(*m/z*) for $C_{19}H_{28}NaO_3$ ($M+Na^+$): Calculated 327.1931, found 327.1914.

2-(2-Hydroxy-2,6,6-trimethylcyclohexyl)-1-(3,4-dimethoxylphenyl)ethan-1-one (4c)

Crude product obtained by General Procedure A at 0 °C for 17 h from ether **2c** (827 mg, 6.0 mmol) and acid chloride **3** (1.07 g, 5.5 mmol) with aluminum chloride (800 mg, 6.0 mmol) in anhydrous dichloromethane (25 mL) was purified by silica gel chromatography with hexane/ethyl acetate (9/1 to 7/3) to afford alcohol **4c** (889 mg, 51% yield) as colorless crystal.

M.p.: 92.2-92.8 °C (recrystallized from diisopropyl ether/hexane = 1/2). IR: ν 3435, 2930, 1666, 1585, 1514, 1264, 1152, 1024, 733 cm^{-1}. ^{1}H NMR ($CDCl_3$): δ 7.64 (dd, *J* = 8.5, 2.0 Hz, 1H), 7.52 (d, *J* = 2.0 Hz, 1H), 6.86 (d, *J* = 8.5 Hz, 1H), 3.91 (s, 3H), 3.90 (s, 3H), 3.03 (dd, *J* = 17.0, 4.8 Hz, 1H), 2.98 (dd, *J* = 17.0, 5.8 Hz, 1H), 2.17 (t, *J* = 5.1 Hz, 1H), 2.03 (br, 1H), 1.82-1.79 (m, 1H), 1.55-1.37 (m, 4H), 1.26 (ddd, *J* = 12.9, 12.9, 3.5Hz, 1H), 1.17 (s, 3H), 0.87 (s, 3H), 0.84 (s, 3H) ppm. ^{13}C NMR ($CDCl_3$): δ 200.7, 153.2, 149.0, 130.4, 122.7, 110.6, 110.0, 73.2, 56.0 (2C), 52.2, 43.4, 41.2, 35.4, 34.7, 32.6, 22.6, 21.5, 20.6 ppm. HRMS (ESI)(*m/z*) for $C_{19}H_{28}NaO_4$ ($M+Na^+$): Calculated 343.1880, found 343.1881.

2-(2-Hydroxy-2,6,6-trimethylcyclohexyl)-1-(7-isopropyl-2,2-dimethylbenzo-1,3-dioxol-5-yl)ethan-1-one (4d)

Crude product obtained by General Procedure A at −40 °C for 18 h from acetal **2d** (2.12 g, 11 mmol)[3,4] and acid chloride **3** (2.00 g, 10 mmol) with aluminum chloride (1.46 g, 11 mmol) in anhydrous dichloromethane (30 mL) was purified by silica gel chromatography with hexane/ethyl acetate (9/1 to 8/2) to afford alcohol **4d** (2.71 g, 73% yield) as yellow oil.

IR: ν 3437, 2928, 1668, 1434, 1297, 1173, 915, 734 cm^{-1}. ^{1}H NMR ($CDCl_3$): δ 7.49 (d, *J* = 1.6 Hz, 1H), 7.26 (d, *J* = 1.6 Hz, 1H), 3.05-2.98 (m, 3H), 2.20 (t, *J* = 5.7 Hz, 1H), 2.16 (br, 1H), 1.83 (brd, *J* = 10.9 Hz, 1H), 1.69 (s, 6H), 1.58-1.40 (m, 5H), 1.28 (d, *J* = 7.0 Hz, 6H), 1.20 (s, 3H), 0.90 (s, 3H), 0.88 (s, 3H) ppm. ^{13}C NMR ($CDCl_3$): δ 200.5, 149.0, 147.3, 131.2, 129.2, 121.5, 118.7, 106.0, 73.1, 52.1, 43.4, 41.3, 35.4, 34.8, 32.6, 28.8, 25.9 (2C), 22.6, 22.0 (2C), 21.5, 20.6 ppm. HRMS (ESI)(*m/z*) for $C_{23}H_{34}NaO_4$ ($M+Na^+$): Calculated 397.2350, found 397.2342.

1-(3-Isopropyl-4-dimethoxylphenyl)-2-(2,6,6-trimethylcyclohex-1-en-1-yl)ethan-1-one (5a)

Alcohol **4a**, (66.5 mg, 0.20 mmol) was dissolved in anhydrous 1,2-dichloroethane (2.0 mL), and silver hexafluoroantimonate (6.8 mg, 0.02 mmol) was added to the solution. After stirring for 6 h at 60 °C, the reaction was quenched by filtration through a pad of silica gel. The filtrate was concentrated *in vacuo*, and the residue was purified by silica gel column chromatography with hexane/ethyl acetate (98/2 to 10/1) to afford alcohol **5a** (58.9 mg, 92% yield) as yellow oil.

IR: ν 2958, 1682, 1598, 1253, 1170 cm^{-1}. ^{1}H NMR ($CDCl_3$): δ 7.91 (d, *J* = 2.3 Hz, 1H), 7.89 (dd, *J* = 2.3, 8.5 Hz, 1H), 6.87 (d, *J* = 8.5 Hz, 1H), 3.90 (s, 3H), 3.69 (s, 2H), 3.32 (sep, *J* = 6.9 Hz, 1H), 2.04 (brt, *J* = 6.3 Hz, 2H), 1.66-1.63 (m, 2H), 1.52-1.49 (m, 2H), 1.49 (s, 3H), 1.24 (d, *J* = 6.9 Hz, 6H), 0.94 (s, 6H) ppm. ^{13}C NMR ($CDCl_3$): δ 196.8, 160.6, 137.1, 130.9, 130.8, 130.2, 127.6, 126.3, 109.5, 55.5, 39.3, 37.6, 34.6, 32.6, 28.2 (2C), 26.9, 22.4 (2C), 20.6, 19.5 ppm. HRMS (ESI)(*m*/*z*) for $C_{21}H_{30}NaO_2$ ($M+Na^+$): Calculated 337.2139, found 337.2159.

1-(2-Bromo-5-isopropyl-4-dimethoxylphenyl)-2-(2-hydroxy-2,6,6-trimethylcyclohexyl)ethan-1-one (7a (Br))

Alcohol **4a (**330 mg, 1.0 mmol) was dissolved in 1-butyl-3-methylimidazolium bis(trifluoromethylsulfonyl)imide (BMIM·NTf_2, 1.0 mL) and anhydrous chloroform (2.0 mL), and (pentamethylcyclopentadienyl)rhodium(III)dimer [$(RhCp^*Cl_2)_2$, 15.4 mg, 0.025 mmol], silver bis(trifluoromethanesulfonyl)imide (38.9 mg, 0.10 mmol), silver acetate (184 mg, 1.1 mmol) and NBS (270 mg, 1.5 mmol) were added to the solution at room temperature under argon atmosphere. After stirring for 24 h at room temperature, the reaction mixture was extracted with diethyl ether six times. The combined extract was washed with saturated aqueous sodium thiosulfate solution, saturated aqueous sodium hydrogen carbonate solution and brine, dried over anhydrous sodium sulfate, filtered through a cotton plug, and concentrated *in vacuo*. The residue was purified by silica gel column chromatography with hexane/ethyl acetate (98/2 to 4/1) to afford bromide **7a (Br)** (226 mg, 55% yield) as yellow oil and starting material **4a** (72.6 mg, 22%).

IR: ν 3468, 2930, 1682, 1593, 1240, 1033, 913, 733 cm^{-1}. ^{1}H NMR ($CDCl_3$): δ 7.43 (s, 1H), 7.03 (s, 1H), 3.85 (s, 3H), 3.24 (sep, *J* = 6.9 Hz, 1H), 3.07 (dd, *J* = 17.4, 6.2 Hz, 1H), 2.99 (dd, *J* = 17.4, 4.5 Hz, 1H), 2.12 (dd, *J* = 6.2, 4.5 Hz, 1H), 1.85-1.81 (m, 1H), 1.69 (br, 1H), 1.69-1.22 (m, 5H), 1.19 (d, *J* = 6.9 Hz, 3H), 1.18 (d, *J* = 6.9 Hz, 3H), 1.18 (s, 3H), 0.91 (s, 3H), 0.85 (s, 3H) ppm. ^{13}C NMR ($CDCl_3$): δ 204.0, 158.8, 136.2, 133.1, 127.6, 117.7, 115.9, 73.2, 55.8, 52.7, 43.5, 41.2, 39.2, 35.3, 32.7, 26.8, 22.6, 22.3 (2C), 21.5, 20.6 ppm. HRMS (ESI)(*m*/*z*) for $C_{21}H_{31}{}^{79}BrNaO_3$ ($M+Na^+$): Calculated 433.1349, found 433.1326.

2-(2-Hydroxy-2,6,6-trimethylcyclohexyl)-1-(2-iodo-5-isopropyl-4-dimethoxylphenyl)ethan-1-one (7a (I))

Alcohol **4a** (66.3 g, 0.20 mmol) was dissolved in BMIM·NTf_2 (0.50 mL) and anhydrous chloroform (1.0 mL). $[RhCp^*Cl_2]_2$ (3.1 mg, 5.0 μmol), silver bis(trifluoromethanesulfonyl)imide (7.8 mg, 0.020 mmol), silver acetate (36.5 mg, 0.22 mmol) and *N*-iodosuccinimide (NIS, 67.7 mg, 0.30 mmol) were added to the solution at room temperature under argon atmosphere. After stirring for 24 h at room temperature, the reaction mixture was extracted with diethyl ether six times. The combined extract was washed with saturated aqueous sodium thiosulfate solution, saturated aqueous sodium hydrogen carbonate

solution and brine, dried over anhydrous sodium sulfate, filtered through a cotton plug, and concentrated *in vacuo*. The residue was purified by silica gel column chromatography with hexane/ethyl acetate (98/2 to 4/1) to afford iodide **7a (I)** (35.8 mg, 26% yield) as yellow oil and starting material **4a** (27.0 mg, 41%).

IR: ν 3420, 2928, 1676, 1587, 1298, 1240, 1172, 1029, 914, 732 cm^{-1}. ^{1}H NMR ($CDCl_3$): δ 7.47 (s, 1H), 7.33 (s, 1H), 3.84 (s, 3H), 3.24 (sep, *J* = 6.8 Hz, 1H), 3.05 (dd, *J* = 17.2, 6.4 Hz, 1H), 2.91 (dd, *J* = 17.2, 4.4 Hz, 1H), 2.12 (dd, *J* = 6.4, 4.4 Hz, 1H), 1.84-1.81 (m, 1H), 1.63 (br, 1H), 1.60-1.25 (m, 5H), 1.20 (d, *J* = 6.8 Hz, 3H), 1.19 (d, *J* = 6.8 Hz, 3H), 1.18 (s, 3H), 0.92 (s, 3H), 0.86 (s, 3H) ppm. ^{13}C NMR ($CDCl_3$): δ 203.8, 158.6, 136.8, 135.4, 126.9, 123.2, 89.9, 73.3, 55.8, 52.8, 43.5, 41.2, 38.0, 35.3, 32.8, 26.9, 22.6, 22.3, 22.2, 21.5, 20.6 ppm. HRMS (ESI)(*m*/*z*) for $C_{21}H_{31}INaO_3$ ($M+Na^+$): Calculated 481.1211, found 481.1223.

1-(2-Bromo-5-isopropyl-4-dimethoxylphenyl)-2-(2-hydroxy-2,6,6-trimethylcyclohexyl)ethan-1-one (6a (Br))

Alcohol **7a (Br)** (211 mg, 0.51 mmol) was dissolved in anhydrous 1,2-dichloroethane (2.0 mL), and silver hexafluoroantimonate (18.1 mg, 0.050 mmol) was added to the solution. After stirring for 3 h at 60 °C, the reaction was quenched by filtration through a pad of silica gel. The filtrate was concentrated *in vacuo*, and the residue was purified by silica gel column chromatography with hexane/ethyl acetate (98/2 to 10/1) to afford bromide **6a (Br)** (173 mg, 86% yield) as yellow oil.

IR: ν 2926, 1698, 1593, 1239, 1034 cm^{-1}. ^{1}H NMR ($CDCl_3$): δ 7.32 (s, 1H), 7.01 (s, 1H), 3.84 (s, 3H), 3.67 (s, 2H), 3.23 (sep, *J* = 6.9 Hz, 1H), 2.02 (brt, *J* = 6.1 Hz, 2H), 1.63-1.60 (m, 2H), 1.54 (s, 3H), 1.50-1.47 (m, 2H), 1.18 (d, *J* = 6.9 Hz, 6H), 0.93 (s, 6H) ppm. ^{13}C NMR ($CDCl_3$): δ 200.6, 158.6, 136.3, 133.9, 131.5, 130.6, 126.9, 117.1, 115.6, 55.8, 42.3, 39.2, 34.6, 32.6, 28.2 (2C), 26.8, 22.3 (2C), 20.7, 19.4 ppm. HRMS (ESI)(*m*/*z*) for $C_{21}H_{29}{}^{79}BrNaO_2$ ($M+Na^+$): Calculated 415.1244, found 415.1236.

General procedure B for oxime formation

Alcohol **4** was dissolved in ethanol, and a solution of sodium acetate and *O*-methylhydroxylamine hydrochloride in water was added to the solution at room temperature. After stirring for 8 h at 70 °C, the reaction was quenched by aqueous 2 M hydrochloric acid and ethyl acetate. The organic layer was separated, and the aqueous layer was extracted with ethyl acetate. The combined extract was washed with saturated aqueous sodium hydrogen carbonate solution and brine, dried over anhydrous sodium sulfate, filtered through a cotton plug, and concentrated *in vacuo*. The residue was purified by silica gel column chromatography with hexane/ethyl acetate to afford oxime ether **8**.

(*E*)-2-(2-Hydroxy-2,6,6-trimethylcyclohexyl)-1-(3-isopropyl-4-methoxylphenyl)ethan-1-one *O*-methyl oxime (8a).

Crude product obtained by General Procedure B from alcohol **4a** (2.65 g, 8.0 mmol) using sodium acetate (2.88 g, 35 mmol) and *O*-methylhydroxylamine (1.80 g, 22 mmol) in ethanol (25 mL) and water (50 mL) was purified by silica gel chromatography with hexane/ethyl acetate (98/2 to 8/2) to afford oxime ether **8a** (2.72 g, 94% yield) as white amorphous.

IR: ν 3446, 2931, 1499, 1461, 1247, 1046, 913, 880, 814, 733 cm^{-1}. ^{1}H NMR ($CDCl_3$): δ 7.40 (d, *J* = 2.3 Hz, 1H), 7.39 (dd,

J = 2.3. 8.2 Hz, 1H), 6.82 (d, J = 8.2 Hz, 1H), 3.98 (s, 3H), 3.83 (s, 3H), 3.31 (sep, J = 7.0 Hz, 1H), 3.08 (dd, J = 14.3, 7.0 Hz, 1H), 2.57 (dd, J = 14.3, 5.2 Hz, 1H), 2.07 (br, 1H), 1.70-1.67 (m, 1H), 1.53-1.38 (m, 3H), 1.32-1.19 (m, 2H), 1.29 (s, 3H), 1.20 (d, J = 7.0 Hz, 3H), 1.19 (d, J = 7.0 Hz, 3H), 1.04 (ddd, J = 13.3, 13.3, 4.3 Hz, 1H), 0.90 (s, 3H), 0.86 (s, 3H) ppm. ^{13}C NMR ($CDCl_3$): δ 162.4, 157.6, 136.7, 128.0, 126.2, 125.2, 110.2, 73.8, 61.6, 55.4, 54.4, 42.8, 41.8, 35.7, 32.7, 26.8, 23.2, 23.1, 22.7, 22.6, 21.2, 20.2 ppm. HRMS (ESI)(m/z) for $C_{22}H_{35}NNaO_3$ ($M+Na^+$): Calculated 384.2510, found 384.2518.

(*E*)-2-(2-Hydroxy-2,6,6-trimethylcyclohexyl)-1-(4-methoxy-3-methylphenyl)ethan-1-one *O*-methyl oxime (8b).

Crude product obtained by General Procedure B from alcohol **4b** (452 mg, 1.5 mmol) using sodium acetate (540 mg, 6.6 mmol) and *O*-methylhydroxylamine (340 mg, 4.1 mmol) in ethanol (5.0 mL) and water (10 mL) was purified by silica gel chromatography with hexane/ethyl acetate (98/2 to 8/2) to afford oxime ether **8b** (482 mg, 96% yield) as colorless viscous oil.

IR: ν 2928, 1506, 1458, 1248, 1048, 871, 815 cm^{-1}. ^{1}H NMR ($CDCl_3$): δ 7.39 (d, J = 2.1 Hz, 1H), 7.38 (dd, J = 2.1. 7.2 Hz, 1H), 6.79 (d, J = 7.2 Hz, 1H), 3.98 (s, 3H), 3.83 (s, 3H), 3.04 (dd, J = 14.3, 6.9 Hz, 1H), 2.76 (d, J = 14.3, 5.3 Hz, 1H), 2.21 (s, 3H), 2.05 (br, 1H), 1.70-1.67 (m, 2H), 1.52-1.38 (m, 3H), 1.32-1.21 (m, 1H), 1.26 (s, 3H), 1.12 (ddd, J = 12.6, 12.6, 3.9 Hz, 1H), 0.91 (s, 3H), 0.87 (s, 3H) ppm. ^{13}C NMR ($CDCl_3$): δ 162.1, 158.5, 129.7, 127.8, 126.7, 126.0, 109.6, 73.8, 61.6, 55.3, 54.4, 42.8, 41.9, 35.7, 32.7, 23.2, 23.1, 21.2, 20.2, 16.3 ppm. HRMS (ESI)(m/z) for $C_{20}H_{31}NNaO_3$ ($M+Na^+$): Calculated 356.2197, found 356.2179.

(*E*)-2-(2-Hydroxy-2,6,6-trimethylcyclohexyl)-1-(3,4-dimethoxylphenyl)ethan-1-one *O*-methyl oxime (8c).

Crude product obtained by General Procedure B from alcohol **4c** (487 mg, 1.5 mmol) using sodium acetate (547 mg, 6.6 mmol) and *O*-methylhydroxylamine (340 mg, 4.1 mmol) in ethanol (5.0 mL) and water (10 mL) was purified by silica gel chromatography with hexane/ethyl acetate (98/2 to 8/2) to afford oxime ether **8c** (507 mg, 97% yield) as white needle.

M.p.: 98.0-99.0 °C (recrystallized from diisopropyl ether/hexane = 1/2). IR: ν 2929, 1515, 1254, 1047, 1026, 731 cm^{-1}. ^{1}H NMR ($CDCl_3$): δ 7.17 (d, J = 2.0 Hz, 1H), 7.15 (dd, J = 2.0. 8.2 Hz, 1H), 6.83 (d, J = 8.2 Hz, 1H), 3.97 (s, 3H), 3.87 (s, 3H), 3.86 (s, 3H), 3.01 (dd, J = 14.4, 6.4 Hz, 1H), 2.81 (d, J = 14.3, 5.9 Hz, 1H), 1.94 (br, 1H), 1.69-1.66 (m, 1H), 1.52 (t, J = 6.2 Hz, 1H), 1.48-1.34 (m, 2H), 1.30-1.22 (m, 2H), 1.25 (s, 3H), 1.10 (ddd, J = 13.2, 13.2, 4.1 Hz, 1H), 0.90 (s, 3H), 0.85 (s, 3H) ppm. ^{13}C NMR ($CDCl_3$): δ 161.7, 149.8, 148.8, 128.5, 120.1, 110.8, 110.4, 73.8, 61.7, 55.9 (2C), 54.2, 43.0, 41.9, 35.7, 32.6, 23.3, 22.8, 21.2, 20.2 ppm. HRMS (ESI)(m/z) for $C_{20}H_{31}NNaO_4$ ($M+Na^+$): Calculated 372.2146, found 372.2132.

(*E*)-2-(2-Hydroxy-2,6,6-trimethylcyclohexyl)-1-[7-isopropyl-2,2-dimethylbenzo(1,3)dioxol-5-yl]ethan-1-one *O*-methyl oxime (8d).

Crude product obtained by General Procedure B from alcohol **4d** (560 mg, 1.5 mmol) using sodium acetate (542 mg, 6.6 mmol) and *O*-methylhydroxylamine (340 mg, 4.1 mmol) in ethanol (5.0 mL) and water (10 mL) was purified by silica gel chromatography with hexane/ethyl acetate (98/2 to 8/2) to afford oxime ether **8d** (485 mg, 81% yield) as colorless

amorphous.

IR: ν 2929, 1433, 1228, 1042 cm^{-1}. ^1H NMR ($CDCl_3$): δ 6.91 (d, *J* = 1.5 Hz, 1H), 6.86 (d, *J* = 1.5 Hz, 1H), 3.97 (s, 3H), 3.06-2.97 (m, 2H), 2.72 (d, *J* = 14.3, 5.2 Hz, 1H), 2.02 (br, 1H), 1.70-1.667 (m, 1H), 1.71-1.67 (m, 1H), 1.66 (s, 3H), 1.65 (s, 3H), 1.53 (dd, *J* = 7.1, 5.3 Hz, 1H), 1.47-1.27 (m, 5H), 1.28 (s, 3H), 1.23 (d, *J* = 7.0 Hz, 3H), 1.21 (d, *J* = 7.0 Hz, 3H), 1.15 (ddd, *J* = 13.3, 13.3, 4.1 Hz, 1H), 0.90 (s, 3H), 0.88 (s, 3H) ppm. ^{13}C NMR ($CDCl_3$): δ 162.4, 147.3, 145.7, 129.2, 128.9, 118.5, 117.7, 105.6, 73.8, 61.6, 54.4, 42.8, 41.9, 35.7, 32.7, 28.6 25.9 (2C), 23.2, 23.1, 22.2 (2C), 21.2, 20.3 ppm. HRMS (ESI)(*m*/*z*) for $C_{24}H_{37}NNaO_4$ ($M+Na^+$): Calculated 426.2615, found 426.2628.

General Procedure C for iodination.

Oxime ether **8** was dissolved in BMIM·NTf_2 and anhydrous chloroform. $[RhCp^*Cl_2]_2$, silver bis(trifluoromethanesulfonyl)imide, silver acetate and NIS were added to the solution at room temperature under argon atmosphere. After stirring at room temperature, the reaction mixture was extracted with diethyl ether six times. The combined extract was washed with saturated aqueous sodium thiosulfate, saturated aqueous sodium hydrogen carbonate and brine, dried over anhydrous sodium sulfate, filtered through a cotton plug, concentrated *in vacuo*. The residue was purified by silica gel column chromatography with hexane/ethyl acetate to afford iodide **9**.

(*E*)-2-(2-Hydroxy-2,6,6-trimethylcyclohexyl)-1-(2-iodo-5-isopropyl-4-methoxylphenyl)ethan-1-one *O*-methyl oxime (9a)

Crude product obtained by General Procedure C from oxime ether **8a** (2.52 g, 7.0 mmol) using $[RhCp^*Cl_2]_2$ (108 mg, 0.18 mmol), silver bis(trifluoromethanesulfonyl)imide (272 mg, 0.70 mmol), silver acetate (1.29 g, 7.7 mmol) and NIS (1.89 g, 8.4 mmol) in BMIM·NTf_2 (25 mL) and chloroform (25 mL) was purified by silica gel chromatography with hexane/ethyl acetate (98/2 to 8/2) to afford iodide **9a** (3.32 g, 98% yield) as yellow amorphous.

IR: ν 3474, 2959, 1487, 1307, 1234, 1037, 670 cm^{-1}. ^1H NMR ($CDCl_3$): δ 7.23 (s, 1H), 7.16 (s, 1H), 4.00 (s, 3H), 3.80 (s, 3H), 3.24 (sep, *J* = 6.9 Hz, 1H), 3.12 (dd, *J* = 14.3, 7.7 Hz, 1H), 3.11 (s, 1H), 2.38 (dd, *J* = 14.3, 2.5 Hz, 1H), 1.74-1.71 (m, 1H), 1.52-1.28 (m, 5H), 1.30 (s, 3H), 1.18 (d, *J* = 6.9 Hz, 3H), 1.17 (d, *J* = 6.9 Hz, 3H), 1.04 (ddd, *J* = 13.1, 13.1, 3.9 Hz, 1H), 0.78 (s, 3H), 0.51 (s, 3H) ppm. ^{13}C NMR ($CDCl_3$): δ 163.3, 156.0, 136.0, 131.5, 128.0, 120.2, 92.0, 72.2, 60.8, 54.7, 52.9, 40.8, 40.4, 34.3, 31.4, 25.5, 24.4, 22.8, 21.4, 21.3, 19.8, 19.1 ppm. HRMS (ESI)(*m*/*z*) for $C_{22}H_{34}INNaO_3$ ($M+Na^+$): Calculated 510.1476, found 510.1475.

(*E*)-2-(2-Hydroxy-2,6,6-trimethylcyclohexyl)-1-(2-iodo-4-methoxy-5-methylphenyl)ethan-1-one *O*-methyl oxime (9b)

Crude product obtained by General Procedure C from oxime ether **8b** (333 mg, 1.0 mmol) using $[RhCp^*Cl_2]_2$ (15.3 mg, 0.025 mmol), silver bis(trifluoromethanesulfonyl)imide (38.7 mg, 0.10 mmol), silver acetate (182 mg, 1.1 mmol) and NIS (276 mg, 1.2 mmol) in BMIM·NTf_2 (3.0 mL) and chloroform (3.0 mL) was purified by silica gel chromatography with hexane/ethyl acetate (98/2 to 8/2) to afford iodide **9b** (406 mg, 88% yield) as colorless amorphous.

IR: ν 3474, 2933, 1595, 1491, 1461, 1438, 1306, 1237, 1035, 730 cm^{-1}. ^{1}H NMR ($CDCl_3$): δ 7.18 (d, *J* = 0.8 Hz, 1H), 7.09 (d, *J* = 0.8 Hz, 1H), 3.97 (s, 3H), 3.77 (s, 3H), 3.14 (s, 1H), 3.09 (dd, *J* = 14.3, 8.0 Hz, 1H), 2.45 (dd, *J* = 14.3, 2.3 Hz, 1H), 2.13 (s, 3H), 1.70-1.67 (m, 1H), 1.49-1.26 (m, 5H), 1.29 (s, 3H), 1.07 (ddd, *J* = 13.0, 13.0, 3.7 Hz, 1H), 0.76 (s, 3H), 0.54 (s, 3H) ppm. ^{13}C NMR ($CDCl_3$): δ 164.5, 158.1, 133.2, 132.3, 126.7, 120.8, 92.7, 73.3, 61.8, 55.6, 54.0, 41.7, 41.3, 35.4, 32.5, 25.5, 23.8, 20.8, 20.1, 15.8 ppm. HRMS (ESI)(*m/z*) for $C_{20}H_{30}INNaO_3$ (M+Na$^+$): Calculated 482.1163, found 482.1165.

(*E*)-2-(2-Hydroxy-2,6,6-trimethylcyclohexyl)-1-(2-iodo-4,5-dimethoxylphenyl)ethan-1-one *O*-methyl oxime (9c)

Crude product obtained by General Procedure C from oxime ether **8c** (352 mg, 1.0 mmol) using $[RhCp^*Cl_2]_2$ (15.6 mg, 0.025 mmol), silver bis(trifluoromethanesulfonyl)imide (39.1 mg, 0.10 mmol), silver acetate (184 mg, 1.1 mmol) and NIS (273 mg, 1.2 mmol) in BMIM·NTf_2 (3.0 mL) and chloroform (3.0 mL) was purified by silica gel chromatography with hexane/ethyl acetate (98/2 to 8/2) to afford iodide **9c**(460 mg, 97% yield) as colorless prism.

M.p.: 133.5-135.9 °C (recrystallized from diisopropyl ether/hexane = 1/2). IR: ν 3454, 2931, 1501, 1382, 1248, 1208, 1028, 730 cm^{-1}. ^{1}H NMR ($CDCl_3$): δ 7.23 (s, 1H), 6.93 (s, 1H), 4.00 (s, 3H), 3.86 (s, 6H), 3.12 (dd, *J* = 14.4, 8.2 Hz, 1H), 2.95 (s, 1H), 2.48 (dd, *J* = 14.4, 2.4 Hz, 1H), 1.73-1.70 (m, 1H), 1.52-1.28 (m, 5H), 1.31 (s, 3H), 1.07 (ddd, *J* = 13.1, 13.1, 3.8 Hz, 1H), 0.79 (s, 3H), 0.58 (s, 3H) ppm. ^{13}C NMR ($CDCl_3$): δ 164.5, 149.5, 148.9, 132.9, 121.7, 114.8, 84.8, 73.6, 61.9, 56.2 (2C), 54.1, 41.9, 41.4, 35.4, 32.5, 25.4, 23.8, 20.8, 20.1 ppm. HRMS (ESI)(*m/z*) for $C_{20}H_{30}INNaO_4$ (M+Na$^+$): Calculated 498.1112, found 498.1108.

(*E*)-2-(2-Hydroxy-2,6,6-trimethylcyclohexyl)-1-[4-iodo-7-isopropyl-2,2-dimethylbenzo(1,3)dioxol-5-yl]ethan-1-one *O*-methyl oxime (9d)

Crude product obtained by General Procedure C from oxime ether **8d** (403 mg, 1.0 mmol) using $[RhCp^*Cl_2]_2$ (15.2 mg, 0.025 mmol), silver bis(trifluoromethanesulfonyl)imide (39.1 mg, 0.10 mmol), silver acetate (185 mg, 1.1 mmol) and NIS (277 mg, 1.2 mmol) in BMIM·NTf_2 (3.0 mL) and chloroform (3.0 mL) was purified by silica gel chromatography with hexane/ethyl acetate (98/2 to 8/2) to afford iodide **9d** (488 mg, 93% yield) as orange amorphous.

IR: ν 3469, 2932, 1428, 1377, 1214, 1039, 856, 733 cm^{-1}. ^{1}H NMR ($CDCl_3$): δ 6.72 (s, 1H), 3.98 (s, 3H), 3.14 (s, 1H), 3.09 (dd, *J* = 14.3, 7.6 Hz, 1H), 2.94 (sep, *J* = 6.9 Hz, 1H), 2.25 (dd, *J* = 14.3, 2.5 Hz, 1H), 1.71-1.69 (m, 1H), 1.69 (s, 3H), 1.66 (s, 3H), 1.51-1.28 (m, 5H), 1.28 (s, 3H), 1.21 (d, *J* = 6.9 Hz, 3H), 1.19 (d, *J* = 6.9 Hz, 3H), 1.06 (ddd, *J* = 12.9, 12.9, 3.8 Hz, 1H), 0.77 (s, 3H), 0.53 (s, 3H) ppm. ^{13}C NMR ($CDCl_3$): δ 163.7, 149.2, 143.5, 131.9, 129.2, 122.6, 117.8, 73.2, 70.5, 61.8, 54.1, 41.8, 41.5, 35.4, 32.5, 28.3, 25.9, 25.8, 25.5, 23.9, 22.0 (2C), 20.8, 20.1 ppm. HRMS (ESI)(*m/z*) for $C_{24}H_{36}INNaO_4$ (M+Na$^+$): Calculated 552.1582, found 552.1600.

Reuse of rhodium catalyst, silver catalyst, ionic liquid and for synthesis of 9a

Oxime ether **8a** (121 mg, 0.33 mmol) was dissolved in BMIM·NTf_2 (1.0 mL) and anhydrous chloroform (1.0 mL). $[RhCp^*Cl_2]_2$ (5.2 mg, 8.3 μmol) silver bis(trifluoromethanesulfonyl)imide (13.0 mg, 0.033 mmol), silver acetate (61.2 mg, 0.36 mml) and NIS (90.2 mg, 0.40 mmol) were added to the solution at room temperature under argon atmosphere. After stirring at room temperature for 18 h, the reaction mixture was extracted with ethyl acetate six times. The ionic liquid layer

was used for the reaction in the 2nd cycle. The ethyl acetate layer was washed with saturated aqueous sodium thiosulfate, saturated aqueous sodium hydrogen carbonate and brine, dried over anhydrous sodium sulfate, filtered through a cotton plug, concentrated *in vacuo*. The residue was purified by silica gel column chromatography with hexane/ethyl acetate (98/2 to 8/2) to afford iodide **9a** (149 mg, 92% yield, 1st cycle). In the 2nd cycle, the ionic liquid layer was concentrated *in vacuo*. Oxime ether **8a** (120 mg, 0.33 mmol) and chloroform (1.0 mL) were added followed by NIS (89.8 mg, 0.40 mmol). The same procedure as described above gave iodide **9a** (143 mg, 88% yield, 2nd cycle). In the 3rd cycle, the same procedure using oxime ether **8a** (121 mg, 0.33 mmol), chloroform (1.0 mL) and NIS (90.5 mg) furnished iodide **9a** (144 mg, 89% yield, third cycle).

General Procedure D for deprotection of oxime ether and dehydration

Oxime ether **9** was dissolved in anhydrous dichloromethane. Trimethyloxonium tetrafluoroborate (Meerwein reagent) was added to the solution at 0 °C under argon atmosphere. After stirring for 10 h at room temperature, the reaction mixture was quenched by saturated aqueous ammonium chloride solution and dichloromethane. The organic layer was separated, and the aqueous layer was extracted with dichloromethane twice. The combined extract was washed with saturated aqueous sodium hydrogen carbonate solution and brine, dried over anhydrous sodium sulfate, filtered through a cotton plug, and concentrated *in vacuo*. The residue was purified by silica gel column chromatography with hexane/ethyl acetate to afford iodide **6 (I)**.

1-(2-Iodo-5-isopropyl-4-dimethoxylphenyl)-2-(2,6,6-trimethylcyclohexy-1-en-1-yl)ethan-1-one (6a (I))

Crude product obtained by General Procedure D from oxime ether **9a** (2.20 g, 4.5 mmol) using Meerwein reagent (737 mg, 5.0 mmol) in anhydrous dichloromethane (50 mL) was purified by silica gel chromatography with hexane/ethyl acetate (99/1 to 95/5) to afford oxime ether **6a (I)** (1.67 g, 84% yield) as yellow oil.

IR: ν 2926, 1695, 1588, 1484, 1316, 1240, 1171, 1029, 845 cm^{-1}. ^1H NMR ($CDCl_3$): δ 7.37 (s, 1H), 7.32 (s, 1H), 3.84 (s, 3H), 3.64 (s, 2H), 3.25 (sep, *J* = 6.9 Hz, 1H), 2.04 (brt, *J* = 6.3 Hz, 2H), 1.66-1.60 (m, 2H), 1.55 (s, 3H), 1.51-1.49 (m, 2H), 1.20 (d, *J* = 6.9 Hz, 6H), 0.95 (s, 6H). ^{13}C NMR ($CDCl_3$): δ 200.5, 158.5, 136.9, 136.3, 131.6, 130.4, 126.1, 122.9, 89.2, 55.8, 41.2, 39.2, 34.6, 32.6, 28.2 (2C), 26.9, 22.2 (2C), 20.7, 19.4 ppm. HRMS (ESI)(*m/z*) for $C_{21}H_{29}INaO_2$ ($M+Na^+$): Calculated 463.1105 found 463.1120.

1-(2-Iodo-4-methoxy-4-methylphenyl)-2-(2,6,6-trimethylcyclohexy-1-en-1-yl)ethan-1-one (6b (I))

Crude product obtained by General Procedure D for from oxime ether **9b** (460 mg, 1.0 mmol) using Meerwein reagent (163 mg, 1.1 mmol) in anhydrous dichloromethane (10 mL) was purified by silica gel chromatography with hexane/ethyl acetate (94/6) to afford oxime ether **6b (I)** (347 mg, 84% yield) as yellow oil.

IR: ν 2924, 1693, 1591, 1486, 1437, 1244, 1150, 1031, 651, 484 cm^{-1}. ^1H NMR ($CDCl_3$): δ 7.35 (d, *J* = 0.8 Hz, 1H), 7.31 (d, *J* = 0.8 Hz, 1H), 3.84 (s, 3H), 3.63 (s, 2H), 2.19 (s, 3H), 2.04 (brt, *J* = 6.3 Hz, 2H), 1.65-1.60 (m, 2H), 1.54 (s, 3H), 1.51-1.48 (m, 2H), 0.94 (s, 6H) ppm. ^{13}C NMR ($CDCl_3$): δ 199.8, 159.4, 135.7, 131.5, 130.4, 130.3, 126.6, 122.6 (2C), 89.3, 55.8, 41.0, 39.2, 34.5, 32.6, 28.2, 20.7, 19.4, 16.1 ppm. HRMS (ESI)(*m/z*) for $C_{19}H_{25}INaO_2$ ($M+Na^+$): Calculated 435.0792,

found 435.0811.

1-(2-Iodo-4,5-dimethoxyphenyl)-2-(2,6,6-trimethylcyclohexy-1-en-1-yl)ethan-1-one (6c (I))

Crude product obtained by general General Procedure D from oxime ether **9c** (474 mg, 1.0 mmol) using Meerwein reagent (163 mg, 1.1 mmol) in anhydrous dichloromethane (10 mL) was purified by silica gel chromatography with hexane/ethyl acetate (94/6) to afford oxime ether **6c (I)** (382 mg, 89% yield) as colorless needle.

M.p.: 97.5-98.2 °C (recrystallized from diisopropyl ether/hexane = 1/2). IR: ν 2927, 1696, 1587, 1500, 1256, 1163, 1026, 732 cm^{-1}. ^{1}H NMR ($CDCl_3$): δ 7.30 (s, 1H), 7.00 (s, 1H), 3.88 (s, 3H), 3.87 (s, 3H), 3.65 (s, 2H), 2.01 (brt, *J* = 6.3 Hz, 2H), 1.62-1.58 (m, 2H), 1.54 (s, 3H), 1.49-1.46 (m, 2H), 0.93 (s, 6H) ppm. ^{13}C NMR ($CDCl_3$): δ 200.3, 151.0, 148.8, 136.7, 131.7, 130.3, 123.2, 111.5, 81.1, 56.3, 56.1, 41.3, 39.2, 34.6, 32.6, 28.2 (2C), 20.7, 19.4 ppm. HRMS (ESI)(*m*/*z*) for $C_{19}H_{25}INaO_3$ ($M+Na^+$): Calculated 451.0741, found 451.0742.

1-[2-Iodo-7-isopropyl-2,2-dimethylbenzo(1,3)dioxol-5-yl]-2-(2,6,6-trimethylcyclohexy-1-en-1-yl)ethan-1-one (6d (I)).

Crude product obtained by General Procedure D from oxime ether **9d** (531 mg, 1.0 mmol) using Meerwein reagent (163 mg, 1.1 mmol) in anhydrous dichloromethane (10 mL) was purified by silica gel chromatography with hexane/ethyl acetate (94/6) to afford oxime ether **6d (I)** (447 mg, 84% yield) as yellow oil.

IR: ν 2928, 1691, 1426, 1197, 1006, 849, 789, 516 cm^{-1}. ^{1}H NMR ($CDCl_3$): δ 7.03 (s, 1H), 3.64 (s, 2H), 2.98 (sep, *J* = 7.0 Hz, 1H), 2.04 (brt, *J* = 6.3 Hz, 2H), 1.72 (s, 6H), 1.64-1.60 (m, 2H), 1.55 (s, 3H), 1.51-1.49 (m, 2H), 1.25 (d, *J* = 7.0 Hz, 6H), 0.95 (s, 6H) ppm. ^{13}C NMR ($CDCl_3$): δ 199.6, 150.1, 145.3, 136.0, 131.5, 130.5, 128.8, 121.0, 118.3, 66.9, 41.0, 39.2, 34.5, 32.6, 28.7, 28.2 (2C), 26.1 (2C), 21.8 (2C), 20.7, 19.4 ppm. HRMS (ESI)(*m*/*z*) for $C_{23}H_{31}INaO_3$ ($M+Na^+$): Calculated 505.1211, found 505.1219.

General Procedure E for radical cyclization

Iodide **6 (I)** was dissolved in anhydrous DMSO in a J. Young test tube, and Ir[dF(CF_3)ppy]$_2$(dtbpy)PF_6 and 1,8-diazabicyclo[5.4.0]undec-7ene (DBU) were added to the solution. The mixture was degassed by three freeze-pump-thaw cycles. The tube was placed at 6 cm away from 40 W blue LED lamps (Kessil A160WE Tuna Blue) with a cooling fan blowing air at room temperature to keep the reaction vessel at ambient temperature. After stirring for 24 h at room temperature, the reaction was quenched by saturated aqueous ammonium chloride solution and ethyl acetate. The organic layer was separated, and the aqueous layer was extracted with ethyl acetate twice. The combined extract was washed with water and brine, dried over anhydrous sodium sulfate, filtered through a cotton plug, and concentrated *in vacuo*. NMR yields were determined at this stage using nitromethane as an internal standard in $CDCl_3$. The residue was purified by silica gel column chromatography with hexane/ethyl acetate to afford tricyclic compound **10**.

5,6-Dehydrosugiol methyl ether (10a)

Crude product was obtained by General Procedure E from iodide **6a (I)** (44.1 mg, 0.10 mmol) using Ir[dF(CF_3)ppy]$_2$(dtbpy)PF_6 (5.8 mg, 5.0 μmol) and DBU (135 μL, 0.90 mmol) in DMSO (2.0 mL). **10a**, *endo*-**11a** with an

endo double bond, and *exo*-**11a** with an *exo* double bond were estimated to be 55%, 13%, and 16% yields by ^{1}H NMR of the crude product. The crude product was purified by silica gel chromatography with hexane/ethyl acetate (98/2 to 85/15) to afford tricyclic compound **10a** (15.5 mg, 50% yield) as colorless solid.

10a: ^{1}H NMR ($CDCl_3$): δ 7.99 (s, 1H), 6.86 (s, 1H), 6.46 (s, 1H), 3.90 (s, 3H), 3.29 (sep, *J* = 6.9 Hz, 1H), 2.46-2.43 (m, 1H), 2.09-2.01 (m, 1H), 1.77-1.65 (m, 3H), 1.54 (s, 3H), 1.43 (ddd, *J* = 13.6, 13.6, 4.0 Hz, 1H), 1.36 (s, 3H), 1.27 (s, 3H), 1.25 (d, *J* = 6.9 Hz, 3H), 1.22 (d, *J* = 6.9 Hz, 3H) ppm. ^{13}C NMR ($CDCl_3$): δ 185.2, 172.7, 160.8, 153.8, 136.0, 124.6, 124.1, 123.5, 105.6, 55.4, 41.4, 40.3, 37.9, 37.5, 32.7, 32.5, 29.2, 26.6, 22.6, 22.4, 18.7 ppm. The ^{1}H and ^{13}C NMR spectra were identical to those in the literature.[2-4]

Endo-**11a** and *exo*-**11a** were inseparable. For characterization, the mixture was converged to *endo*-**11a** according to the cobalt-catalyzed olefin isomerization by Shenvi *et al.*[5] The mixture (31.0 mg) of *endo*-**11** and *exo*-**11a** with unidentified by-products was dissolved in benzene in a J. Young test tube. Co(salen$^{t\text{-Bu}}$,$^{t\text{-Bu}}$)Cl (6.7 mg, 0.010 mmol) was added to the solution. After the mixture was degassed by three freeze-pump-thaw cycles, phenysilane (3.5 μL, 0.025 mmol) was added to the solution under argon atmosphere. After stirring for 24 h at 40 °C under argon atmosphere, the reaction mixture was filtered through a pad of silica gel and concentrated *in vacuo*. The residue was purified by preparative TLC (hexane/ethyl acetate = 10/1) to afford *endo*-**11a** (6.7 mg, 22%).

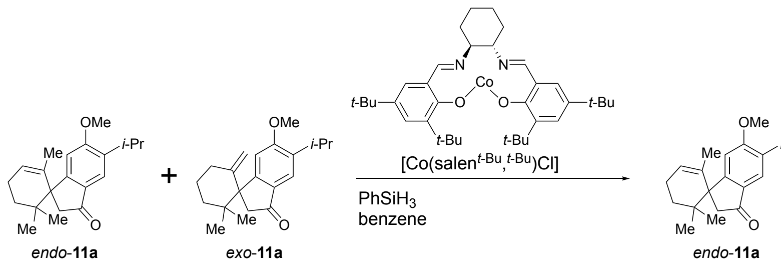

***endo*-11a**: IR: ν 2960, 1703, 1606, 1280, 1237, 1060 cm^{-1}. ^{1}H NMR ($CDCl_3$): δ 7.56 (s, 1H), 6.74 (s, 1H), 5.63-5.61 (m, 1H), 3.88 (s, 3H), 3.26 (sep, *J* = 7.0 Hz, 1H), 2.75 (d *J* = 18.8 Hz,1H), 2.57 (d, *J* = 18.8 Hz,1H), 2.18-2.16 (m, 1H), 1.69-1.51 (m, 3H), 1.43 (q, *J* = 1.9 Hz, 3H), 1.22 (d, *J* = 7.0 Hz, 3H), 1.20 (d, *J* = 7.0 Hz, 3H), 0.81 (s, 3H), 0.77 (s, 3H) ppm. ^{13}C NMR ($CDCl_3$): δ 205.2, 161.8, 137.8, 135.7, 131.1, 123.9, 120.3 (2C), 108.3, 55.6, 53.8, 36.1, 34.1, 26.8 (2C), 25.2, 24.9, 22.8, 22.5 (2C), 20.4 ppm. HRMS (ESI)(*m/z*) for $C_{21}H_{28}NaO_2$ (M+Na$^+$): Calculated 335.1987, found 335.1973.

Crossogumerin A methyl ether (10b)

Crude product was obtained by General Procedure E from iodide **6b (I) (**41.4 mg, 0.10 mmol) using Ir[dF(CF_3)ppy]$_2$(dtbpy)PF_6 (5.8 mg, 5.0 μmol) and DBU (135 μL, 0.90 mmol) in DMSO (2.0 mL). **10b**, *endo*-**11b** and *exo*-**11b** were estimated to be 53%, 12%, and 13% yields by ^{1}H NMR of the crude product. The crude product was purified by silica gel chromatography with hexane/ethyl acetate (98/2 to 85/15) to afford tricyclic compound **10b** (11.7 mg, 51% yield) as colorless prism.

M.p.: 133.5-134.2 °C (recrystallized from diisopropyl ether/hexane = 1/2). IR: ν 2930, 1650, 1602, 1496, 1456, 1340, 1245, 440 cm^{-1}. ^{1}H NMR ($CDCl_3$): δ 7.91 (d, *J* = 1.0 Hz, 1H), 6.84 (s, 1H), 6.45 (s, 1H), 3.90 (s, 3H), 2.45-2.42 (m, 1H), 2.23 (d, *J* = 0.6 Hz, 3H), 2.09-2.00 (m, 1H), 1.77-1.63 (m, 3H), 1.53 (s, 3H), 1.44 (ddd, *J* = 13.4, 13.4, 4.1 Hz, 1H), 1.35 (s, 3H),

1.26 (s, 3H) ppm. ^{13}C NMR ($CDCl_3$): δ 185.1, 172.8, 161.7, 154.2, 128.2, 125.7, 124.5, 123.3, 105.2, 55.4, 41.4, 40.3, 37.9, 37.5, 32.7, 32.5, 29.2, 18.7, 15.7 ppm. HRMS (ESI)(*m*/*z*) for $C_{19}H_{24}NaO_2$ ($M+Na^+$): Calculated 307.1669, found 307.1646.

Δ^5-Nimbidiol dimethyl ether (10c)

Crude product (28.2 mg, 56% of **10c** 11% of **11c**, 13% of **11c'**) obtained by General Procedure E from iodide **6b (I) (**43.0 mg, 0.10 mmol) using Ir[dF(CF_3)ppy]$_2$(dtbpy)PF_6 (5.8 mg, 5.0 μmol) and DBU (135 μL, 0.90 mmol) in DMSO (2.0 mL). **10c**, *endo*-**11c** and *exo*-**11c** were estimated to be 56%, 11%, and 13% yields by ^{1}H NMR of the crude product. The crude product was purified by silica gel chromatography with hexane/ethyl acetate (98/2 to 85/15) to afford tricyclic compound **10c** (13.9 mg, 46% yield) as yellow amorphous.

IR: ν 2931, 1646, 1597, 1415, 1265, 874, 503, 475 cm^{-1}. ^{1}H NMR ($CDCl_3$): δ 7.58 (s, 1H), 6.90 (s, 1H), 6.47 (s, 1H), 3.95 (s, 3H), 2.43-2.41 (m, 1H), 2.06-2.00 (m, 1H), 1.77-1.59 (m, 3H), 1.53 (s, 3H), 1.44 (ddd, *J* = 13.4, 13.4, 4.0 Hz, 1H), 1.35 (s, 3H), 1.27 (s, 3H) ppm. ^{13}C NMR ($CDCl_3$): δ 184.8, 173.2, 153.1, 148.6, 147.9, 124.3, 123.9, 107.0, 106.8, 56.1, 56.0, 41.3, 40.4, 38.0, 37.6, 32.7, 32.1, 29.0, 18.7 ppm. HRMS (ESI)(*m*/*z*) for $C_{19}H_{24}NaO_3$ ($M+Na^+$): Calculated 323.1618, found 323.1603.

4-Isopropyl-2,2,8,8,11a-pentamethyl-9,10,11,11a-tetrahydrophenanthro[1,3]dioxol-6(*8H*)-one (10d)

Crude product obtained by General Procedure E from iodide **6d (I) (**48.0 mg, 0.10 mmol) using Ir[dF(CF_3)ppy]$_2$(dtbpy)PF_6 (5.8 mg, 5.0 μmol) and DBU (135 μL, 0.90 mmol) in DMSO (2.0 mL). **10d**, *endo*-**11d** and *exo*-**11d** were estimated to be 40%, 16%, and 11% yields by ^{1}H NMR of the crude product. The crude product was purified by silica gel chromatography with hexane/ethyl acetate (98/2 to 85/15) to afford tricyclic compound **10d** (12.6 mg, 36% yield) as colorless prism.

M.p.: 140.9-141.6 °C (recrystallized from diisopropyl ether/hexane = 1/2). IR: ν 2962, 1657, 1626, 1375, 1317, 1256, 405 cm^{-1}. ^{1}H NMR ($CDCl_3$): δ 7.67 (d, *J* = 0.7 Hz, 1H), 6.40 (s, 1H), 3.03 (sep, *J* = 6.9 Hz, 1H), 2.93-2.91 (m, 1H), 1.98-19.3 (m, 1H), 1.71 (s, 3H), 1.71 (s, 3H), 1.68-1.63 (m, 2H), 1.57 (s, 3H), 1.47-1.39 (m, 2H), 1.34 (s, 3H), 1.27 (d, *J* = 7.0 Hz, 3H), 1.26 (d, *J* = 7.0 Hz, 3H), 1.25 (s, 3H) ppm. ^{13}C NMR ($CDCl_3$): δ 185.0, 172.5, 148.9, 142.7, 133.3, 129.1, 124.4, 124.0, 118.6, 118.0, 40.8 (2C), 37.8, 35.0, 32.9, 28.9, 28.4, 26.1, 25.9 (2C), 22.0, 21.9, 18.5 ppm. HRMS (ESI)(*m*/*z*) for $C_{23}H_{30}NaO_3$ ($M+Na^+$): Calculated 377.2088, found 377.2069.

(±)-5,6-Dehydrosugiol (1a).

Sodium hydride (51.9 mg, 2.2 mmol and 1-dodecanethiol (525 μL, 2.2 mmol) were dissolved in anhydrous DMF (1.0 mL), and a solution of **10a** (62.1 mg, 0.20 mmol) in anhydrous DMF (1.0 mL) was added to the solution at room temperature. After stirring for 8 h at reflux, the reaction was quenched by saturated aqueous ammonium chloride solution at 0 °C and ethyl acetate. The organic layer was separated, and the aqueous layer was extracted with ethyl acetate. The combined extract was washed with brine, dried over anhydrous sodium sulfate, filtered through a cotton plug, and concentrated *in vacuo*. The residue was purified by silica gel column chromatography with hexane/ethyl acetate (98/2 to 7/3) to afford 5,6-dehydrosugiol (**1a**) (55.3 mg, 94% yield) as colorless solid.

^{1}H NMR ($CDCl_3$): δ 8.35 (brs, 1H), 8.03 (s, 1H), 6.99 (s, 1H), 6.47 (s, 1H), 3.29 (sep, *J* = 6.9 Hz, 1H), 2.27 (brd, *J* = 10.9

Hz, 1H), 1.98-1.90 (m, 1H), 1.67-1.63 (m, 2H), 1.53 (ddd, J = 13.2, 13.2, 3.8 Hz, 1H), 1.45 (s, 3H), 1.39-1.35 (m, 1H), 1.30 (s, 3H), 1.26 (d, J = 6.9 Hz, 3H), 1.24 (d, J = 6.9 Hz, 3H), 1.21 (s, 3H) ppm. ^{13}C NMR ($CDCl_3$): δ 186.1, 174.6, 159.1, 154.6, 134.4, 124.9, 124.1, 122.7, 111.2, 41.3, 40.3, 37.6 (2C), 32.6, 32.5, 29.1, 26.9, 22.5, 22.3, 18.6 ppm. The ^{1}H and ^{13}C NMR spectra were identical to those in the literature.[6]

(±)-Crossogumerin A (1b)

Sodium hydride (51.9 mg, 2.2 mmol) and 1-dodecanethiol (525 μL, 2.2 mmol) were dissolved in anhydrous DMF (1.0 mL), and a solution of **10b** (56.9 mg, 0.20 mmol) in anhydrous DMF (1.0 mL) was added to the solution. After stirring for 8 h at reflux, the reaction was quenched by saturated aqueous ammonium chloride solution and ethyl acetate at 0 °C. The organic layer was separated, and the aqueous layer was extracted with ethyl acetate twice. The combined extract was washed with brine, dried over anhydrous sodium sulfate, filtered through a cotton plug, and concentrated *in vacuo*. The residue was purified by silica gel column chromatography with hexane/ethyl acetate (98/2 to 7/3) to afford crossogumerin A (**10b**) (50.9 mg, 94% yield) as colorless solid.

^{1}H NMR ($CDCl_3$): δ 7.93 (d, J = 0.7 Hz, 1H), 6.87 (s, 1H), 6.45 (s, 1H), 2.39-2.35 (m, 1H), 2.89 (d, J = 0.5 Hz, 3H), 2.07-1.98 (m, 1H), 1.76-1.61 (m, 3H), 1.51 (s, 3H), 1.44 (ddd, J = 13.5, 13.5, 4.0 Hz, 1H), 1.35 (s, 3H), 1.26 (s, 3H) ppm. ^{13}C NMR ($CDCl_3$): δ 185.0, 173.1, 158.1, 154.5, 129.1, 124.5, 123.9, 122.6, 110.7, 41.1, 40.3, 37.7, 37.5, 32.6, 32.5, 29.2, 18.6, 15.2 ppm. The ^{1}H and ^{13}C NMR spectra were identical to those in the literature.[7]

(±)-Δ^5-Nimbidiol (1c).

Tricyclic compound **10c** (30.1 mg, 0.10 mmol) and 1-dodecanethiol (120 μL, 1.0 mmol) were dissolved in anhydrous dichloromethane (2.0 mL), and aluminum chloride (80.1 mg, 0.60 mmol) was added to the solution at room temperature. After stirring for 24 h at room temperature, 1-dodecanethiol (60 μL, 0.50 mmol) and aluminum chloride (39.2 mg, 0.30 mmol) were added. After stirring for 24 h, the reaction was quenched by water and chloroform. The organic layer was separated, and the aqueous layer was extracted with chloroform twice. The combined extract was washed with brine, dried over anhydrous sodium sulfate, filtered through a cotton plug, and concentrated *in vacuo*. The residue was purified by silica gel column chromatography with hexane/ethyl acetate (9/1 to 7/3) to afford Δ_5-nimbidiol (**1c**) (16.4 mg, 60% yield) as yellow amorphous.

^{1}H NMR (acetone-d_6): 8.52-8.13 (br, 1H), 7.45 (s, 1H), 7.02 (s, 1H), 6.27 (s, 1H), 2.45-2.42 (m, 1H), 1.72-1.68 (m, 2H), 1.53-1.48 (m, 1H), 1.49 (s, 3H), 1.43 (ddd, J = 12.8, 12.8, 3.6 Hz, 1H), 1.35 (s, 3H), 1.28 (br, 1H), 1.24 (s, 3H) ppm. ^{13}C NMR (acetone-d_6): δ 184.2, 172.9, 151.1, 148.8, 144.7, 124.8, 124.3, 112.3, 111.7, 41.6, 41.1, 38.7, 37.9, 33.0, 32.7, 19.3 ppm. The ^{1}H and ^{13}C NMR spectra were identical to those in the literature.[8]

(±)-Salvinolone (1d)

Tricyclic compound **10d** (35.3 mg, 0.10 mmol) was dissolved in trifluoroacetic acid (0.9 mL) and water (0.1 mL). After stirring for 24 h at 90 °C by a microwave synthesizer, the reaction was quenched by ice and ethyl acetate. The organic layer was separated, the aqueous layer was extracted with ethyl acetate twice. The combined extract was washed with water

three times and brine, dried over anhydrous sodium sulfate, filtered through a cotton plug, and concentrated *in vacuo*. The residue was purified by silica gel column chromatography with hexane/ethyl acetate (4/1) to afford salvinolone (**1d**) (3.5 mg, 11% yield) as colorless solid.

^{1}H NMR ($CDCl_3$): δ 7.70 (s, 1H), 6.46 (s, 1H), 3.27-3.23 (m, 1H), 3.01 (sep, *J* = 6.9 Hz, 1H), 1.97-1.91 (m, 1H), 1.73-1.68 (m, 1H), 1.65 (s, 3H), 1.65-1.58 (m, 1H), 1.47-1.38 (m, 2H), 1.34 (s, 3H), 1.30 (d, *J* = 6.9 Hz, 3H), 1.28 (d, *J* = 6.9 Hz, 3H), 1.25 (s, 3H) ppm. ^{13}C NMR ($CDCl_3$): δ 185.5, 174.9, 144.3, 141.2, 137.3, 132.2, 124.3, 123.6, 115.7, 42.1, 40.4, 38.1, 34.3, 33.1, 29.4, 27.5, 25.0, 22.6, 22.5, 18.7 ppm. The ^{1}H and ^{13}C NMR spectra were identical to those in the literatures [6]

(±)-Salvinolone (1d) from 5,6-dehydrosugiol (1a)

5,6-Dehydrosugiol **1a** (29.8 mg, 0.10 mmol) was dissolved in methanol (5.0 mL), and SIBX (*ca.* 40% purity, 93.1 mg, 0.15 mmol) was added to the solution at 0 °C. After stirring for 6 h at 0 °C, the reaction was quenched by saturated aqueous sodium thiosulfate solution and ethyl acetate. The organic layer was separated, and the aqueous layer was extracted with ethyl acetate three times. The combined extract was washed with saturated aqueous sodium hydrogen carbonate solution and brine, dried over anhydrous sodium sulfate, filtered through a cotton plug, and concentrated *in vacuo*. The residue was purified by silica gel column chromatography with hexane/ethyl acetate (95/5 to 7/3) to afford salvinolone (**1d**) (27.0 mg, 86% yield) as colorless solid.

(±)-Sugiol (12) from 10a

Tricyclic compound **10a** (62.3 mg, 0.20 mmol) was dissolved in dioxane (2.0 mL) and water (2.0 mL). Sodium hydrosulfite (174 mg, 1.0 mmol) and sodium hydrogen carbonate (111 mg, 1.3 mmol) were added to the solution. After stirring for 8 h at 100 °C, the reaction was quenched by water and ethyl acetate. The organic layer was separated, and the aqueous layer was extracted with ethyl acetate. The combined extract was washed with brine, dried over anhydrous sodium sulfate, filtered through a cotton plug, and concentrated *in vacuo*. The solution of the crude product in anhydrous DMF (1.0 mL) was added to the suspension of sodium hydride (53.1 mg, 2.2 mmol) and 1-dodecanethiol (525 μL, 2.2 mmol) in anhydrous DMF (1.0 mL) at room temperature. After stirring for 8 hat reflux, the reaction was quenched by saturated aqueous ammonium chloride solution and ethyl acetate at 0 °C. The organic layer was separated, and the aqueous layer was extracted with ethyl acetate. The combined extract was washed with brine, dried over anhydrous sodium sulfate, filtered through a cotton plug, and concentrated *in vacuo*. The residue was purified by silica gel column chromatography with hexane/ethyl acetate (98/2 to 7/3) to afford sugiol (**12**) (56.8 mg, 94% yield in 2steps) as colorless solid.

^{1}H NMR ($CDCl_3$): δ 7.93 (s, 1H), 7.65 (brs, 1H), 6.80 (s, 1H), 3.23 (sep, *J* = 6.9 Hz, 1H), 2.70 (dd, *J* = 18.2, 4.1 Hz, 1H), 2.61 (dd, *J* = 18.2, 13.7 Hz, 1H), 2.15 (brd, *J* = 12.5 Hz, 1H), 1.84 (dd, *J* = 13.7, 4.1 Hz, 1H), 1.74-1.71 (m, 1H), 1.63-1.59 (m, 1H), 1.52-1.45 (m, 2H), 1.25-1.22 (m, 1H), 1.24 (d, *J* = 7.1 Hz, 3H), 1.23 (d, *J* = 7.1 Hz, 3H), 1.19 (s, 3H), 0.97 (s, 3H), 0.90 (s, 3H) ppm. ^{13}C NMR ($CDCl_3$): δ 200.0, 159.7, 156.9, 133.3, 126.7, 123.8, 110.0, 49.5, 41.3, 37.9, 37.8, 36.0, 33.3, 32.5, 26.7, 23.2, 22.5, 22.3, 21.3, 18.9 ppm. The ^{1}H and ^{13}C NMR spectra were identical to those in the literature.[9]

(±)-Ferruginol (13) from 10a

Tricyclic compound **10a** (62.1 mg, 0.20 mmol) was dissolved in ethyl acetate (2.0 mL), and palladium hydroxide (10.1 mg) was added at room temperature. After stirring for 20 h under hydrogen atmosphere with a balloon, the reaction mixture was filtered through a celite pad and washed by ethyl acetate. The combined filtrate and washings were concentrated in *vacuo*, and the residue was purified by flash silica gel column chromatography with hexane/ethyl acetate (99/1) to afford methyl ether of ferruginol (57.6 mg, 96%). Methyl ether of ferruginol (60.2 mg, 0.20 mmol) was dissolved in dichloromethane (5.0 mL). 1.0 M boron tribromide in dichloromethane (3.0 mL, 2.0 mmol) was added dropwise over 10 min at −10 °C. After stirring for 18 h at room temperature, the reaction was quenched by water and chloroform. The organic layer was separated, and the aqueous layer was extracted with chloroform. The combined extract was washed with saturated aqueous sodium hydrogen carbonate solution and brine, dried over anhydrous sodium sulfate, filtered through a cotton plug, and concentrated *in vacuo*. The residue was purified by silica gel column chromatography with hexane/ethyl acetate (98/2 to 7/3) to afford ferruginol (**13**) (44.5 mg, 78% yield) as brown solid.

^{1}H NMR ($CDCl_3$): δ 6.82 (s, 1H), 6.62 (s, 1H), 4.48 (s, 1H), 3.01 (sep, J = 6.9 Hz, 1H), 2.85 (ddd, J = 16.7, 5.5, 1.3 Hz, 1H), 2.80-2.73 (m, 1H), 2.17-2.14 (m, 1H), 1.87-1.82 (m, 1H), 1.74-1.56 (m, 3H), 1.48-1.44 (m, 1H), 1.37 (ddd, J = 13.0, 13.0, 3.6 Hz, 1H), 1.30 (dd, J = 12.4, 2.3 Hz, 1H), 1.23 (d, J = 6.9 Hz, 3H), 1.23-1.17 (m, 1H), 1.21 (d, J = 6.9 Hz, 3H), 1.16 (d, J = 0.6 Hz, 3H), 0.94 (s, 3H), 0.91 (s, 3H) ppm. ^{13}C NMR ($CDCl_3$): δ 150.7, 148.7, 131.4, 127.3, 126.6, 111.0, 50.4, 41.7, 38.9, 37.5, 33.4, 33.3, 29.8, 26.8, 24.8, 22.8, 22.6, 21.6, 19.3, 19.2 ppm. The ^{1}H and ^{13}C NMR spectra were identical to those in the literature.[9]

Supplementary References

1. Branca, S. J., Lock, R. L. & Smith III, A. B. Exploitation of the vinylogous Wolff rearrangement. An efficient total synthesis of (±)-mayurone, (±)-thujopsene, and (±)-thujopsadiene. *J. Org. Chem.* **42**, 3165–3168 (1977).
2. Seong, C., Kang, J., Chai, U., Mac, D. H. & Oh, C. H. Total Synthesis of 1-oxomiltirone and arucadiol. *Synlett* **31**, 1953-1956 (2020).
3. Hashimoto, R., Hanaya, K., Sugai, T. & Higashibayashi, S. 1,2-Rearrangement from *o*-quinols to multisubstituted catechols *via* retro Diels-Alder reaction of *o*-quinol dimers. *Bull. Chem. Soc. Jpn.* **95**, 663-672 (2022).
4. Pramanik C. *et al.* Commercial manufacturing of propofol: Simplifying the isolation process and control on related substances. *Org. Process Res. Dev.* **18**, 152-156 (2014).
5. Crossley, S. W. M., Barabé, F. & Shenvi, R. A. Simple, chemoselective, catalytic olefin isomerization. *J. Am. Chem. Soc.* **136**, 16788-216791 (2014).
6. Yang, Z. *et al.* Synthesis of variously oxidized abietane diterpenes and their antibacterial activities against MRSA and VRE. *Bioorg. Med. Chem.* **9**, 347-356 (2001).
7. Miron-Lopez, G. *et al.* Cytotoxic diterpenes from roots of *Crossopetalum gaumeri*, a Celastraceae species from Yucatan Peninsula. *Bioorg. Med. Chem. Lett.* **24**, 2105-2109 (2014).
8. Wu, J., Zhou, Y., Wang, L., Zuo, J. & Zhao, W. Terpenoids from root bark of *Celastrus orbiculatus*. *Phytochemistry* **75**, 159-168 (2012).
9. Marcos, I. S. *et al.* Lateral lithiation in terpenes: synthesis of (+)-ferruginol and (+)-sugiol. *Tetrahedron* **66**, 7773–7780 (2010).
