# Supplementary material for: Unified short syntheses of oxygenated tricyclic aromatic diterpenes by radical cyclization with a photoredox catalyst
Source: Commun Chem. 2023 Aug 21;6:169. doi: 10.1038/s42004-023-00979-2 (PMC10442340; doi:10.1038/s42004-023-00979-2)
Supplement: Supplementary file 2 — Description of Additional Supplementary Files [file 42004_2023_979_MOESM2_ESM.pdf]

Description of Additional Supplementary Files

File name: Supplementary Data 1

Description: NMR spectra

File name: Supplementary Data 2

Description: DFT calculations
