# Supplementary material for: Unified short syntheses of oxygenated tricyclic aromatic diterpenes by radical cyclization with a photoredox catalyst
Source: Commun Chem. 2023 Aug 21;6:169. doi: 10.1038/s42004-023-00979-2 (PMC10442340; doi:10.1038/s42004-023-00979-2)

Supplementary Data 1

Unified short syntheses of oxygenated tricyclic aromatic diterpenes by radical cyclization with a photoredox catalyst

Riichi Hashimoto,[1]* Kengo Hanaya,[1] Takeshi Sugai,[1] and Shuhei Higashibayashi[1]*

[1]Faculty of Pharmacy, Keio University, 1-5-30 Shibakoen, Minato-ku, Tokyo 105-8512, Japan
E-mail: riichi8222hashimoto@keio.jp, higashibayashi-sh@pha.keio.ac.jp

^{1}H and ^{13}C NMR Spectra

^{1}H and ^{13}C NMR spectra of **3**

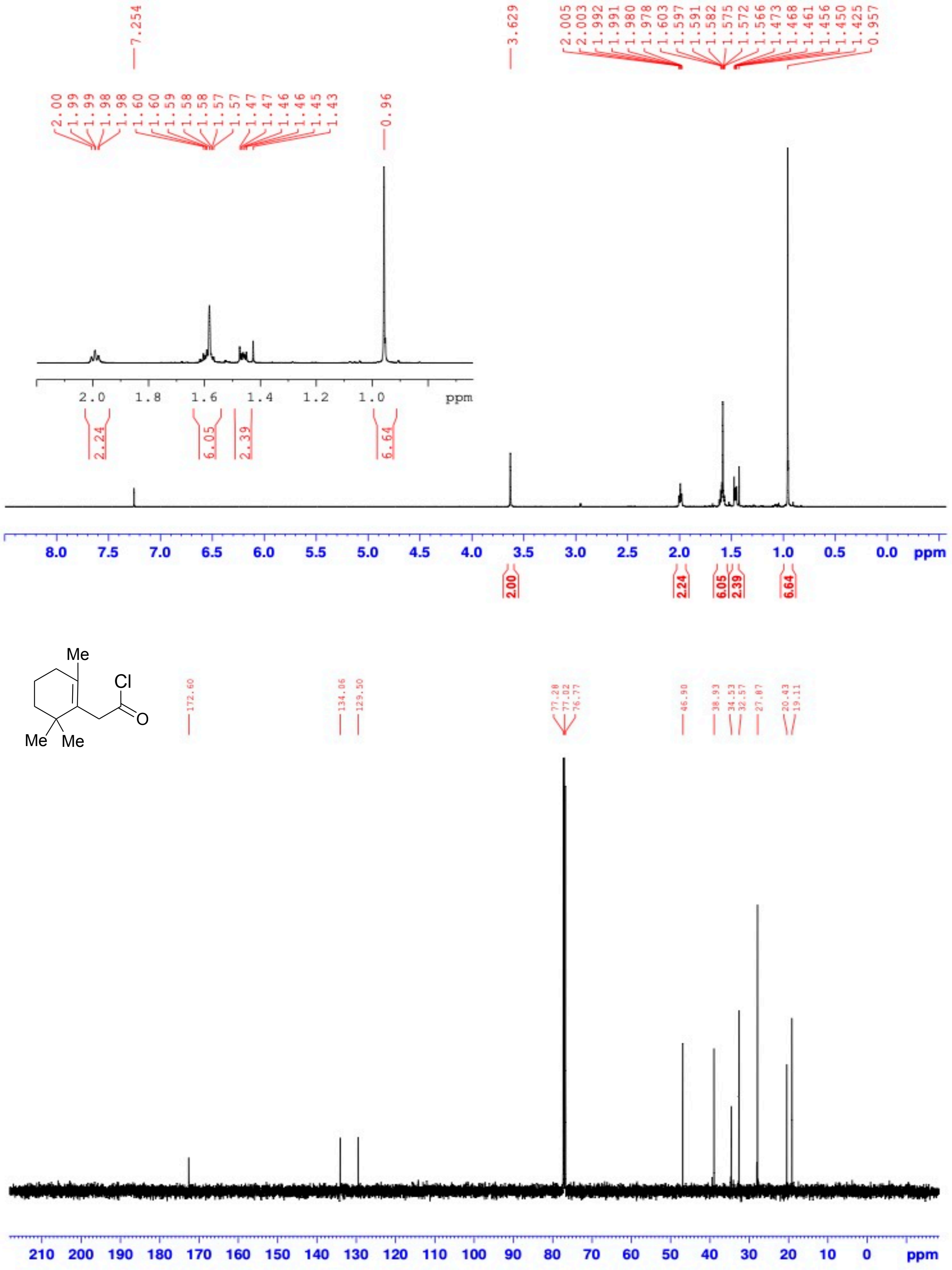

^{1}H and ^{13}C NMR spectra of **4a**

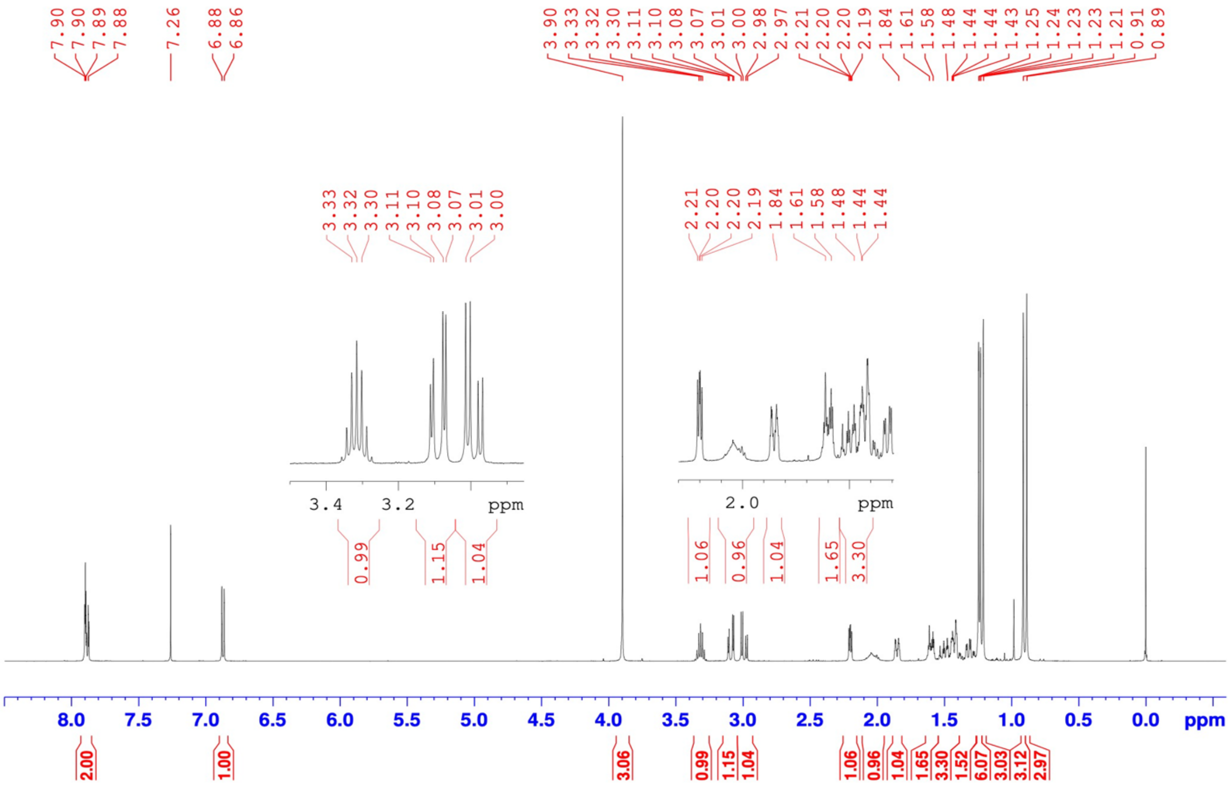

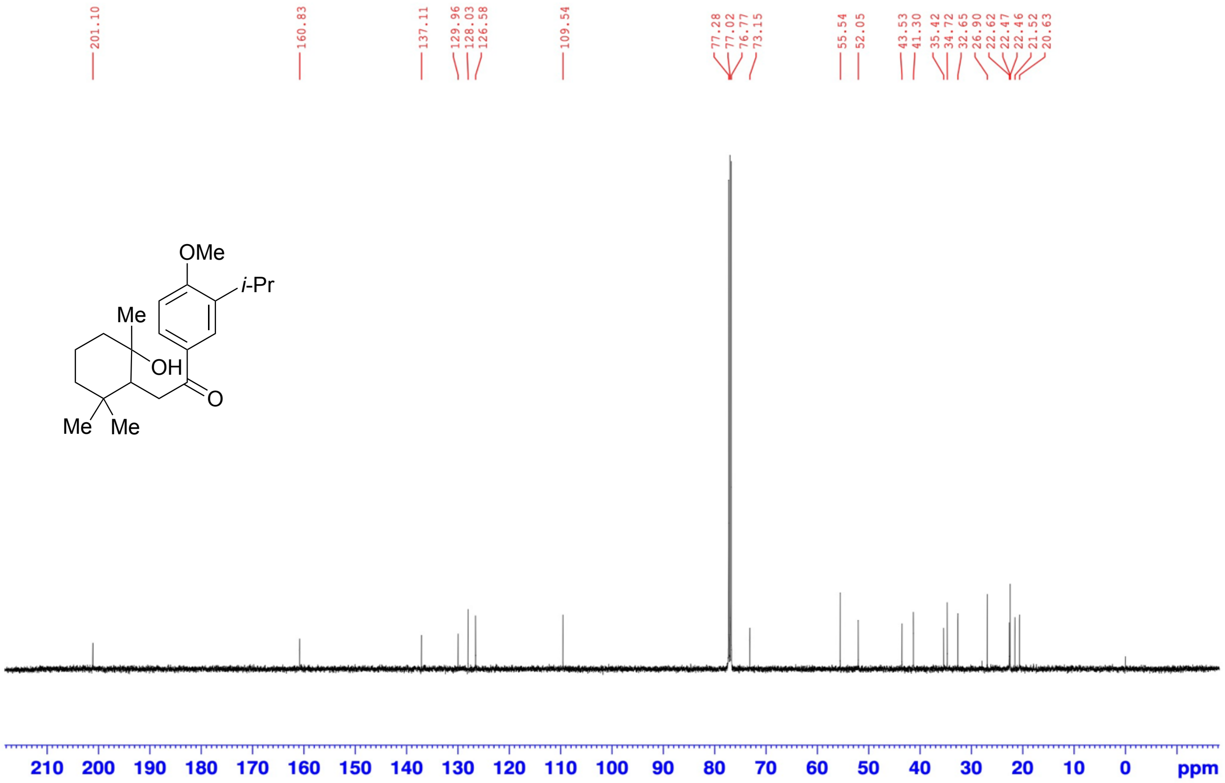

^{1}H and ^{13}C NMR spectra of **4b**

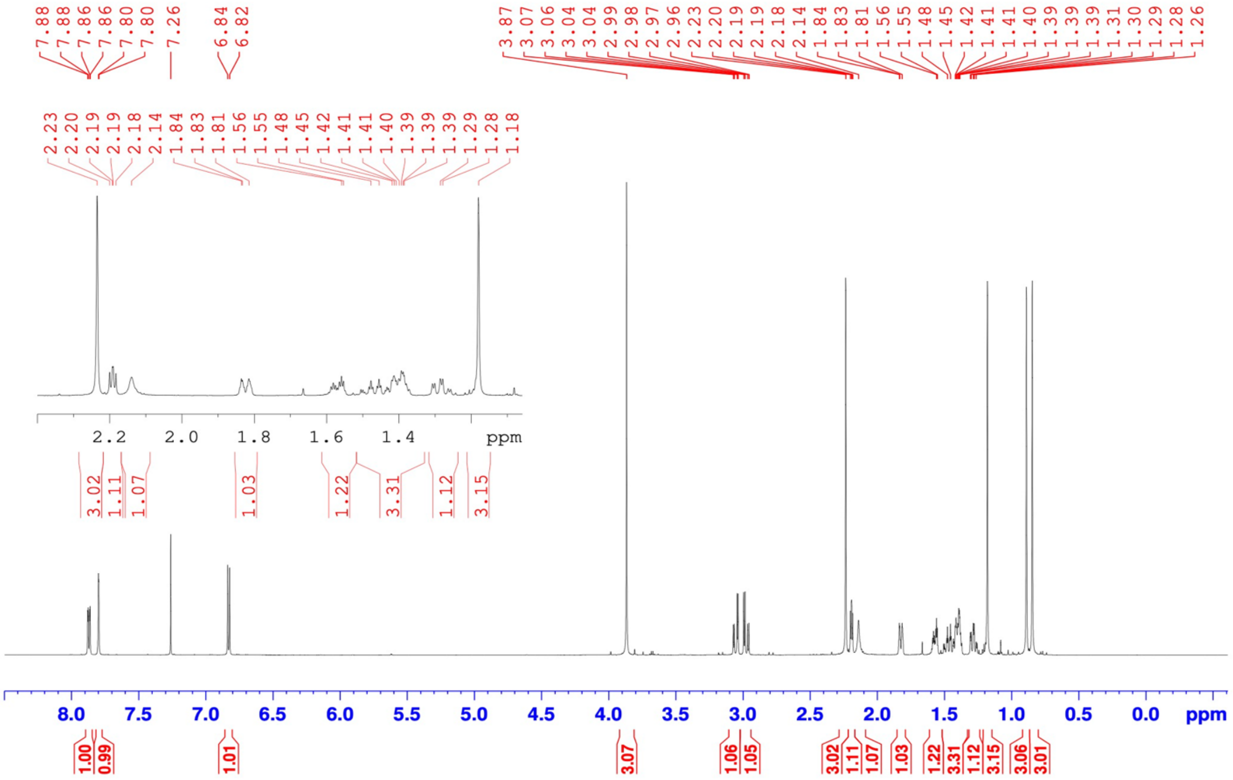

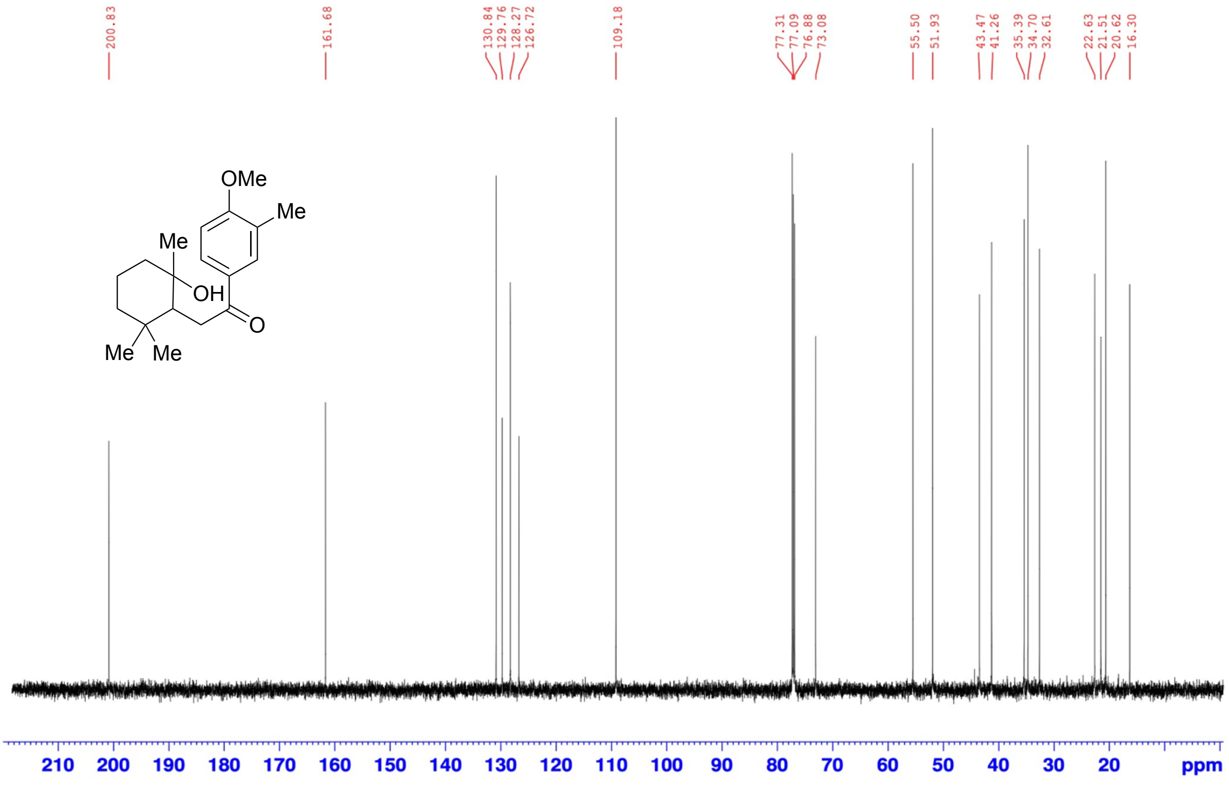

^{1}H and ^{13}C NMR spectra of **4c**

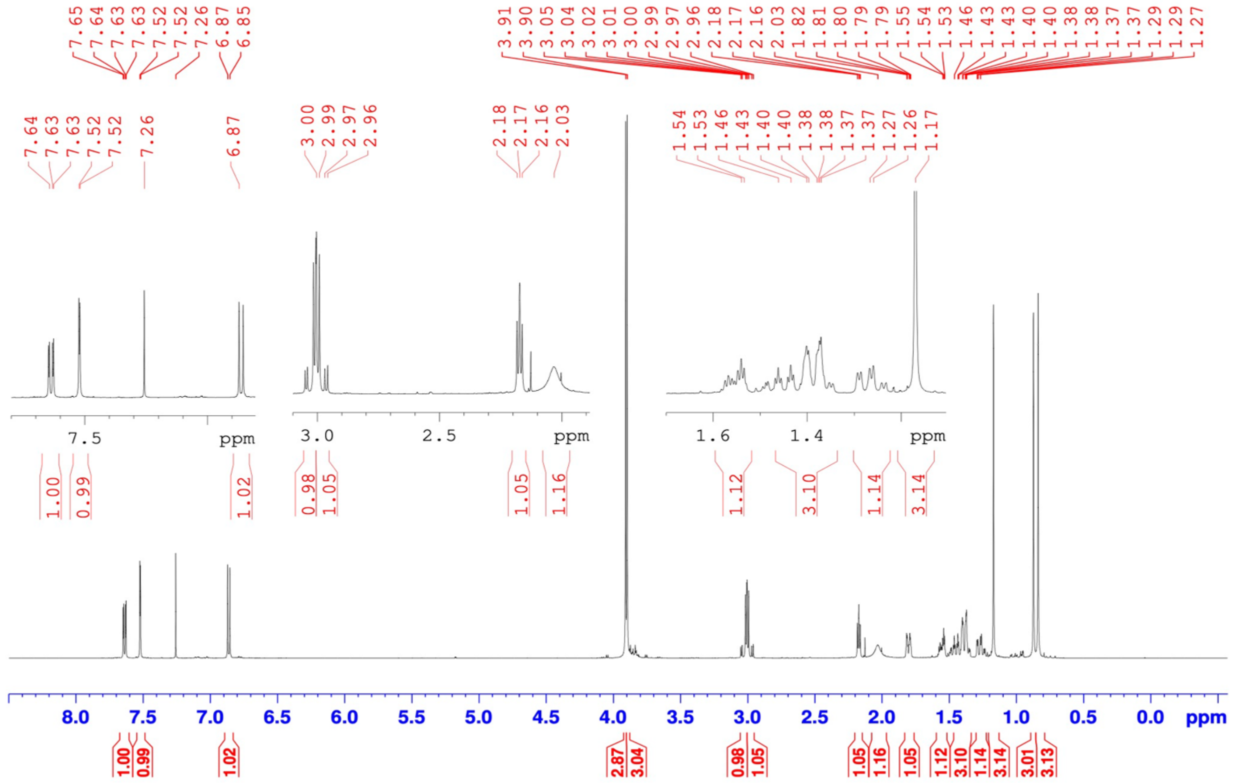

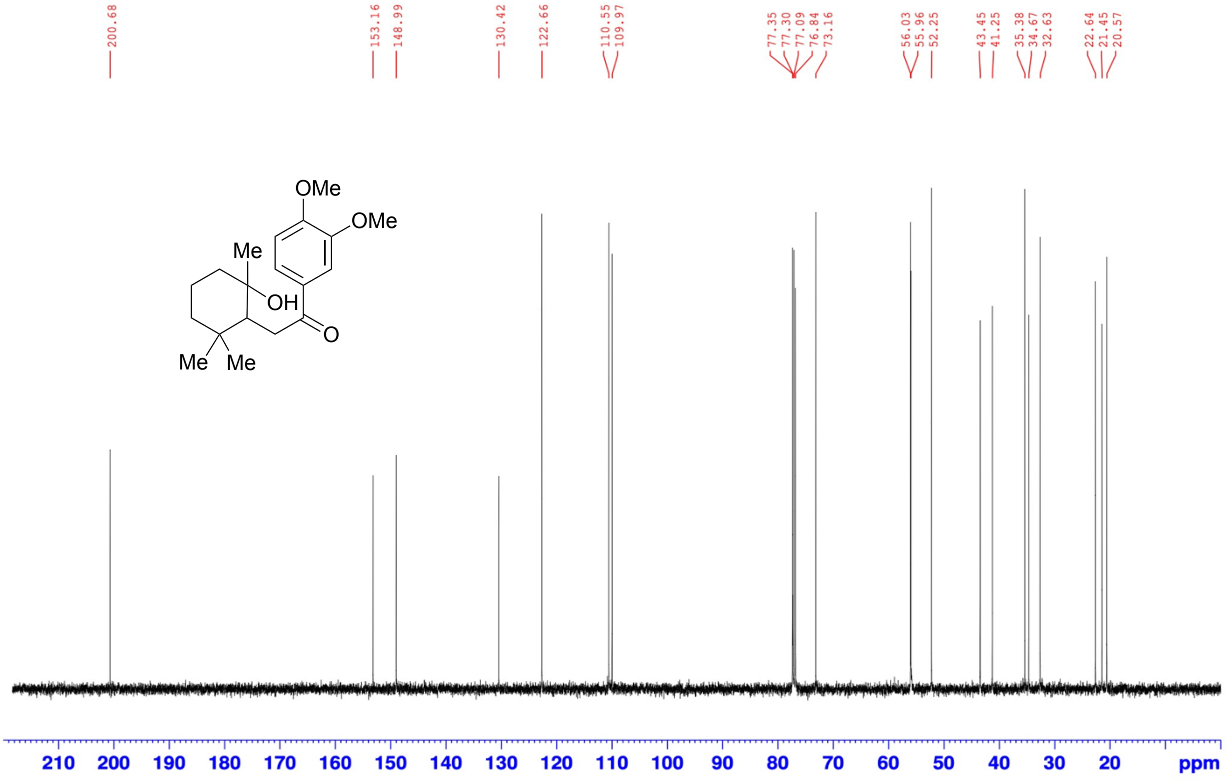

^{1}H and ^{13}C NMR spectra of **4d**

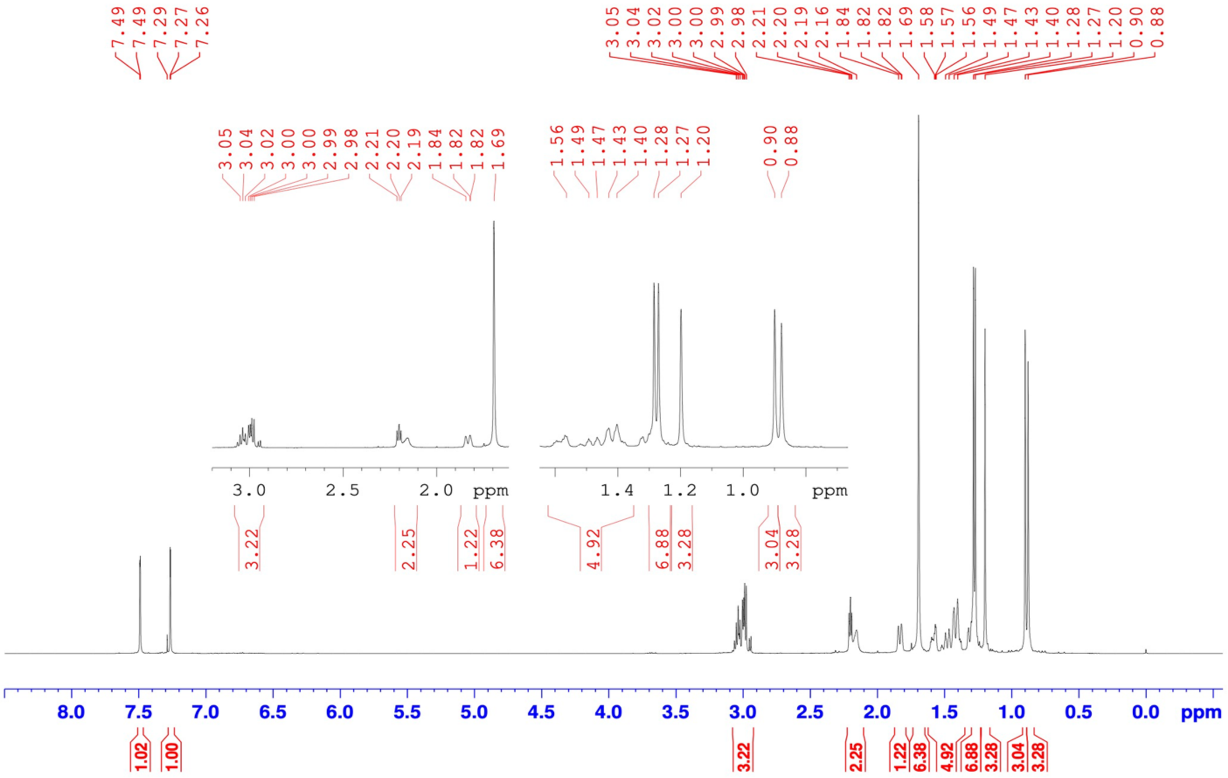

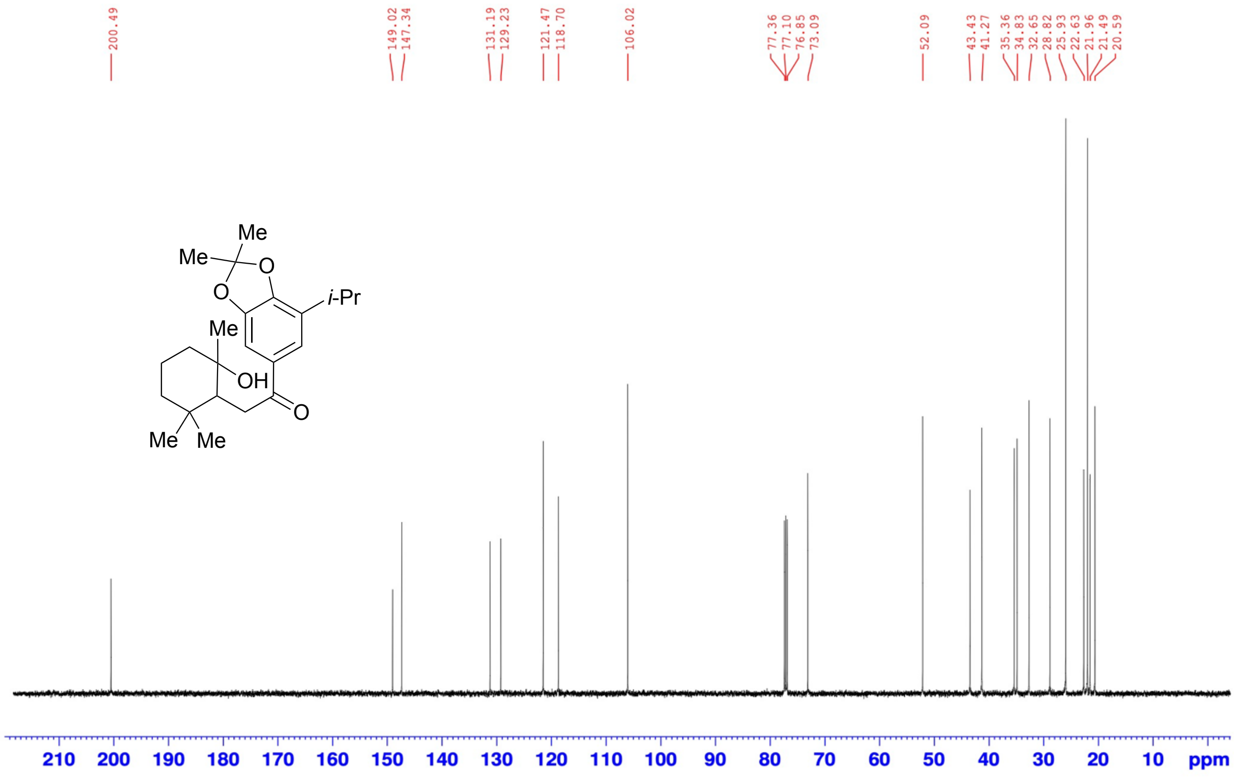

^{1}H and ^{13}C NMR spectra of **5a**

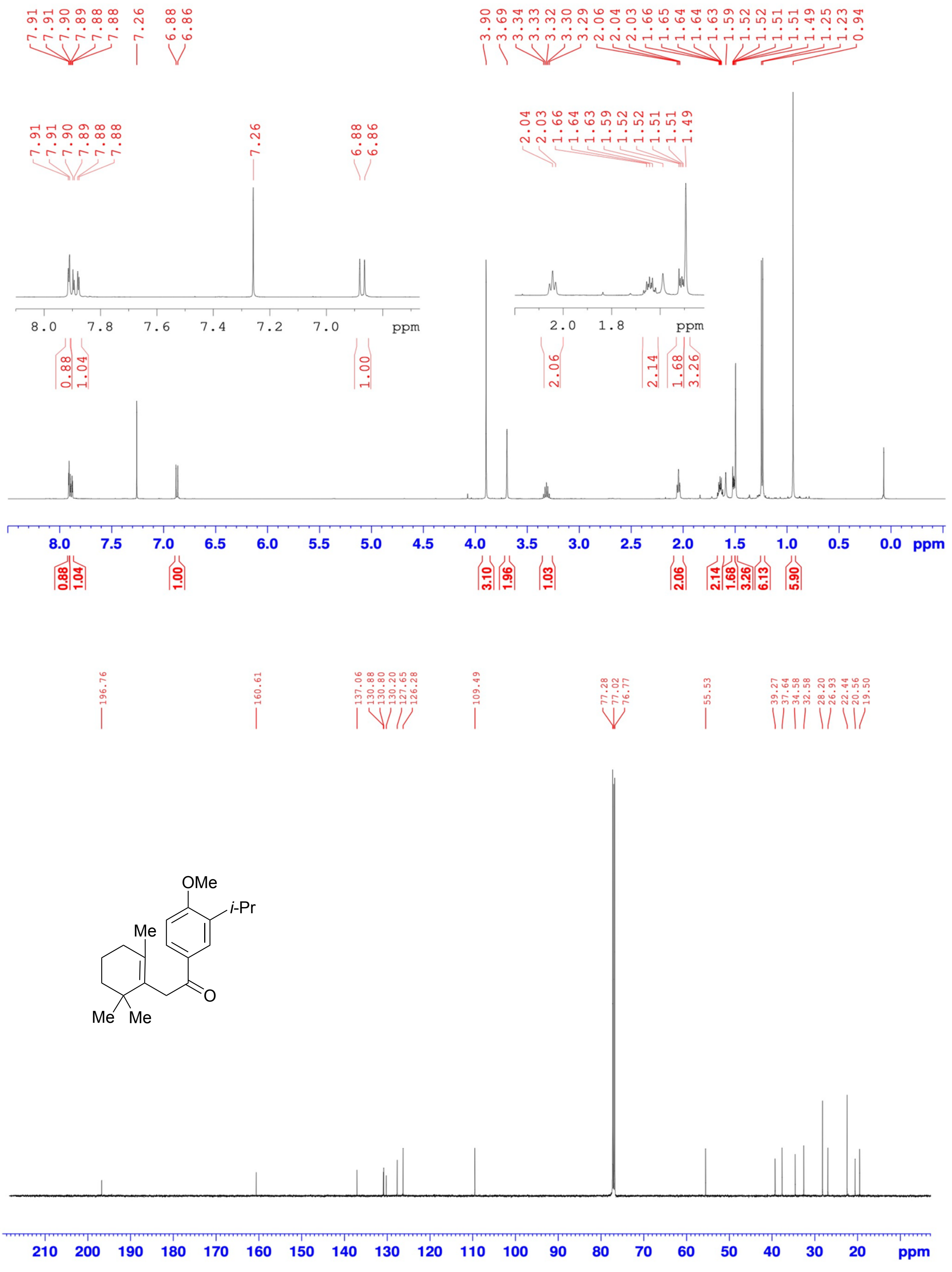

^{1}H and ^{13}C NMR spectra of **7a (Br)**

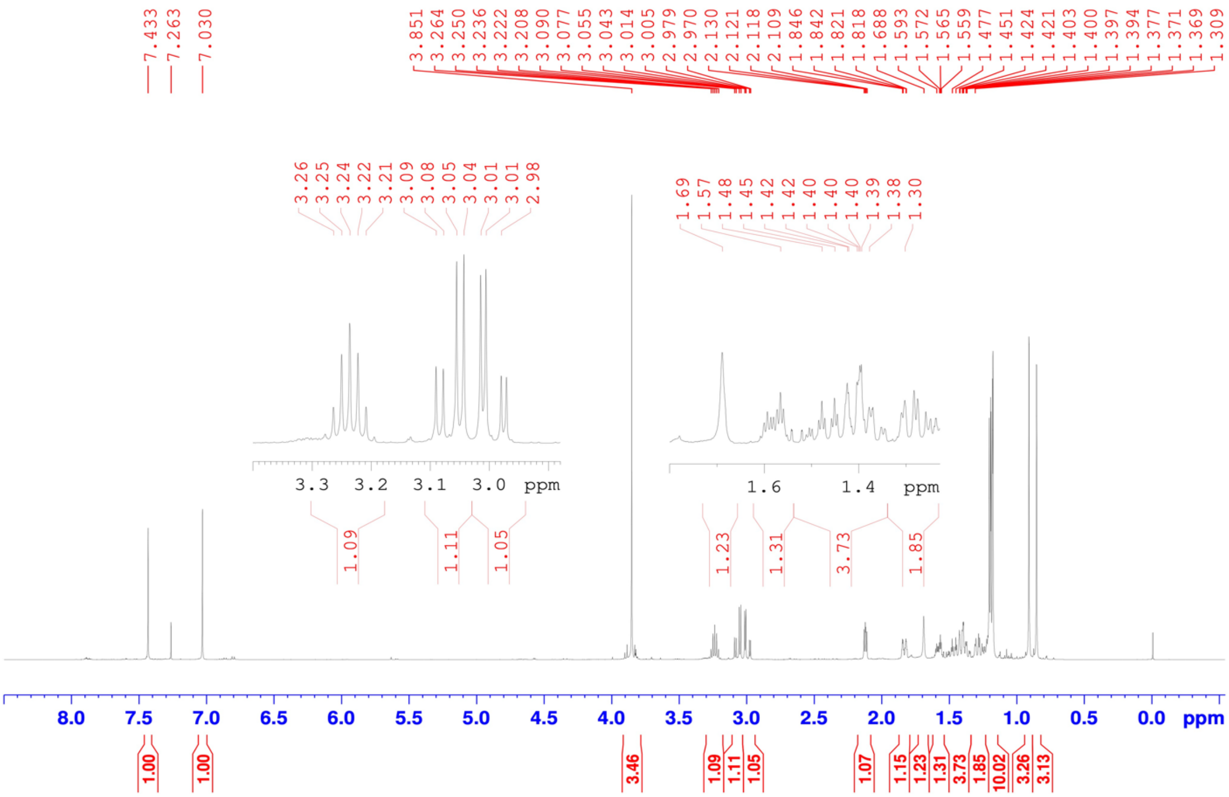

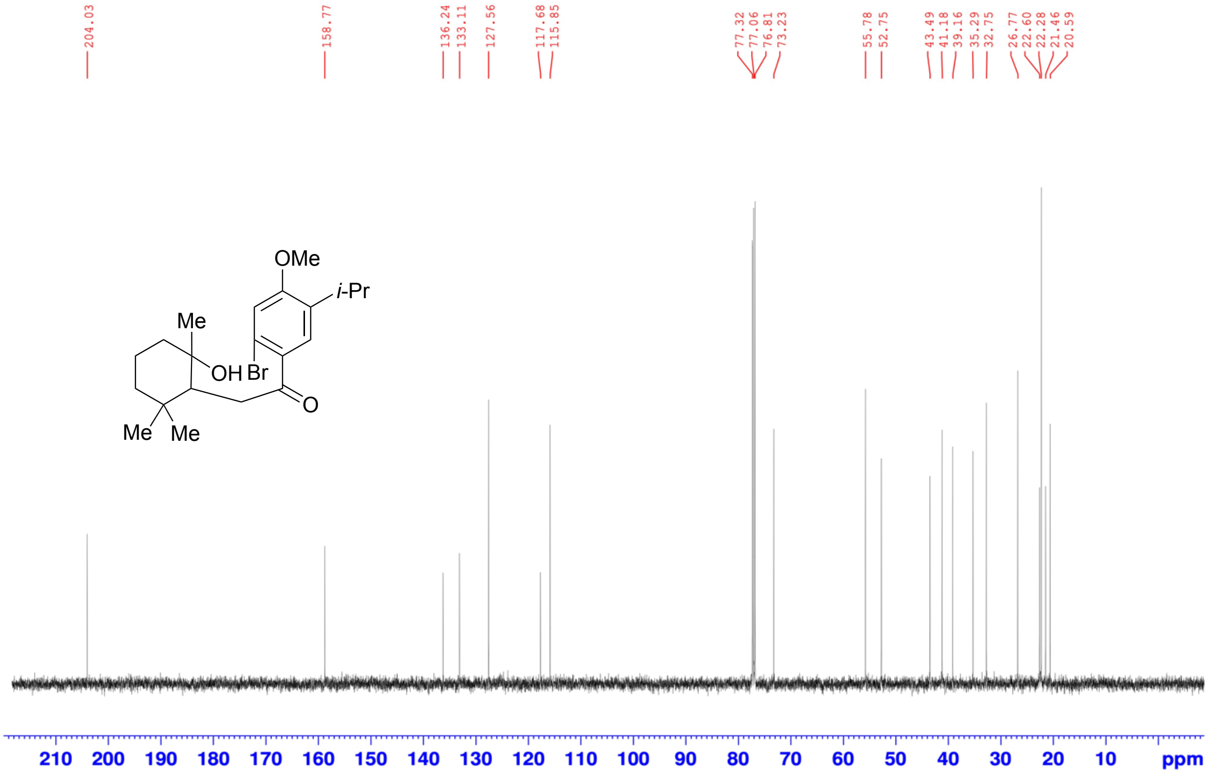

^{1}H and ^{13}C NMR spectra of **7a (I)**

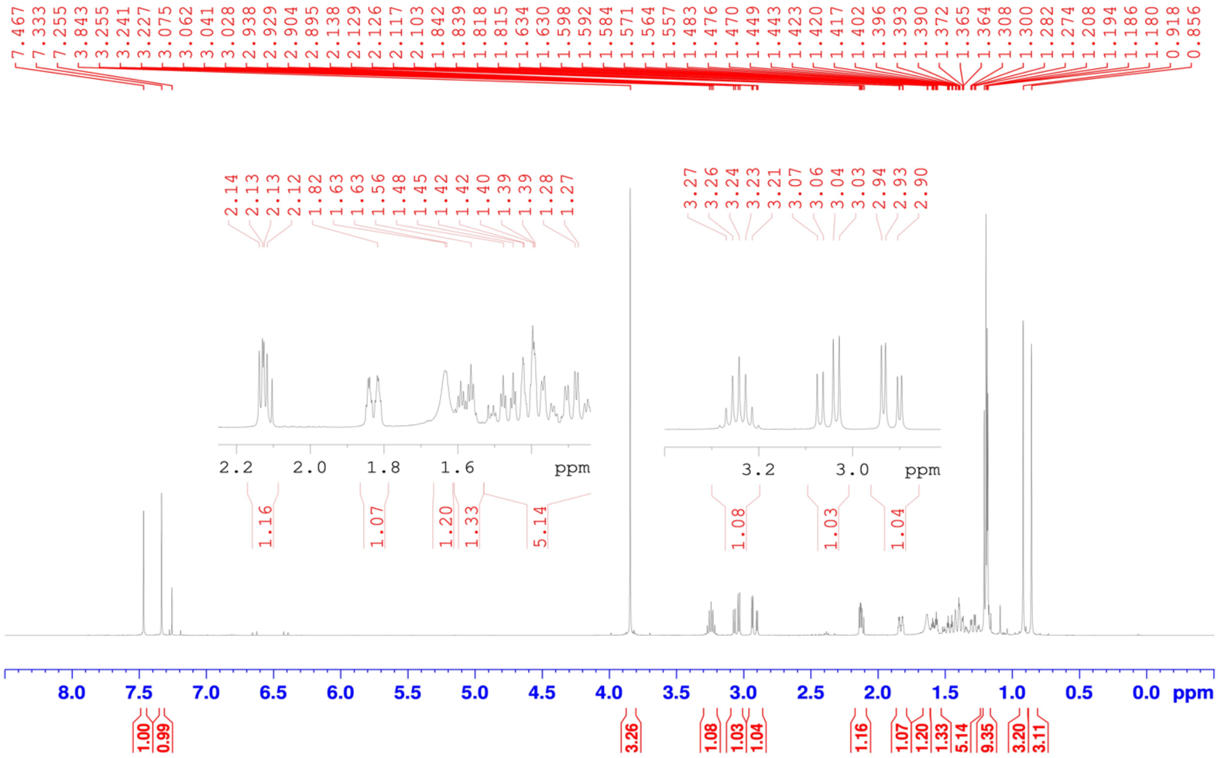

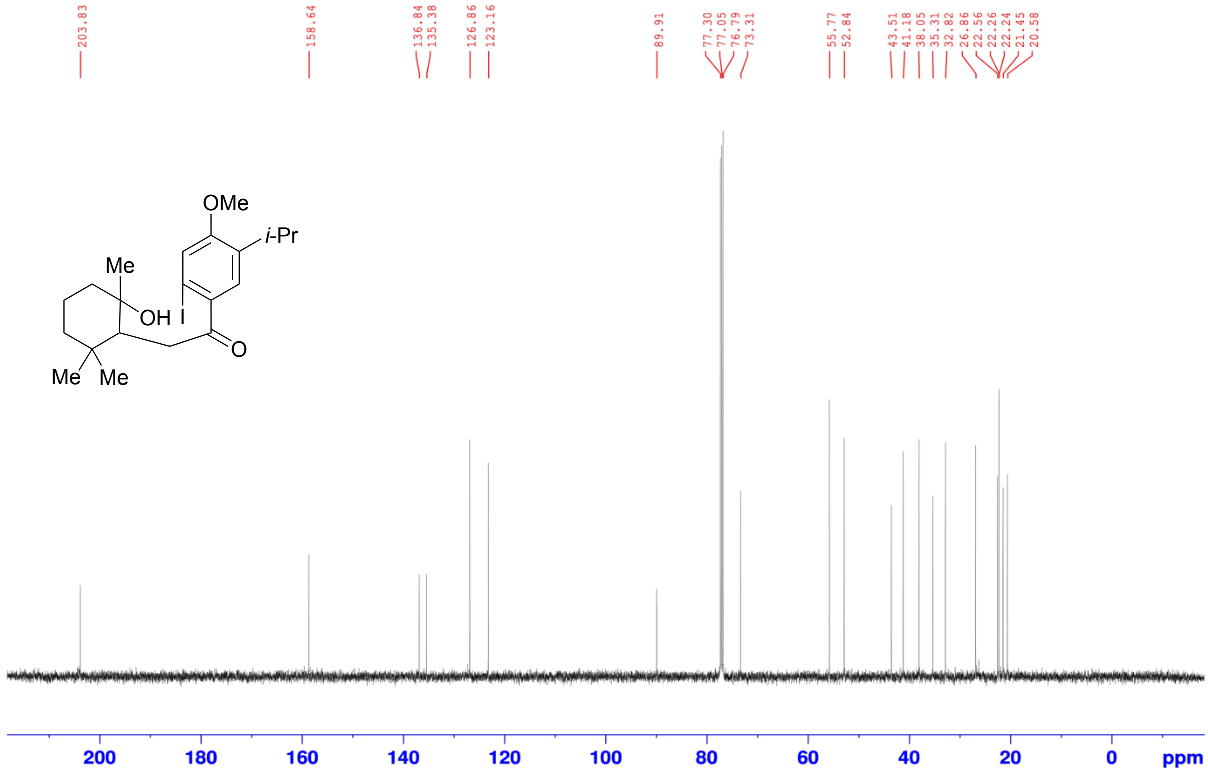

^{1}H and ^{13}C NMR spectra of **6a (Br)**

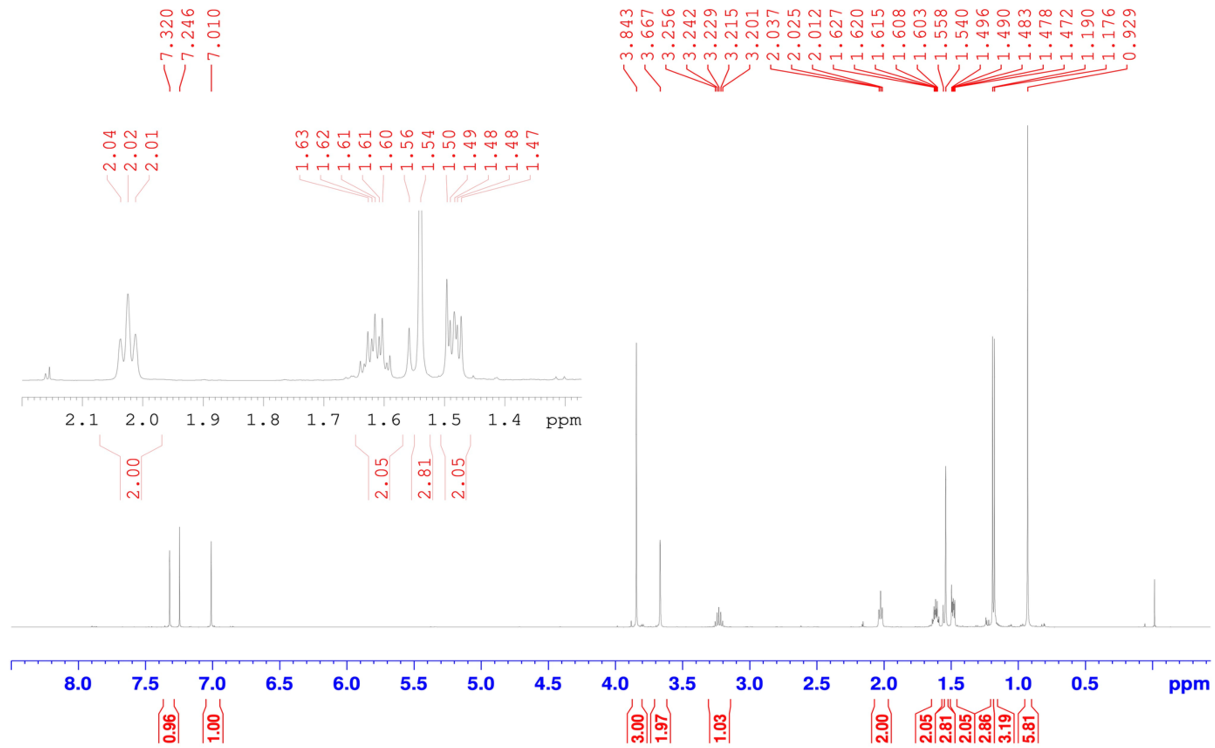

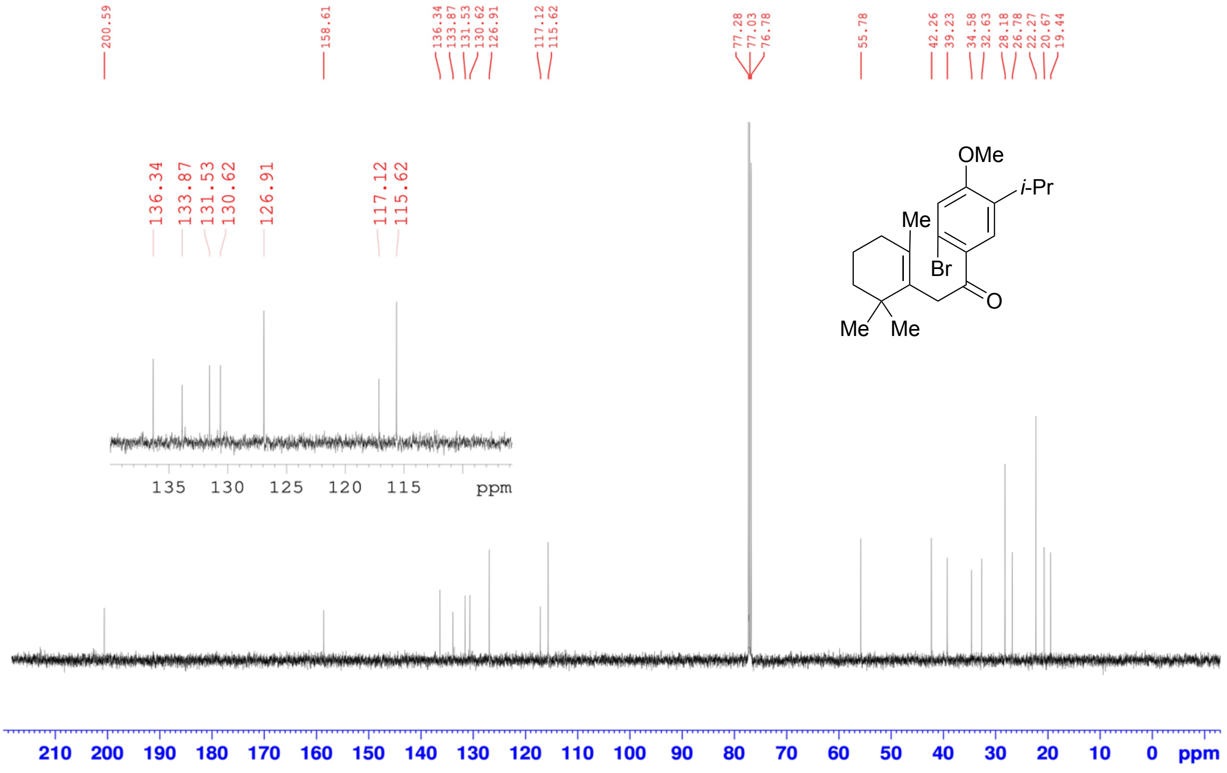

^{1}H and ^{13}C NMR spectra of **8a**

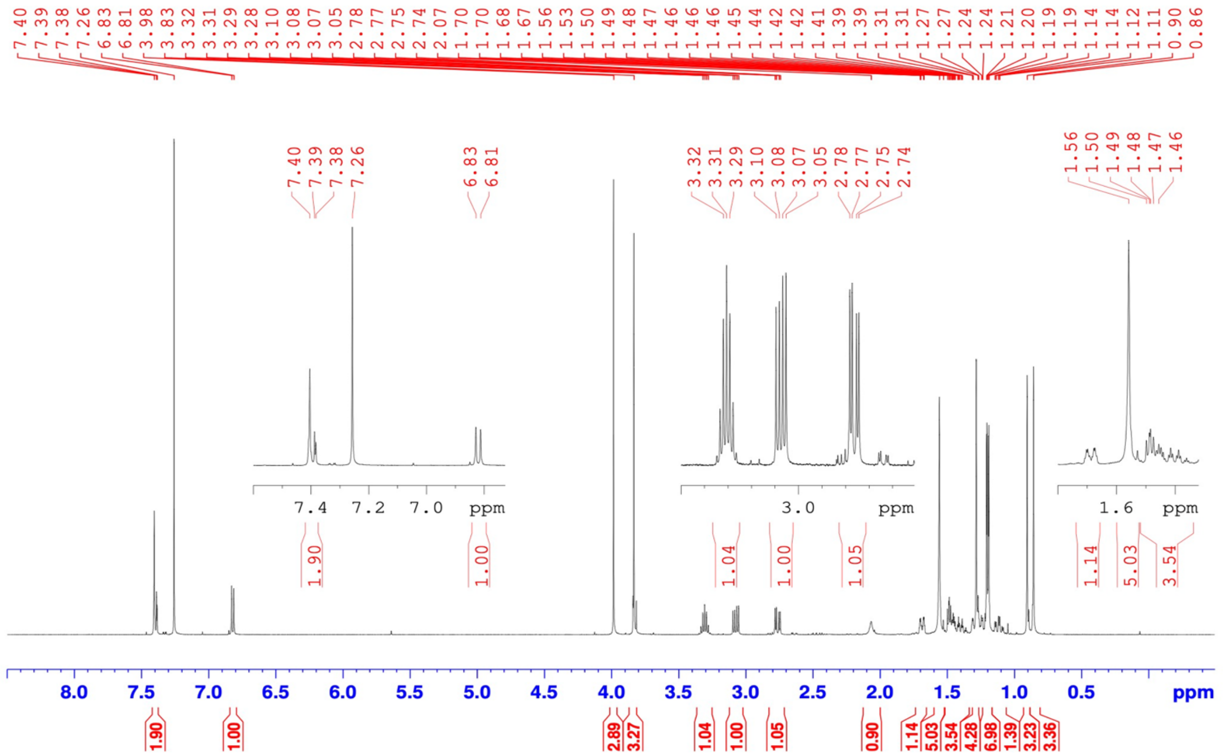

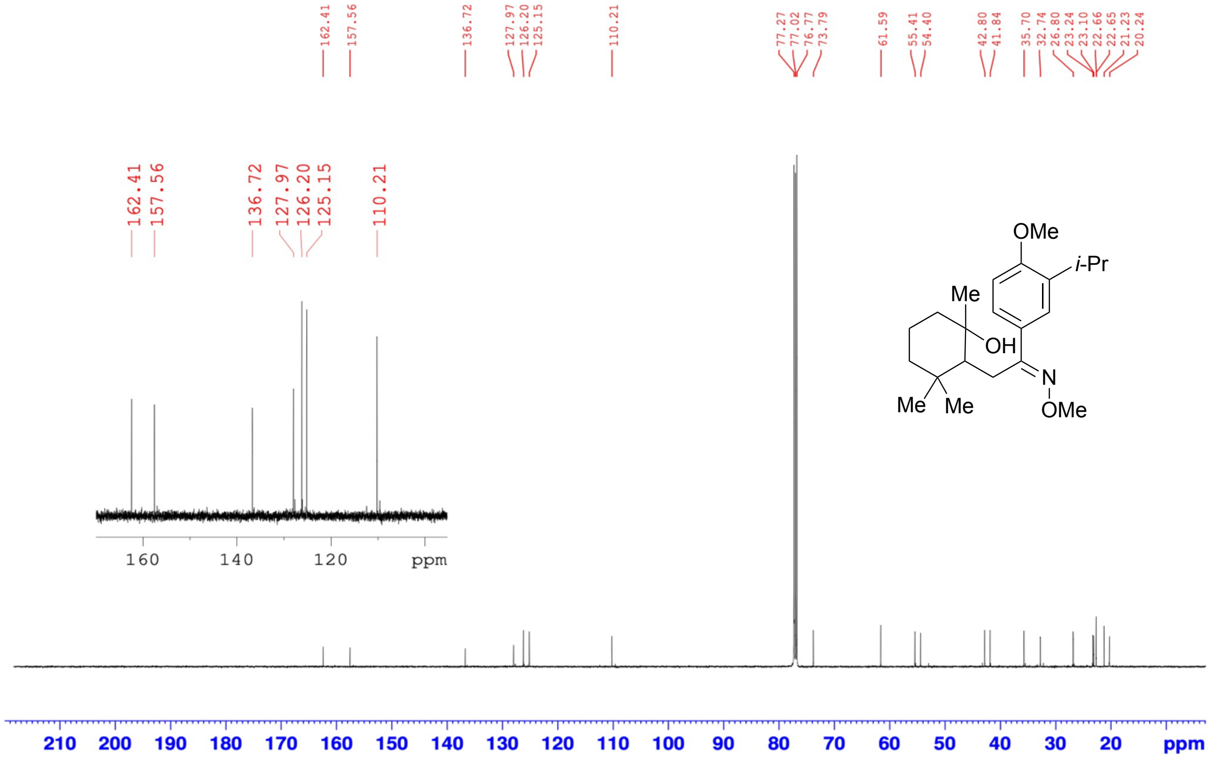

^{1}H and ^{13}C NMR spectra of **8b**

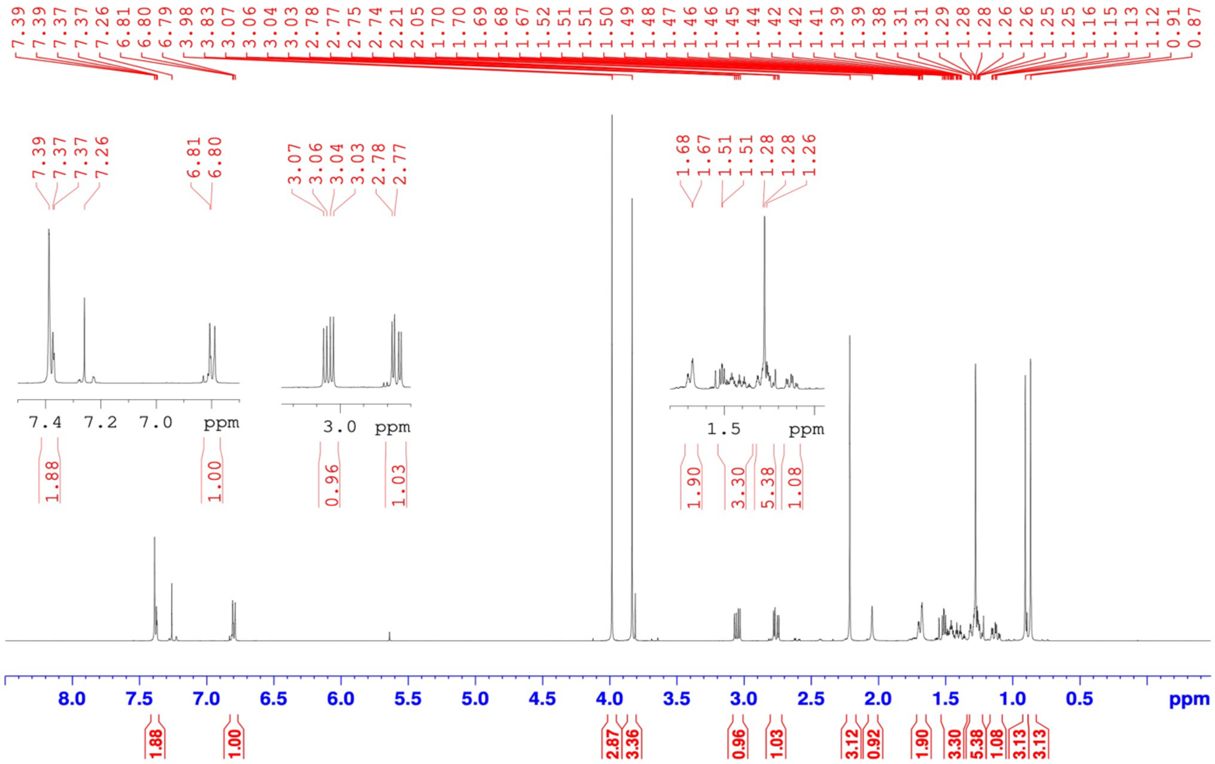

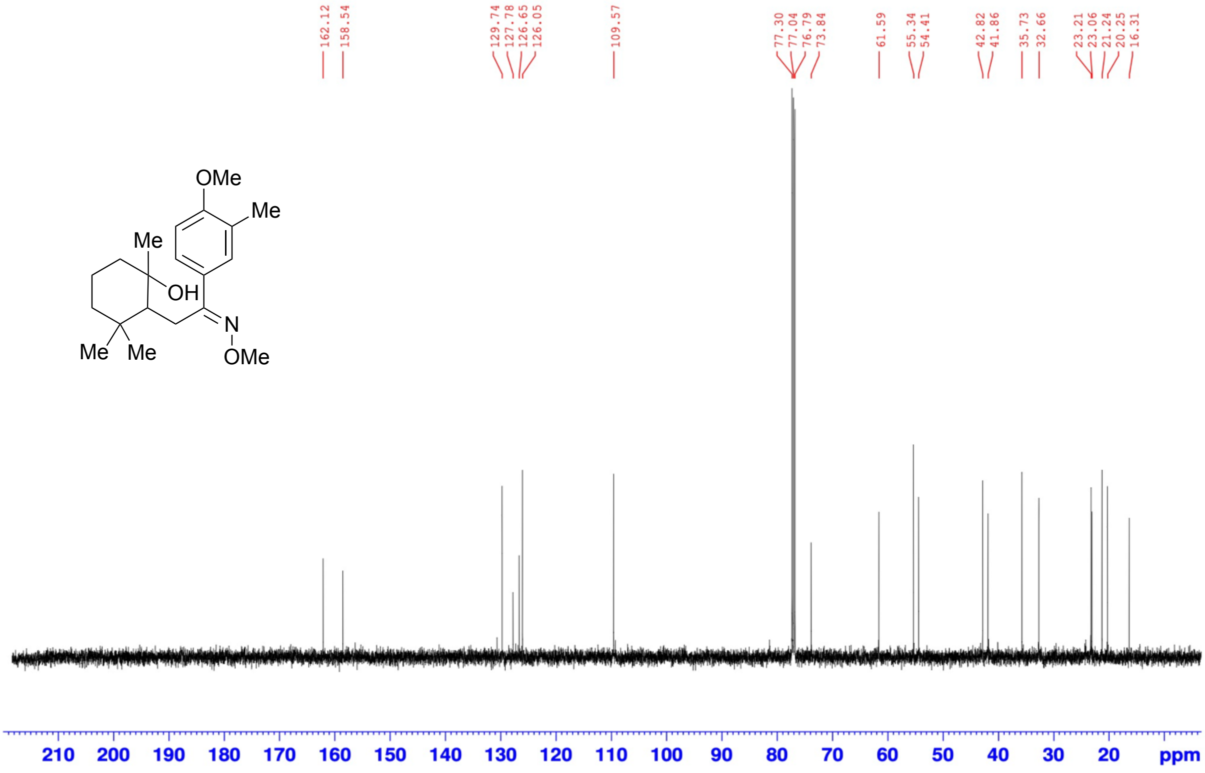

^{1}H and ^{13}C NMR spectra of **8c**

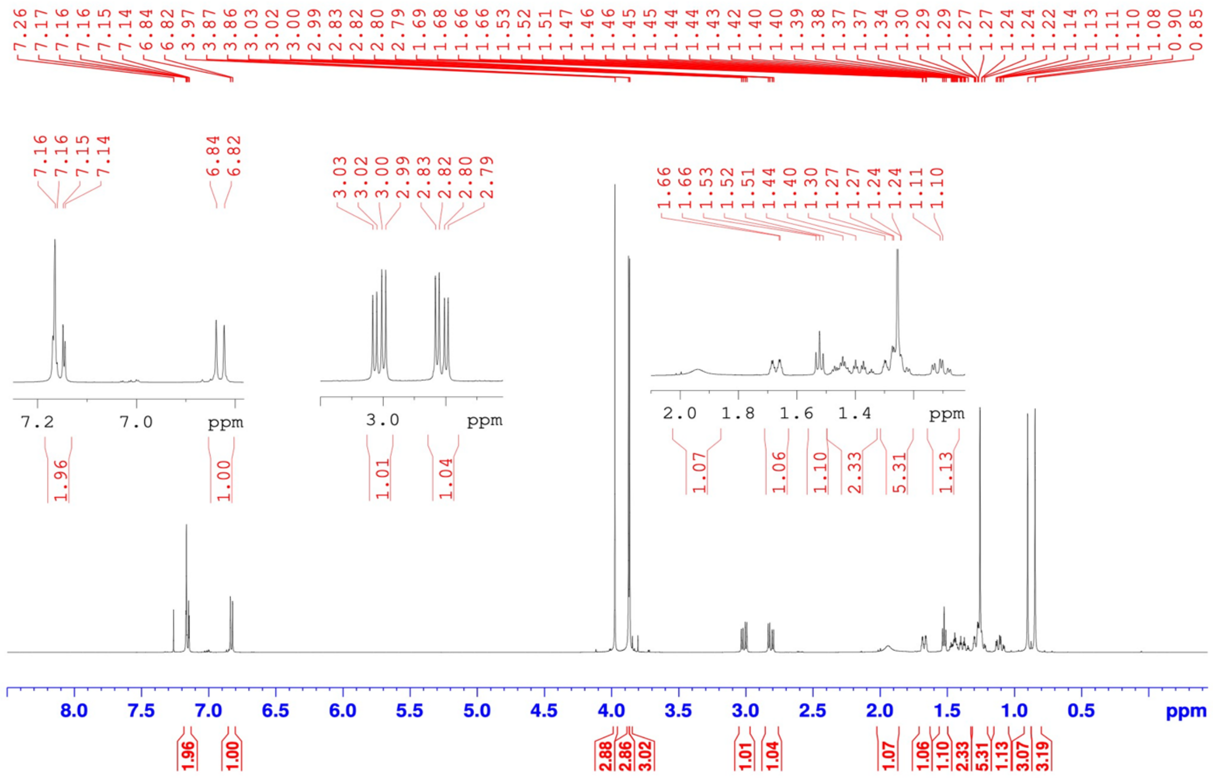

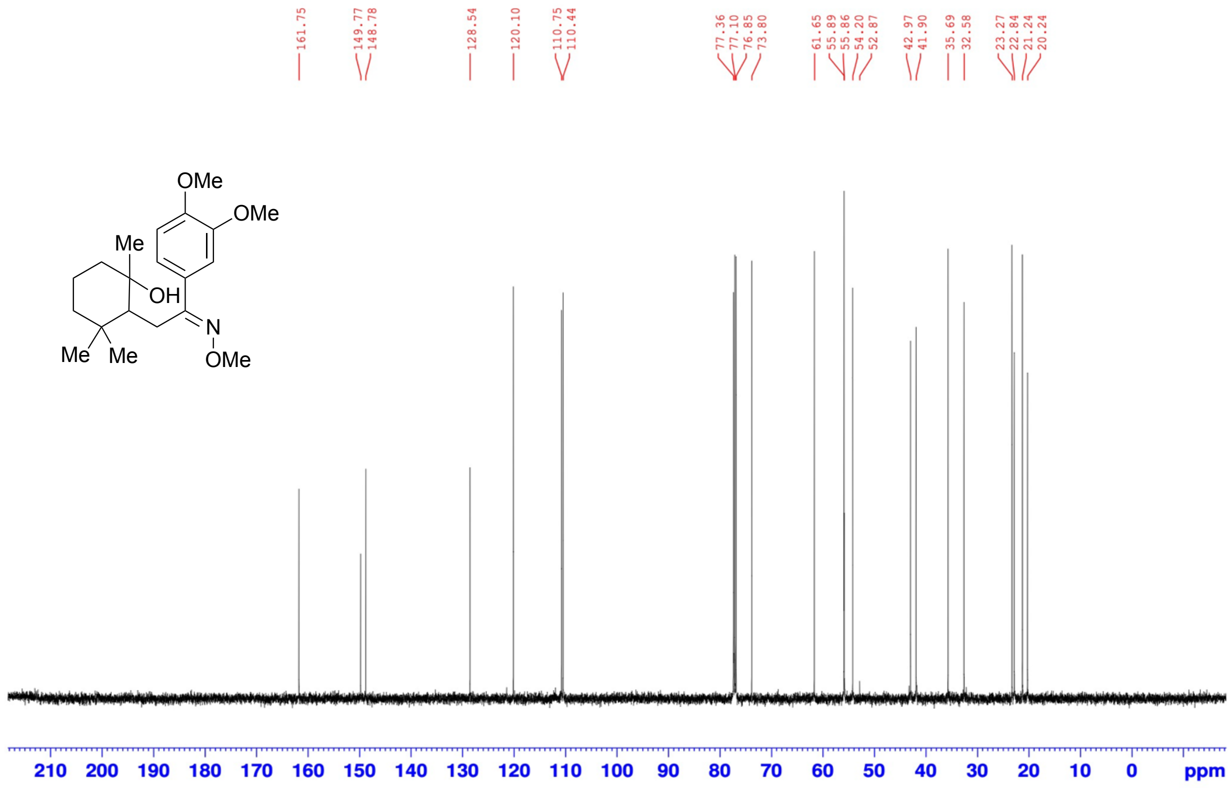

^{1}H and ^{13}C NMR spectra of **8d**

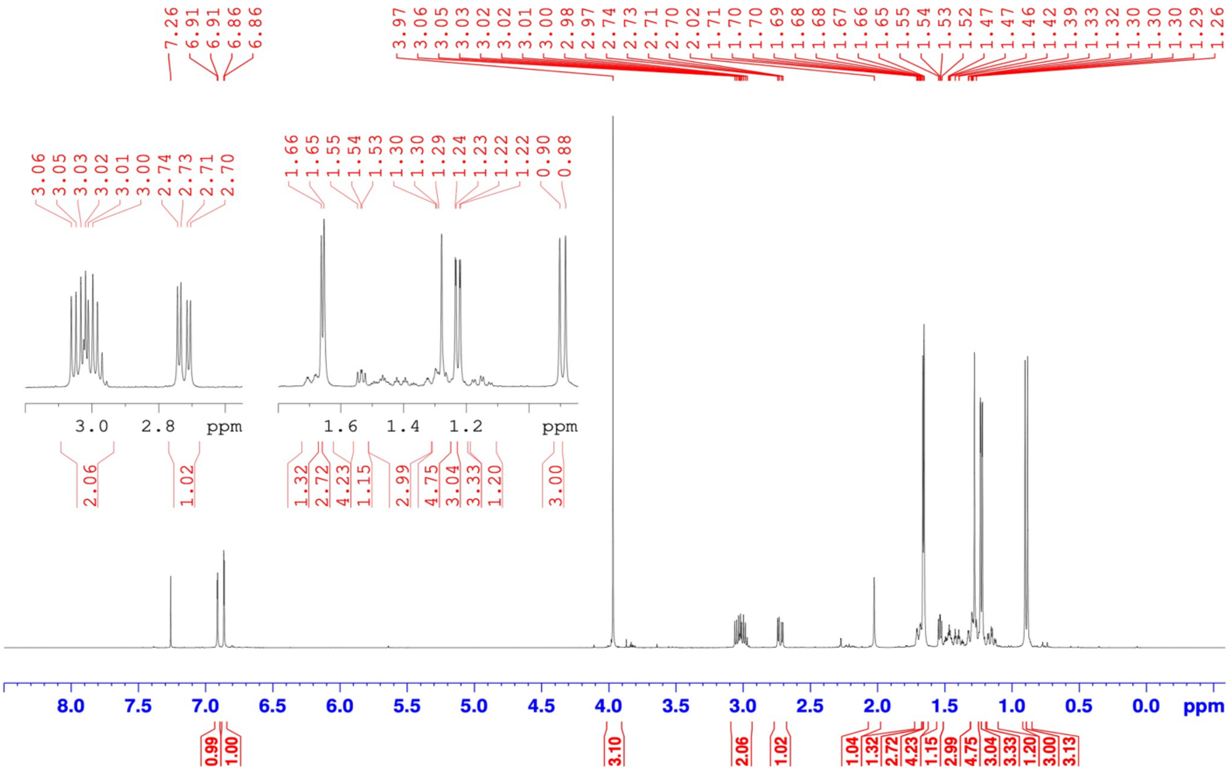

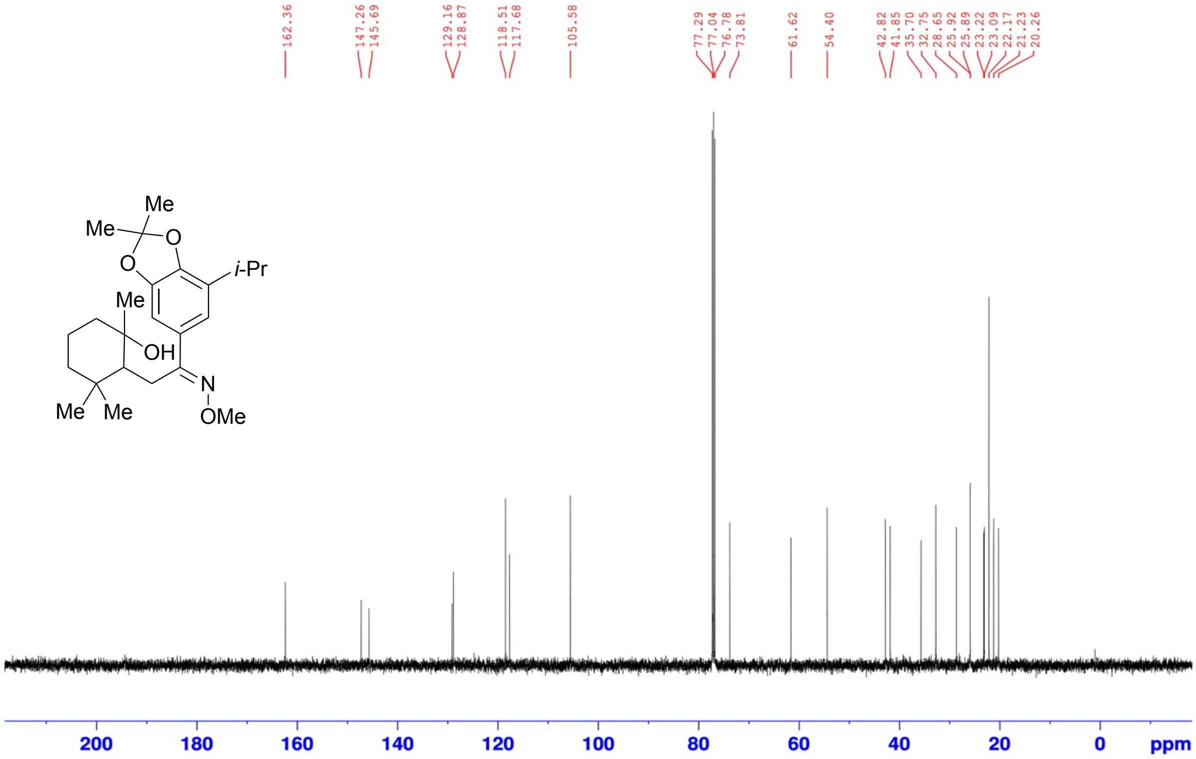

^{1}H and ^{13}C NMR spectra of **9a**

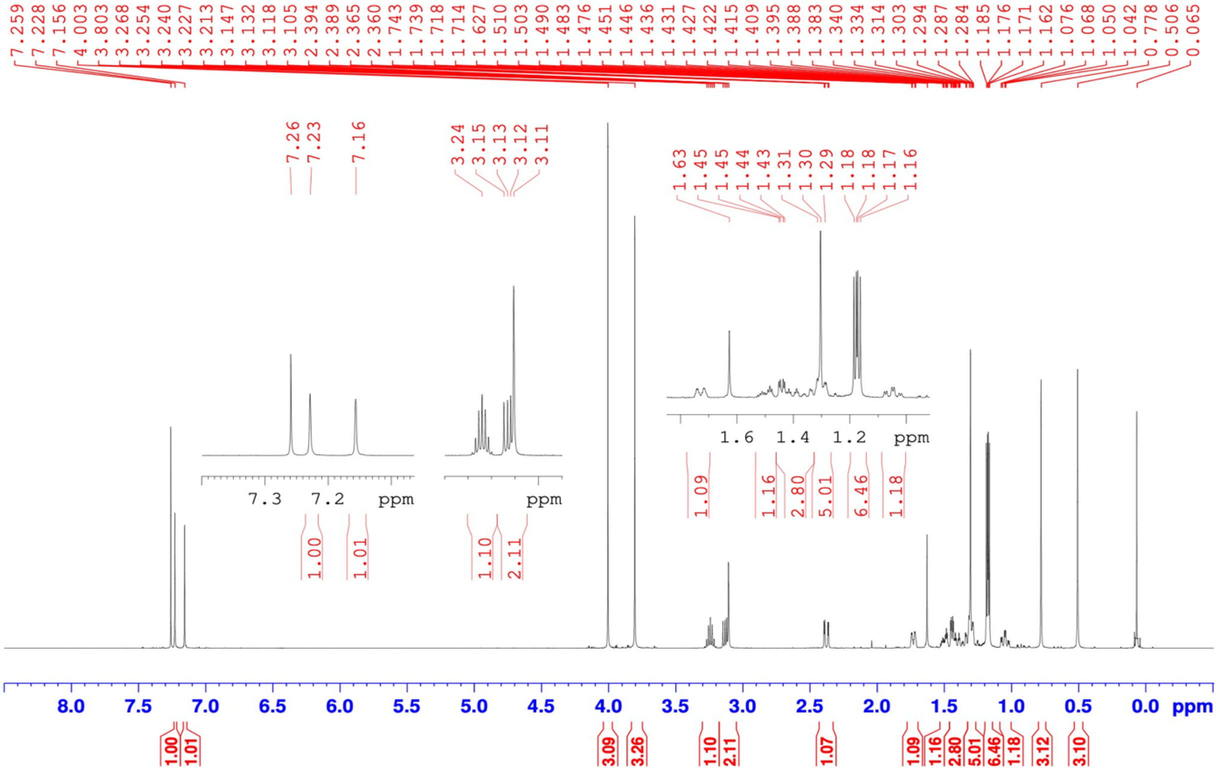

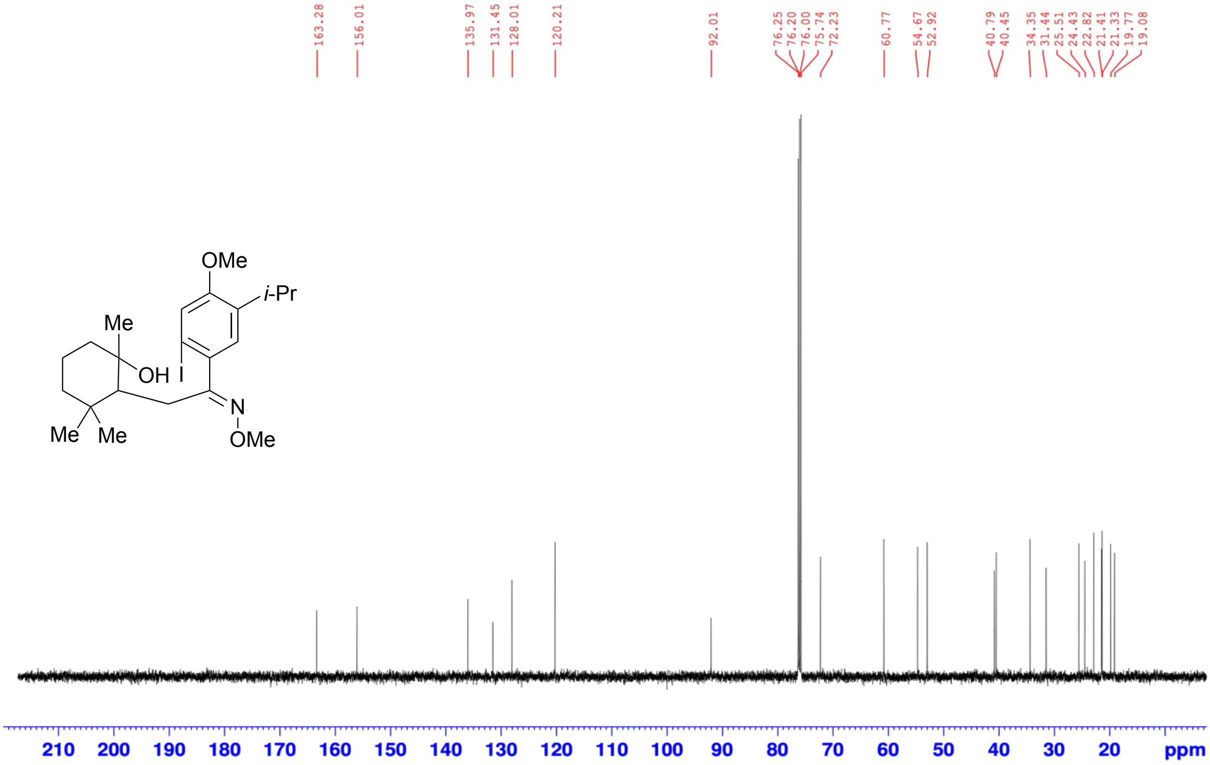

^{1}H and ^{13}C NMR spectra of **9b**

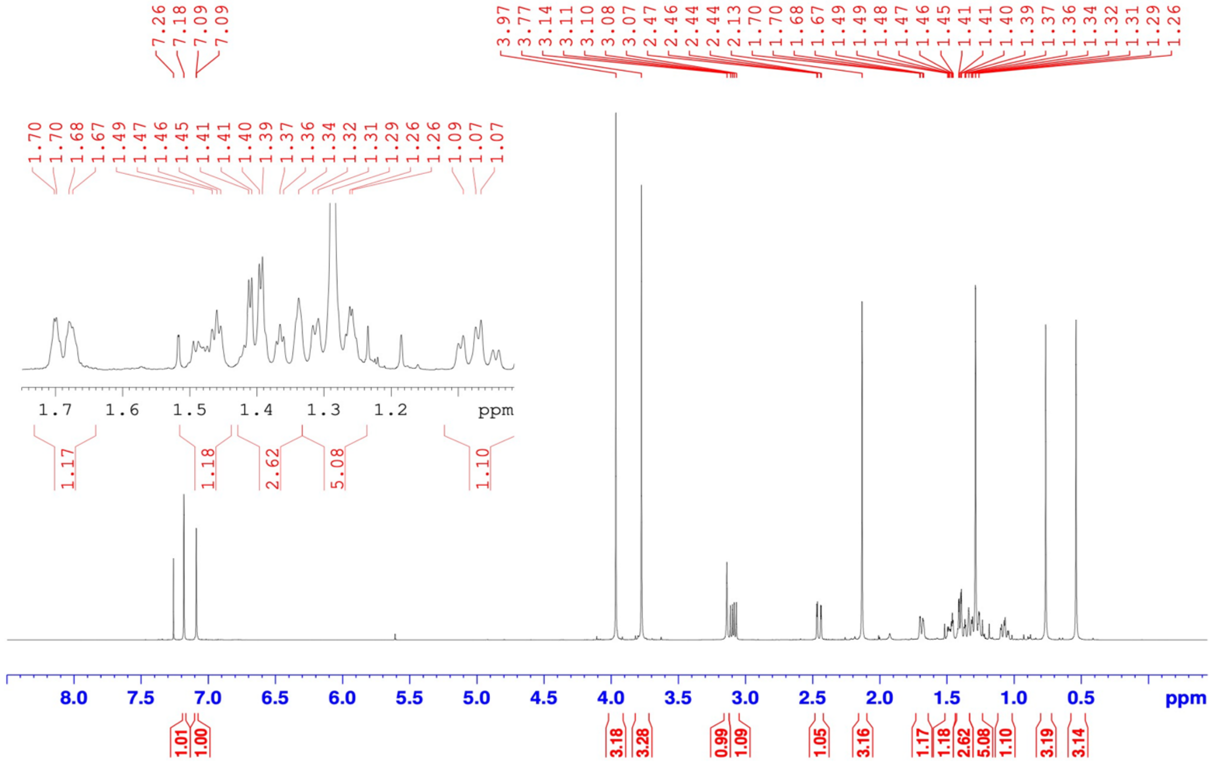

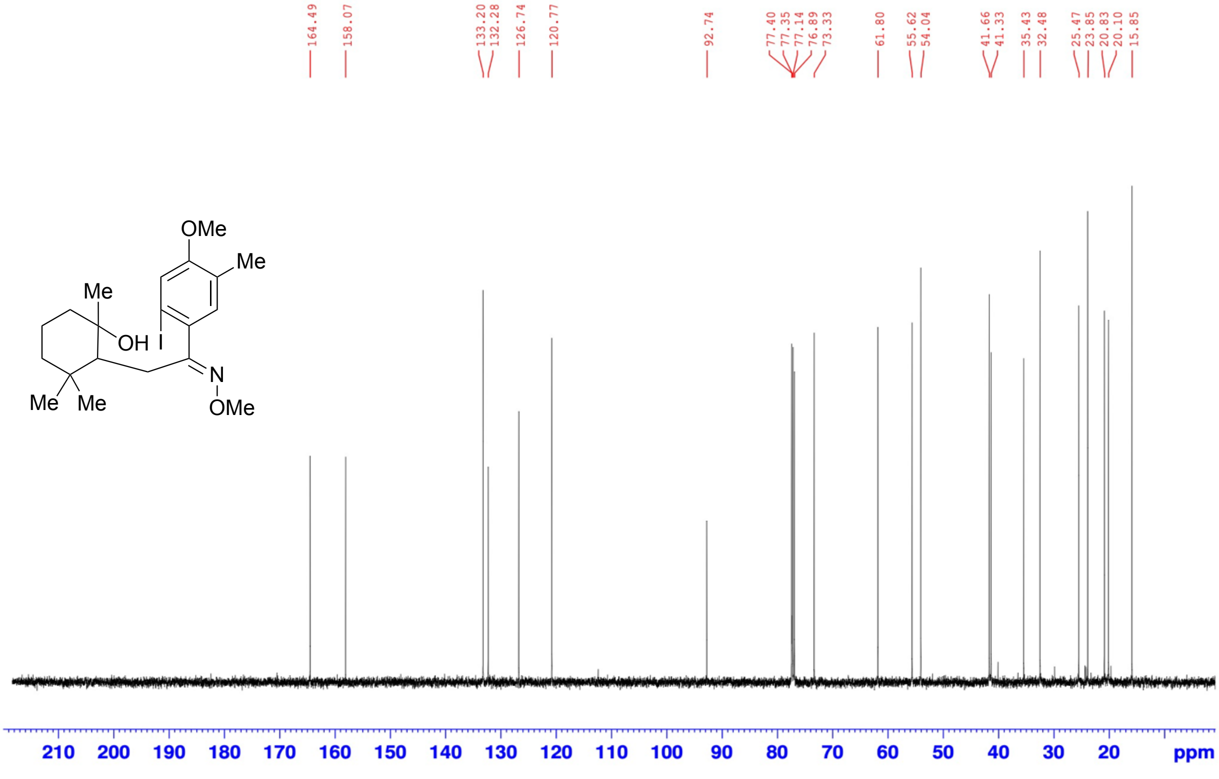

^{1}H and ^{13}C NMR spectra of **9c**

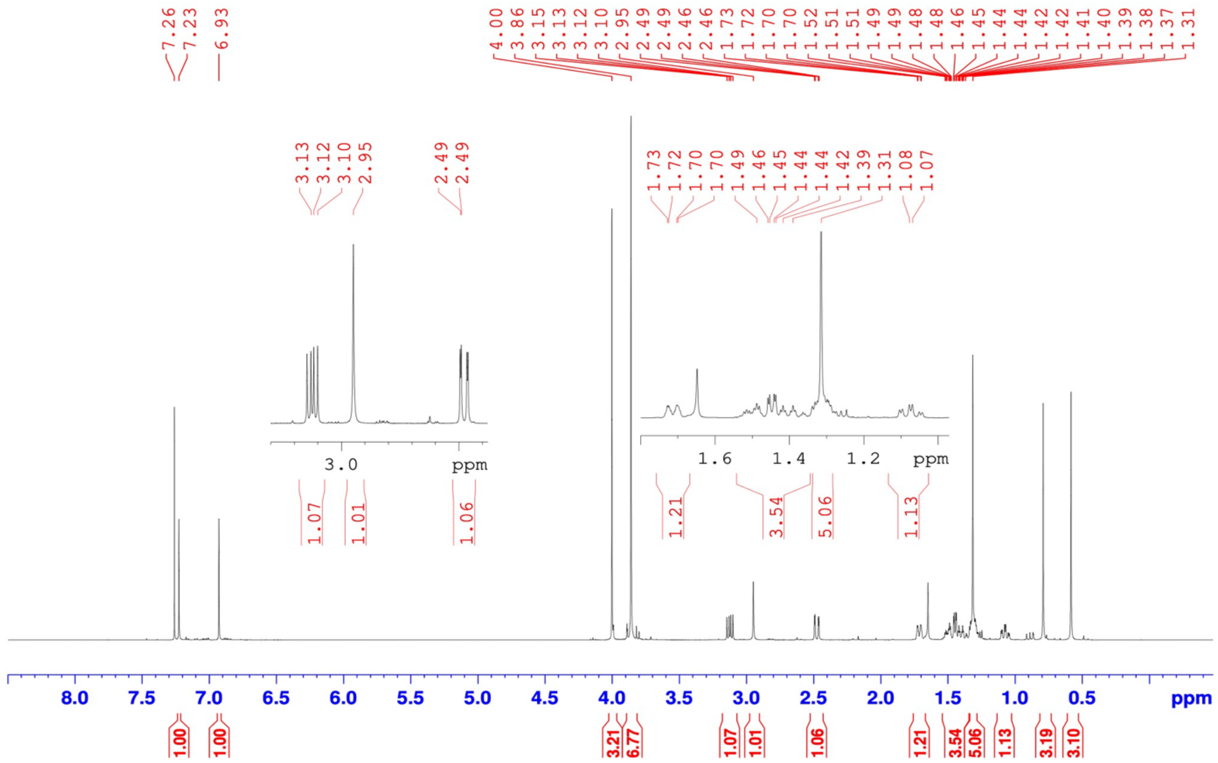

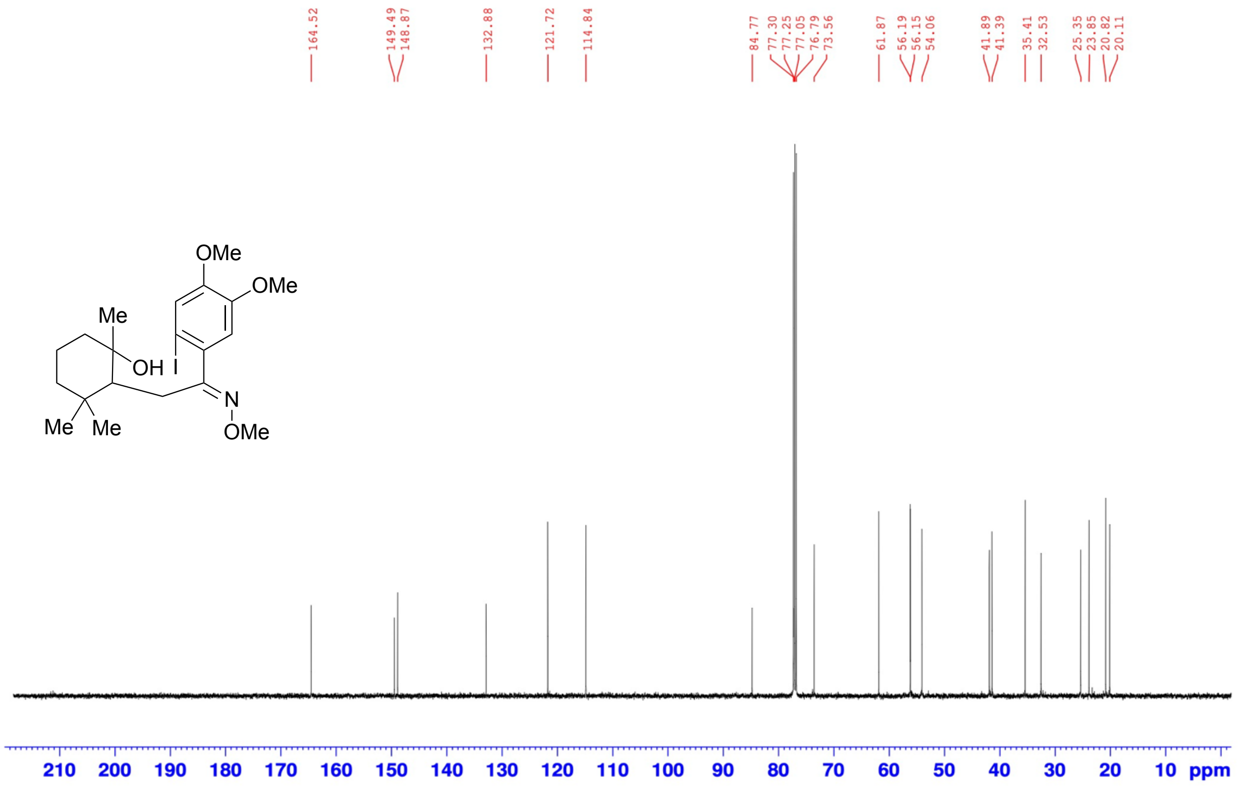

^{1}H and ^{13}C NMR spectra of **9d**

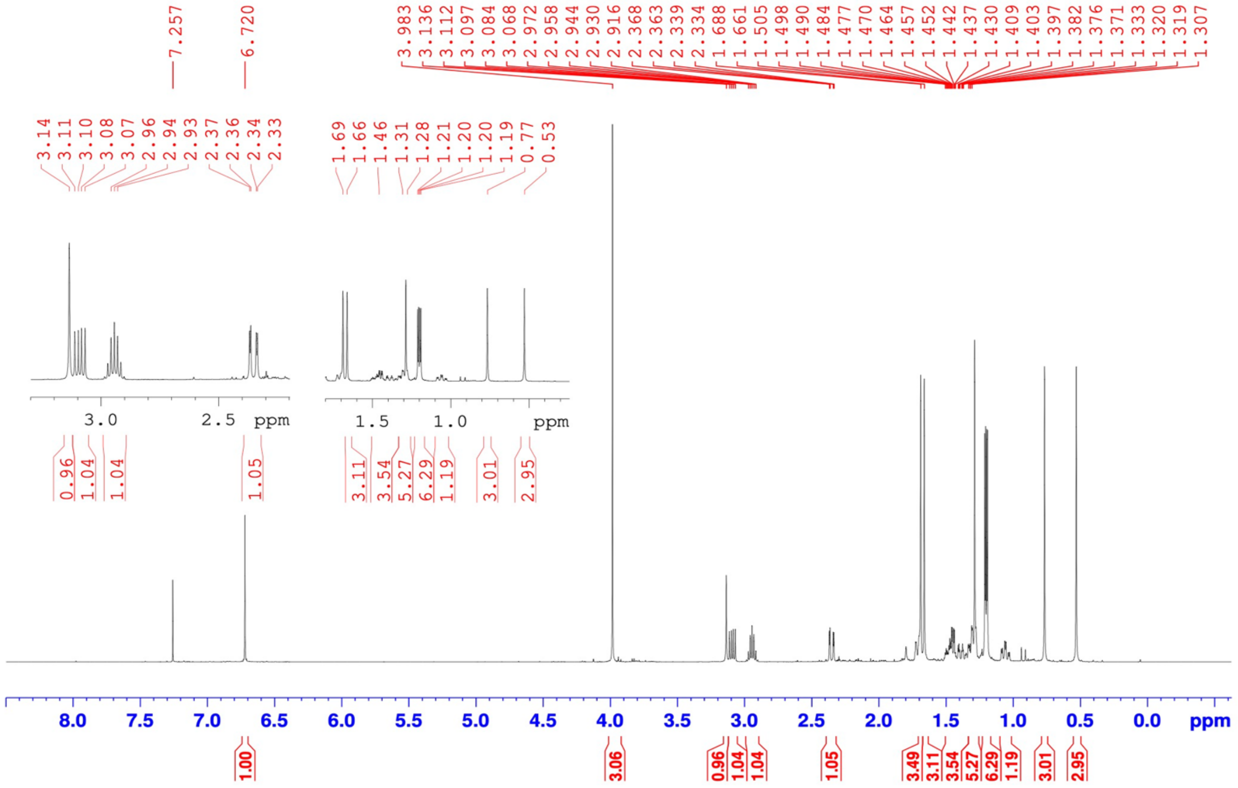

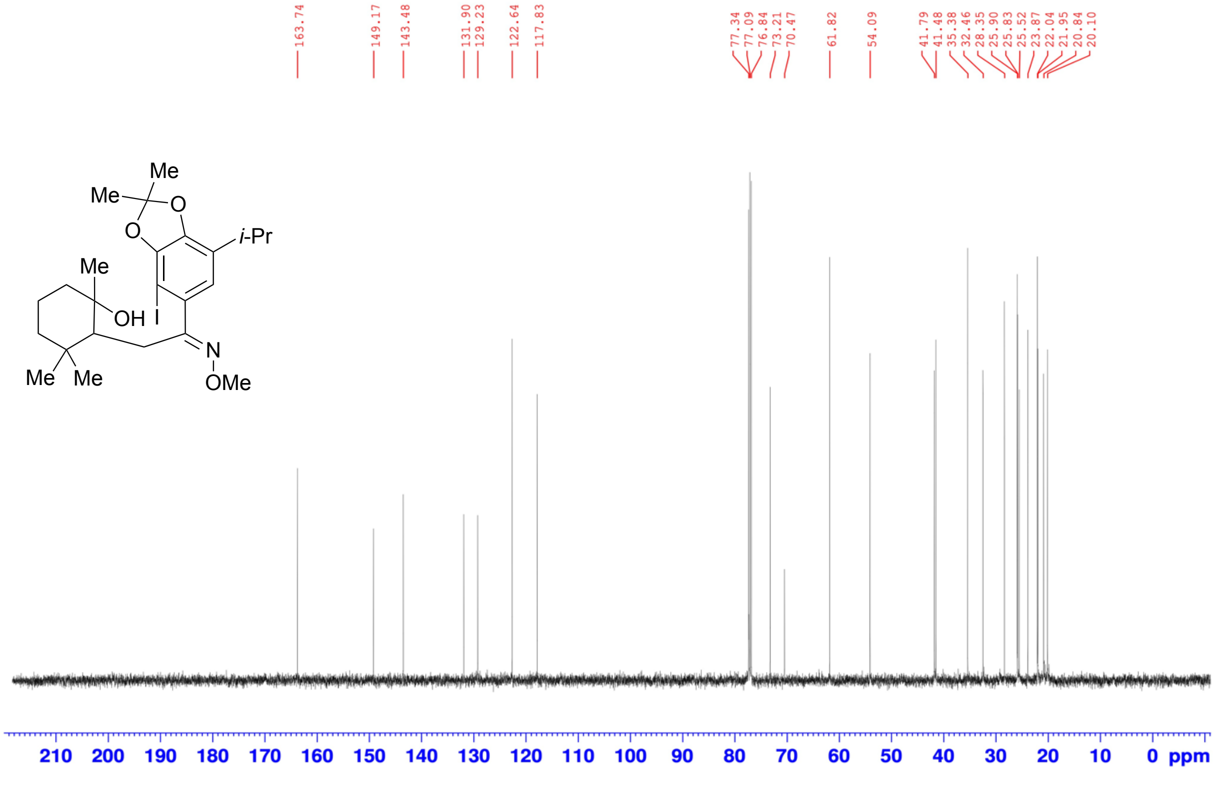

^{1}H and ^{13}C NMR spectra of **6a (I)**

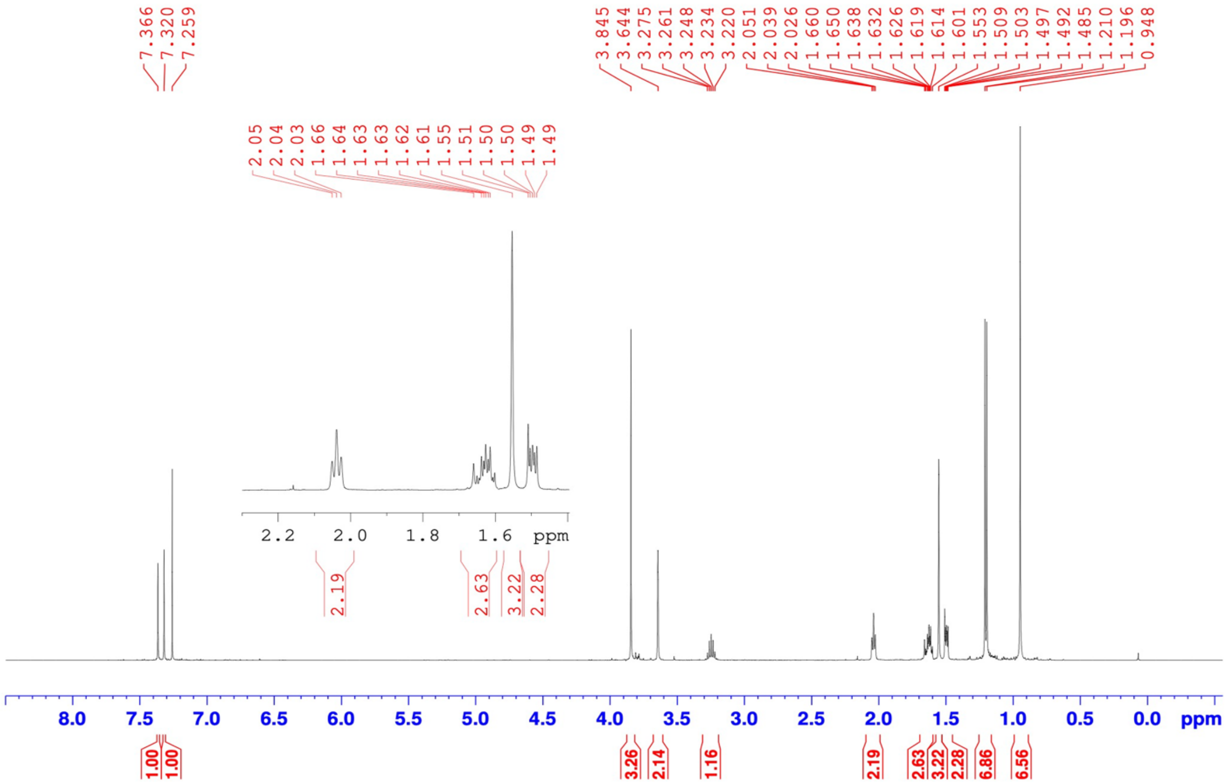

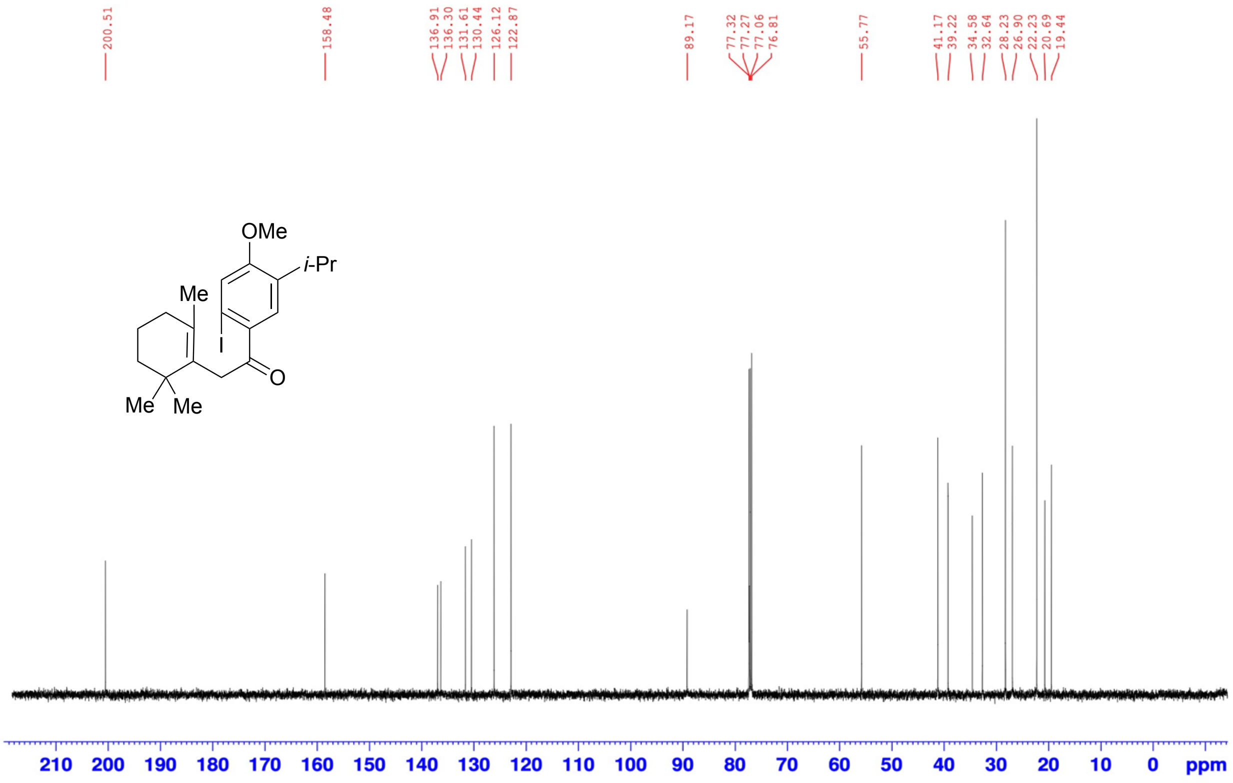

^{1}H and ^{13}C NMR spectra of **6b (I)**

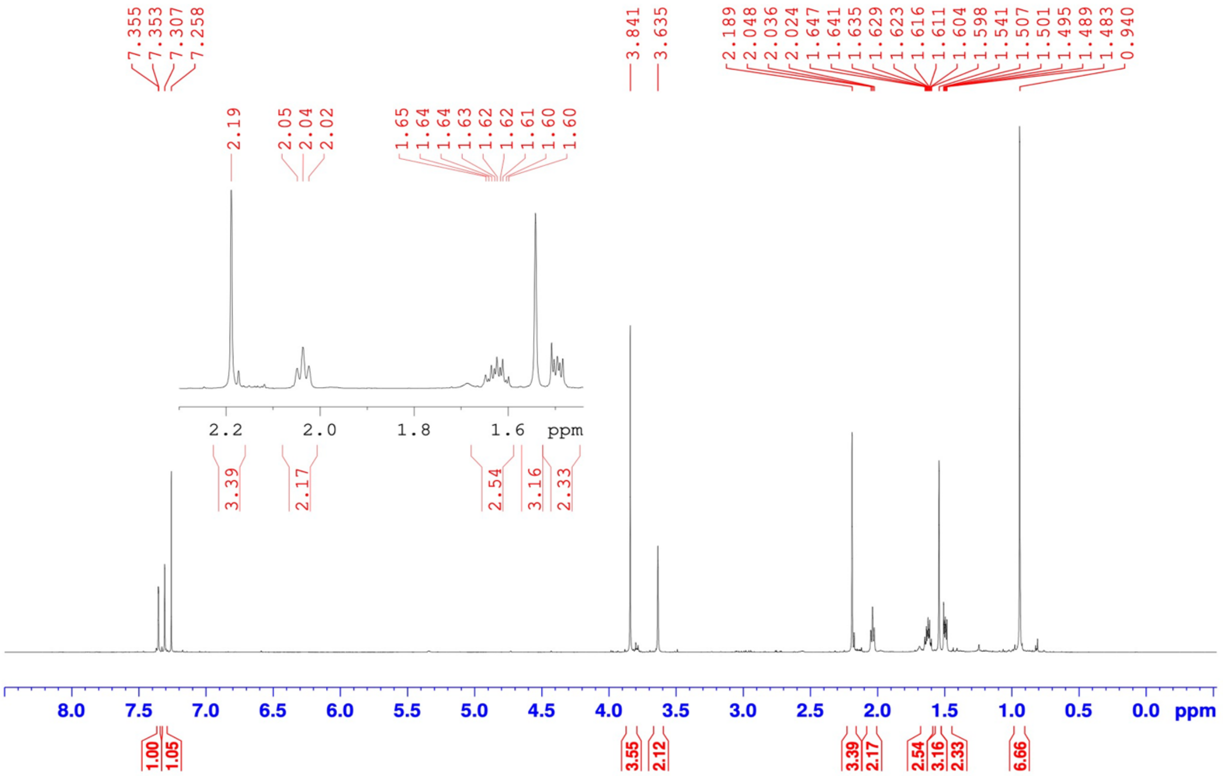

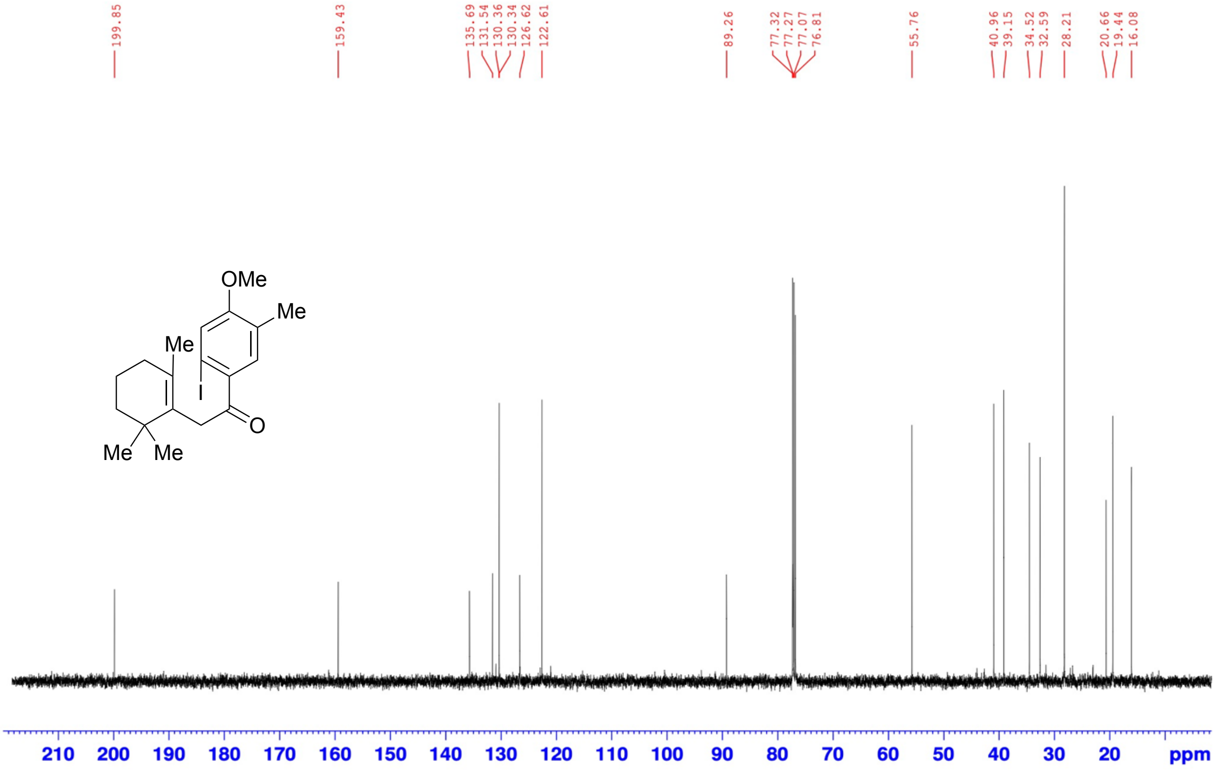

^{1}H and ^{13}C NMR spectra of **6c (I)**

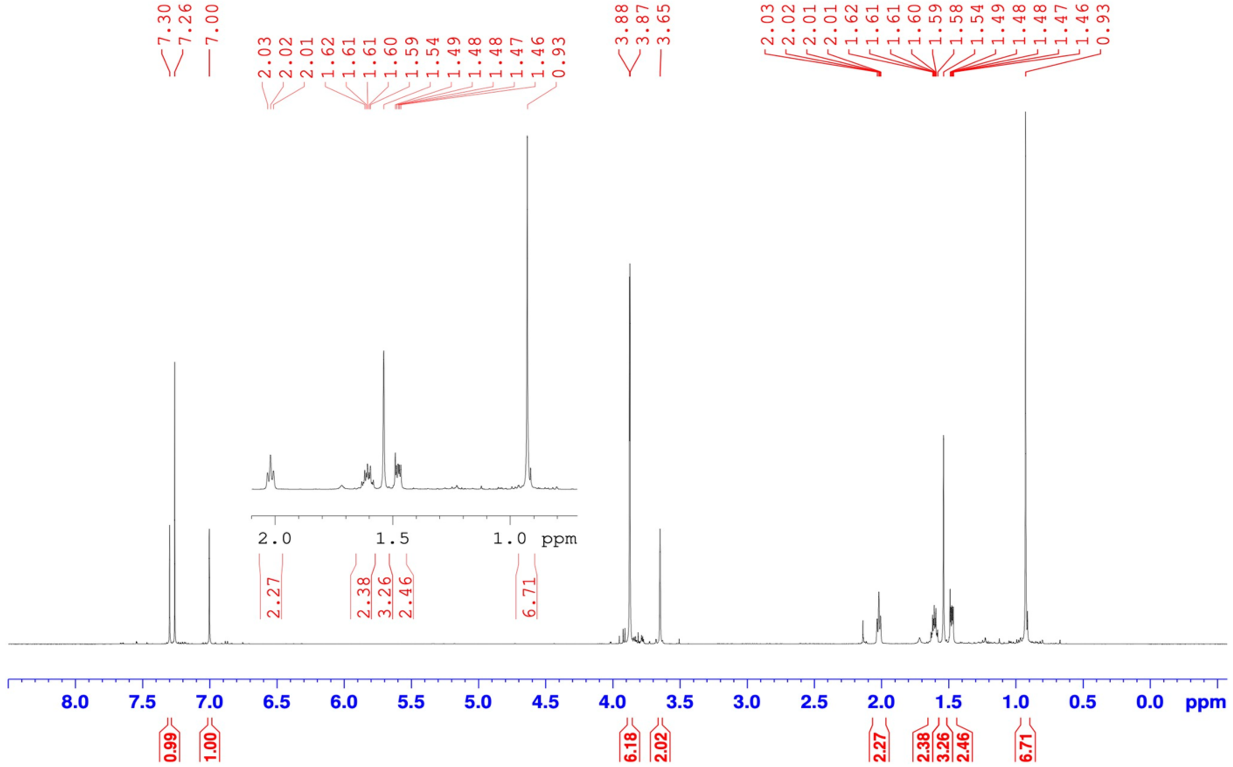

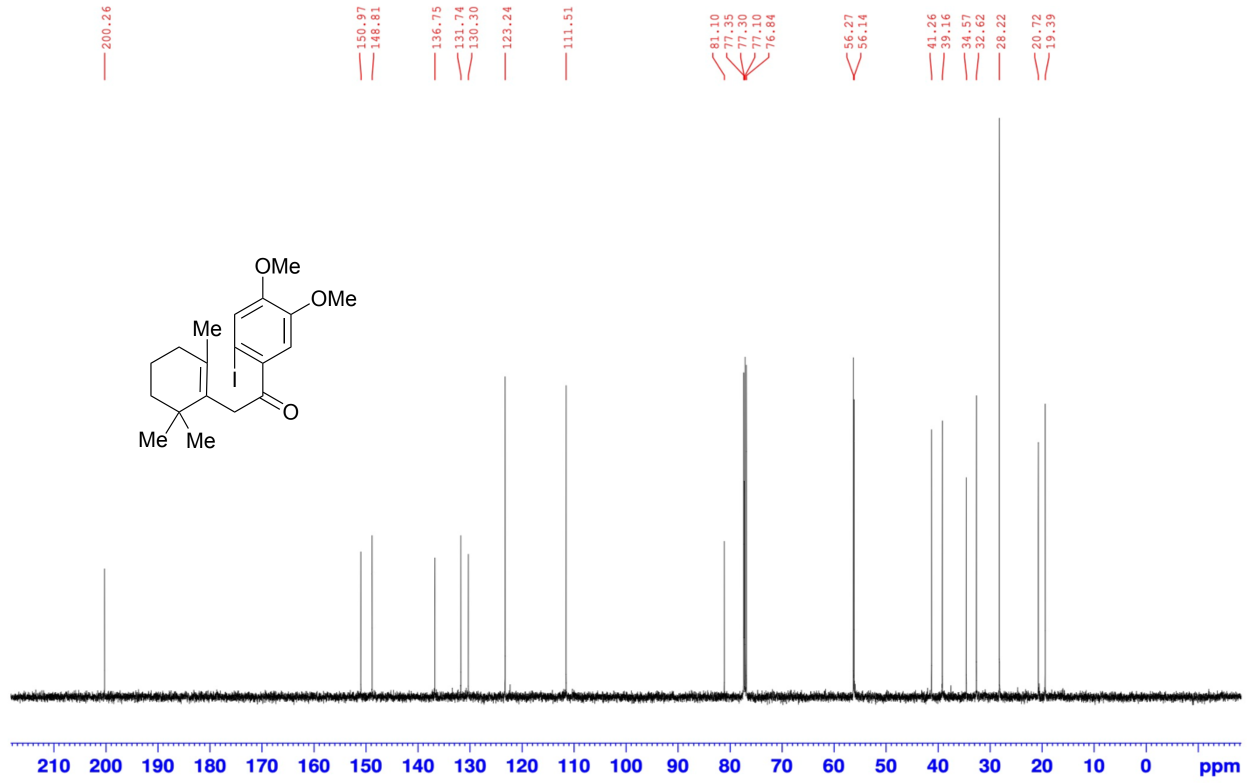

^{1}H and ^{13}C NMR spectra of **6d (I)**

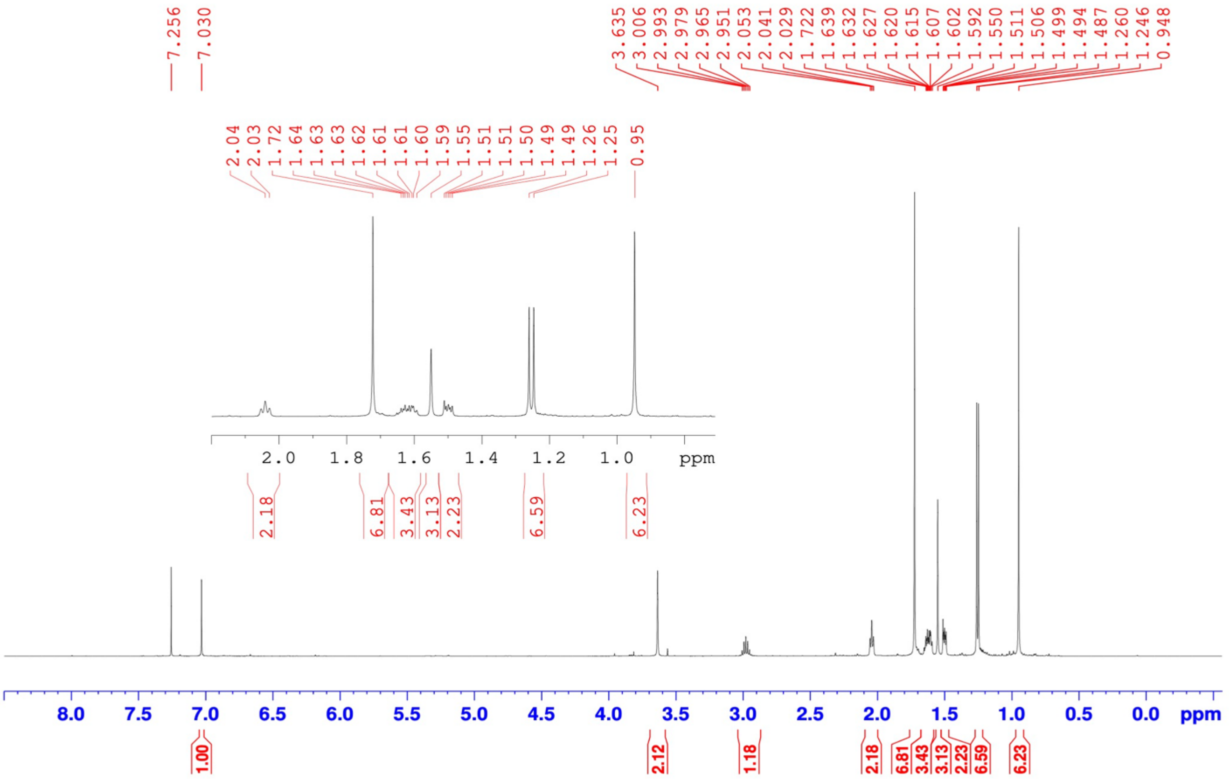

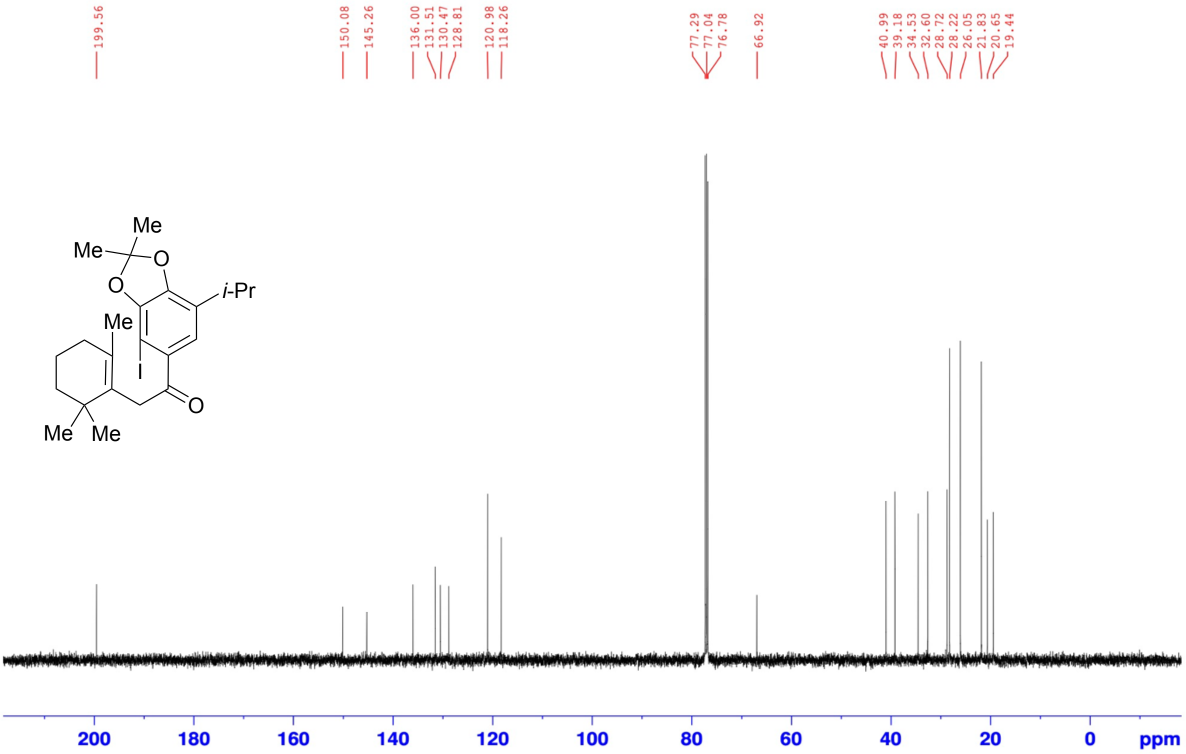

^{1}H and ^{13}C NMR spectra of **10a**

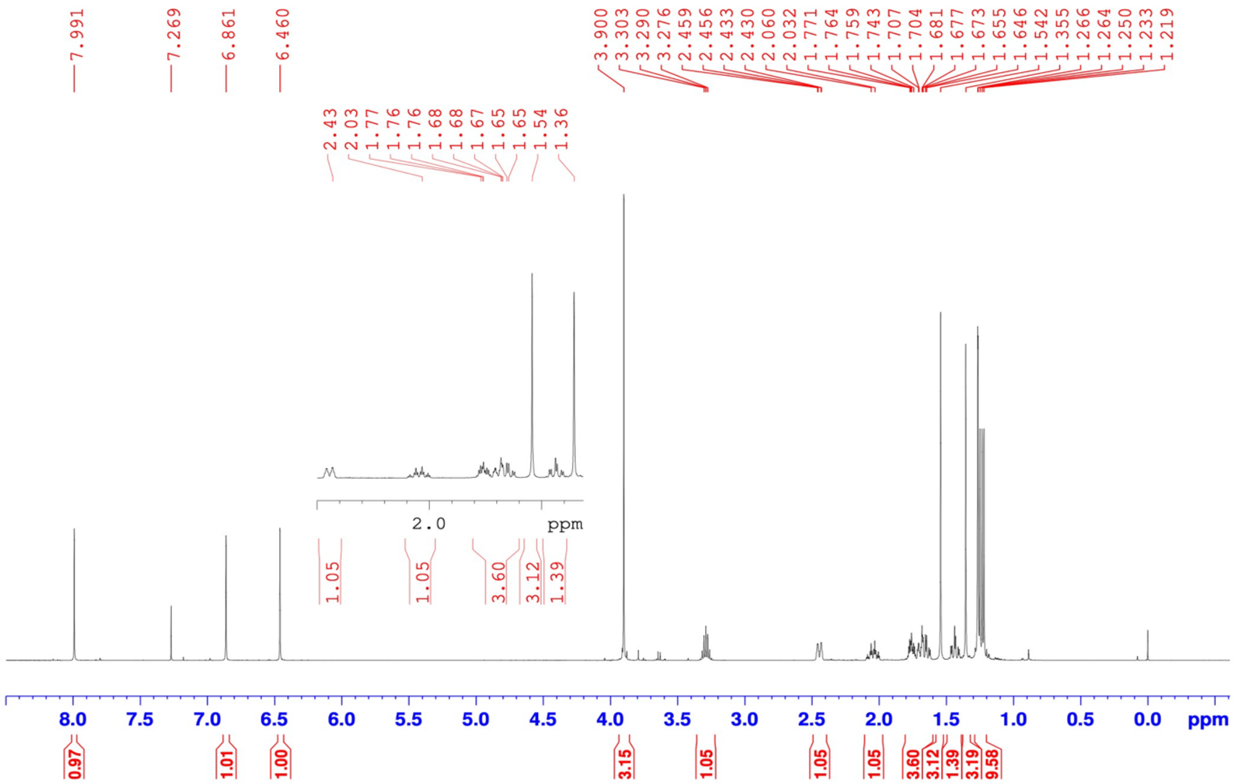

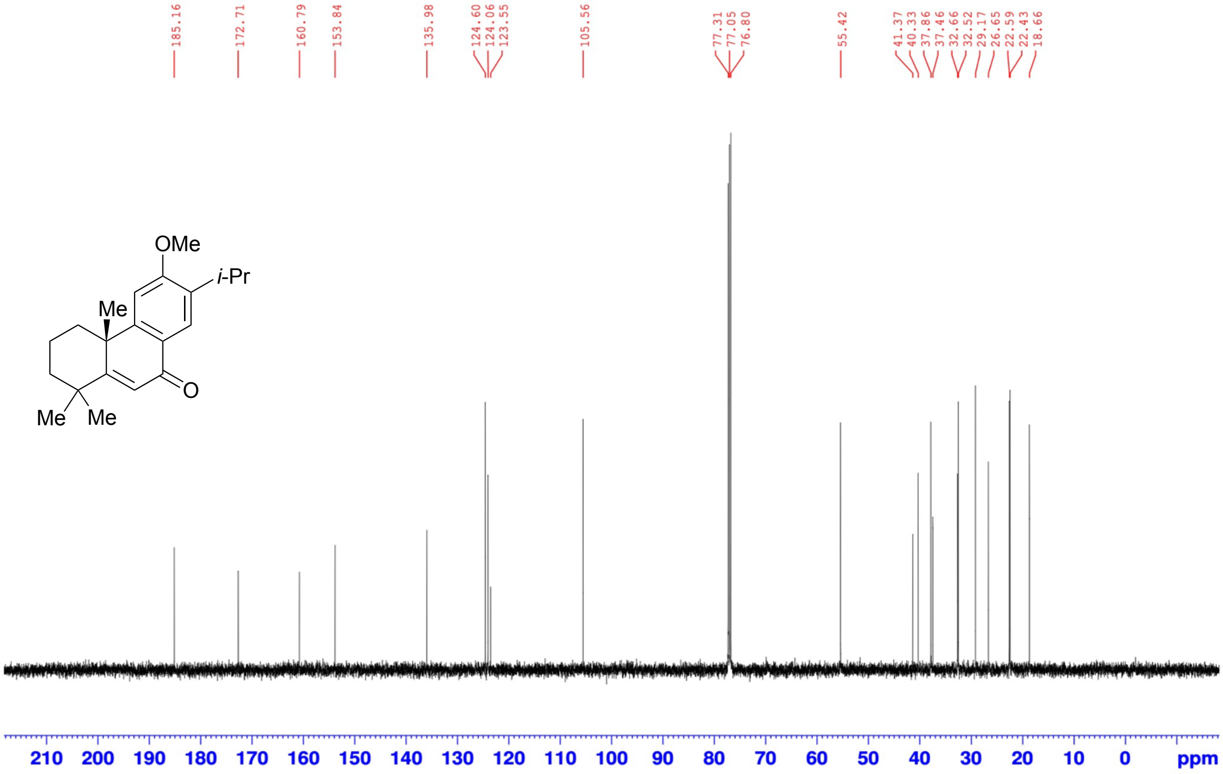

^{1}H and ^{13}C NMR spectra of **11a**

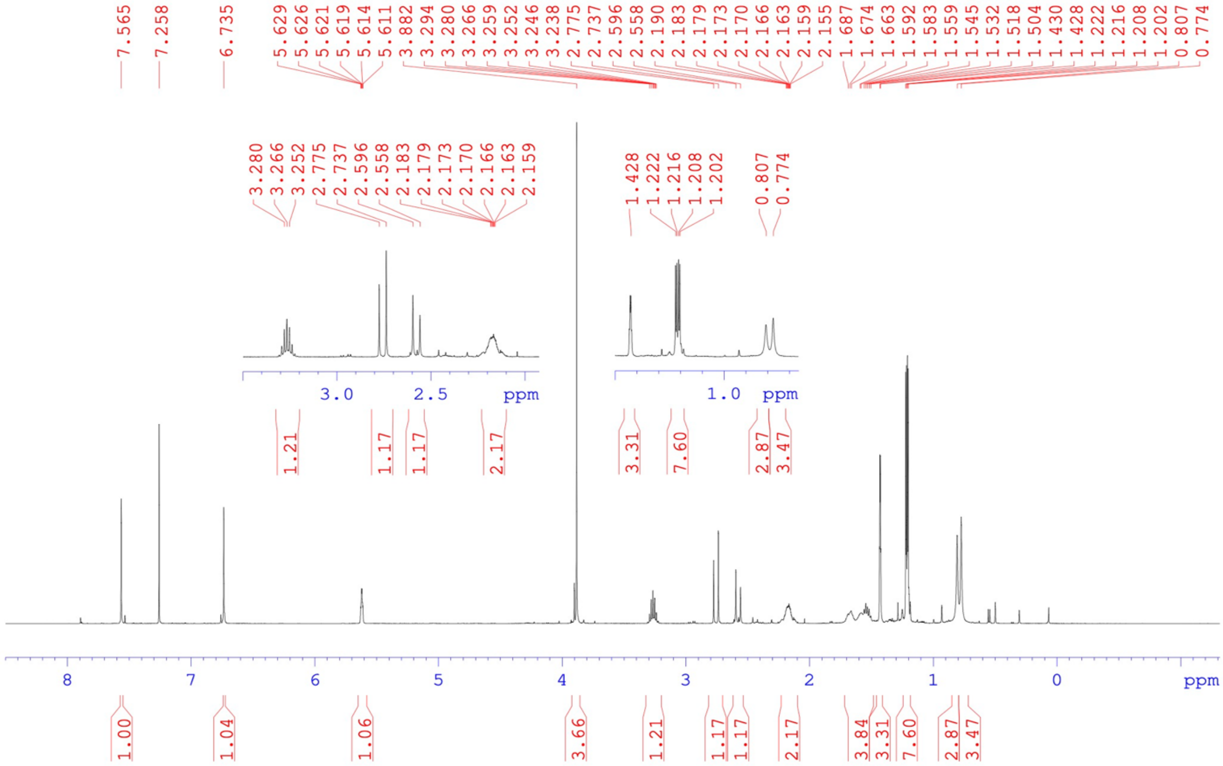

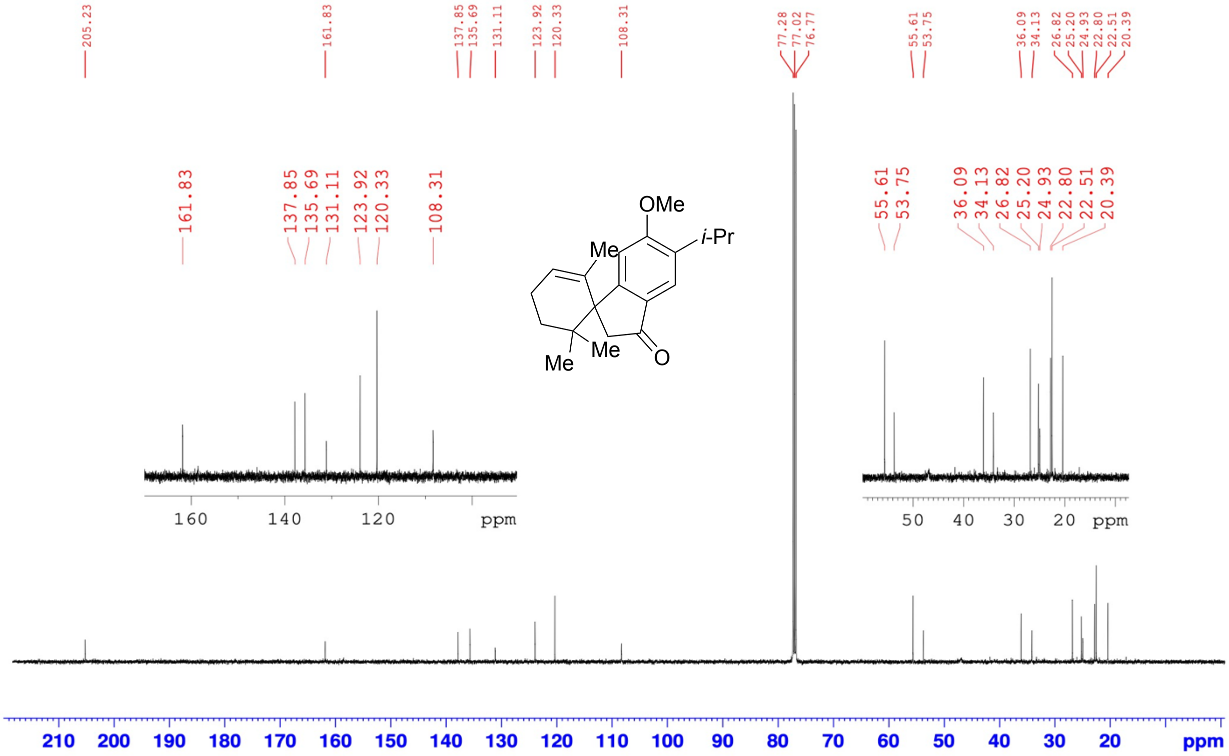

^{1}H and ^{13}C NMR spectra of **10b**

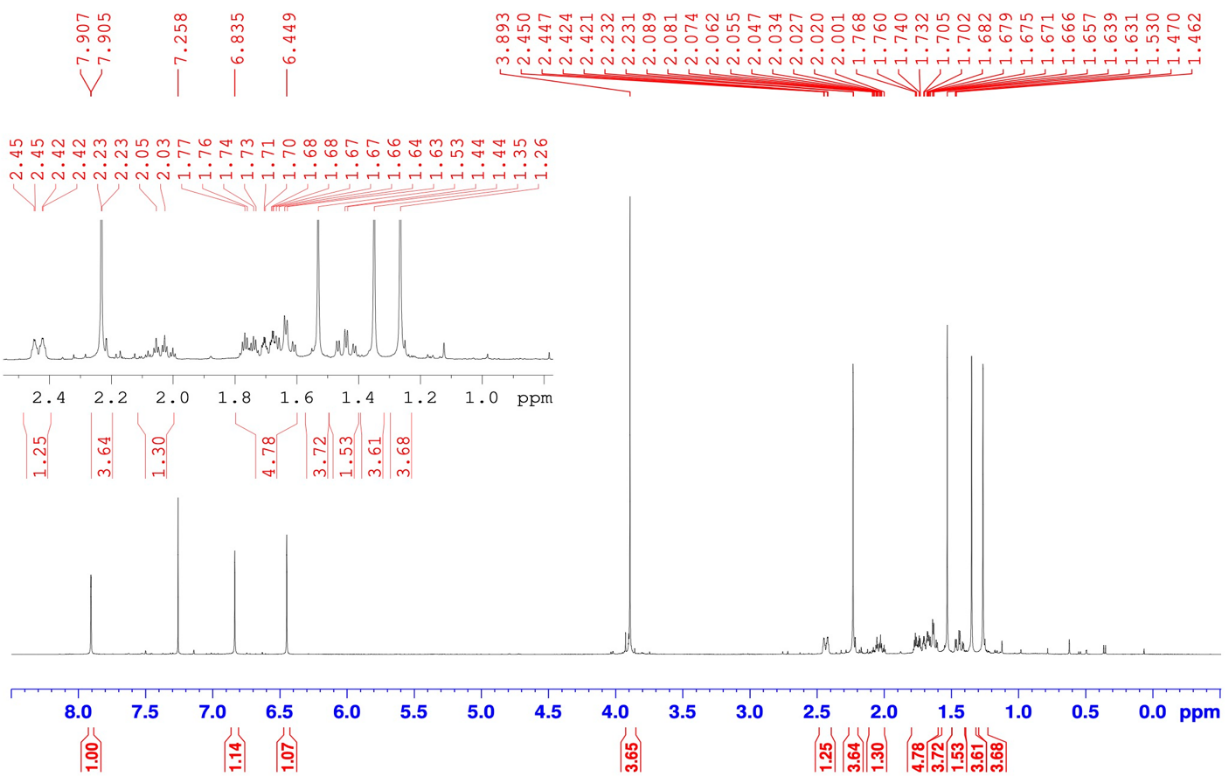

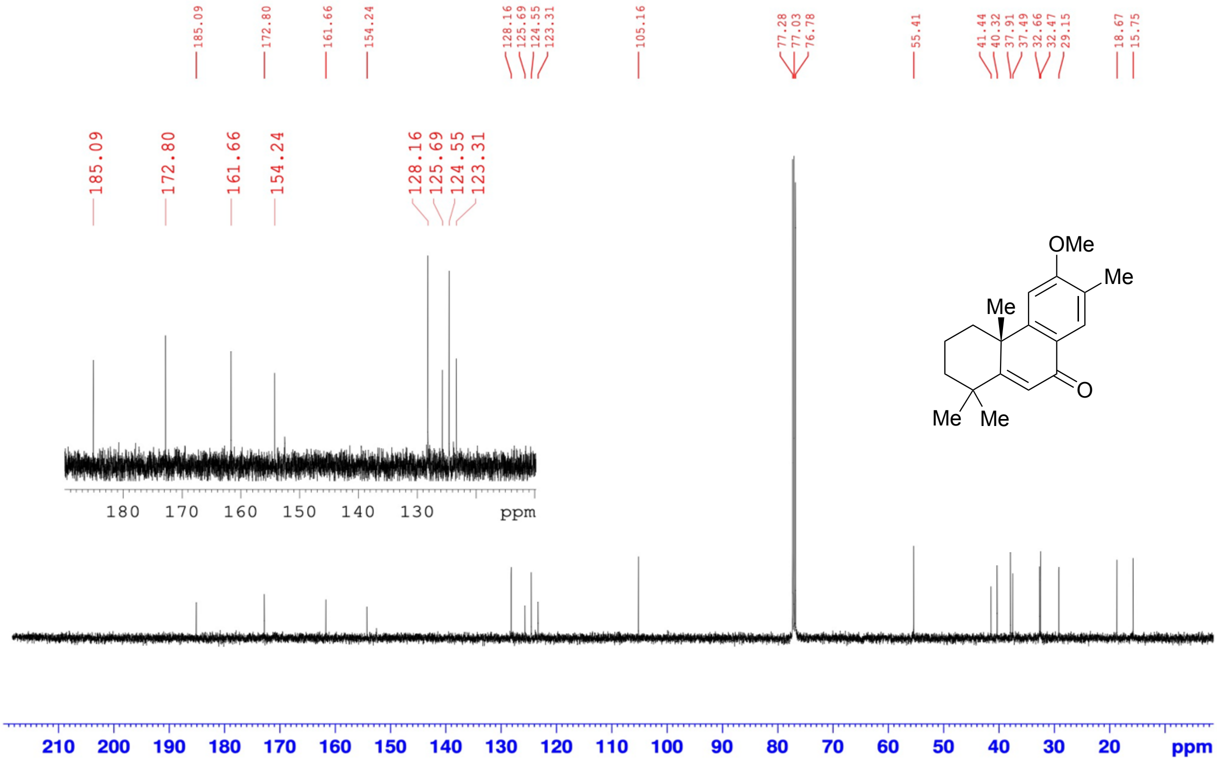

^{1}H and ^{13}C NMR spectra of **10c**

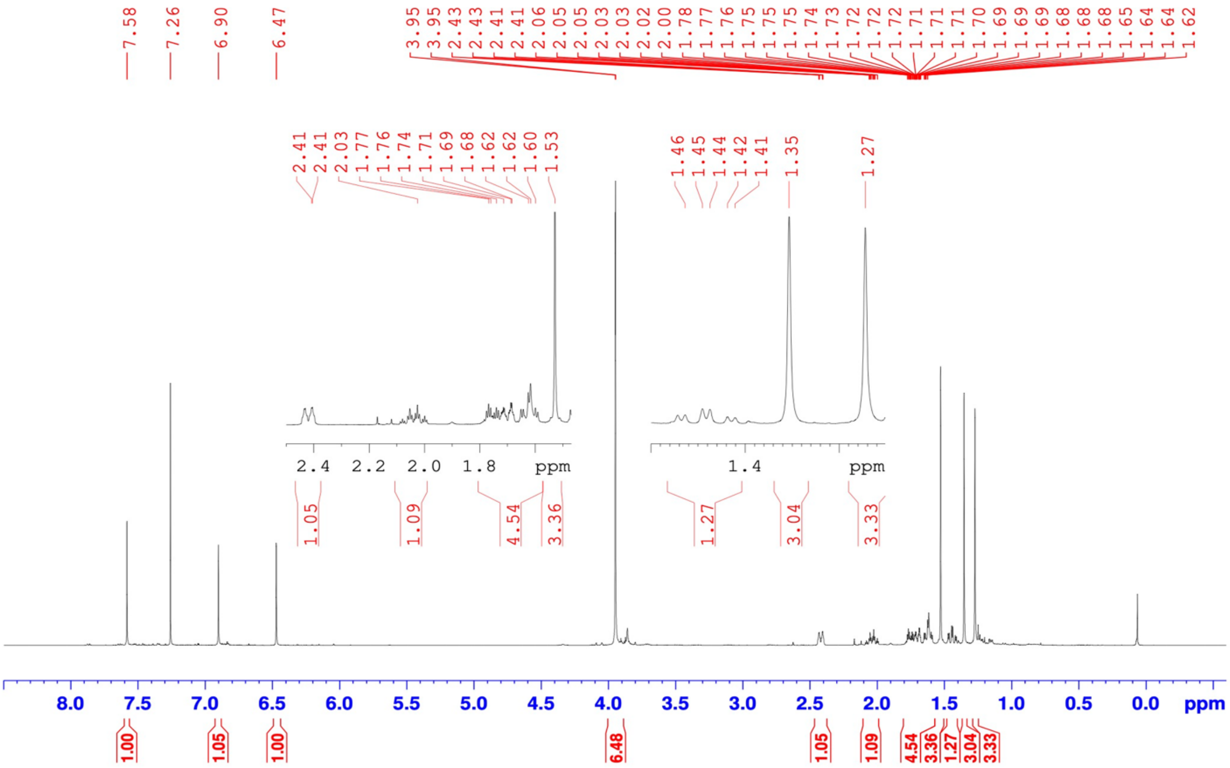

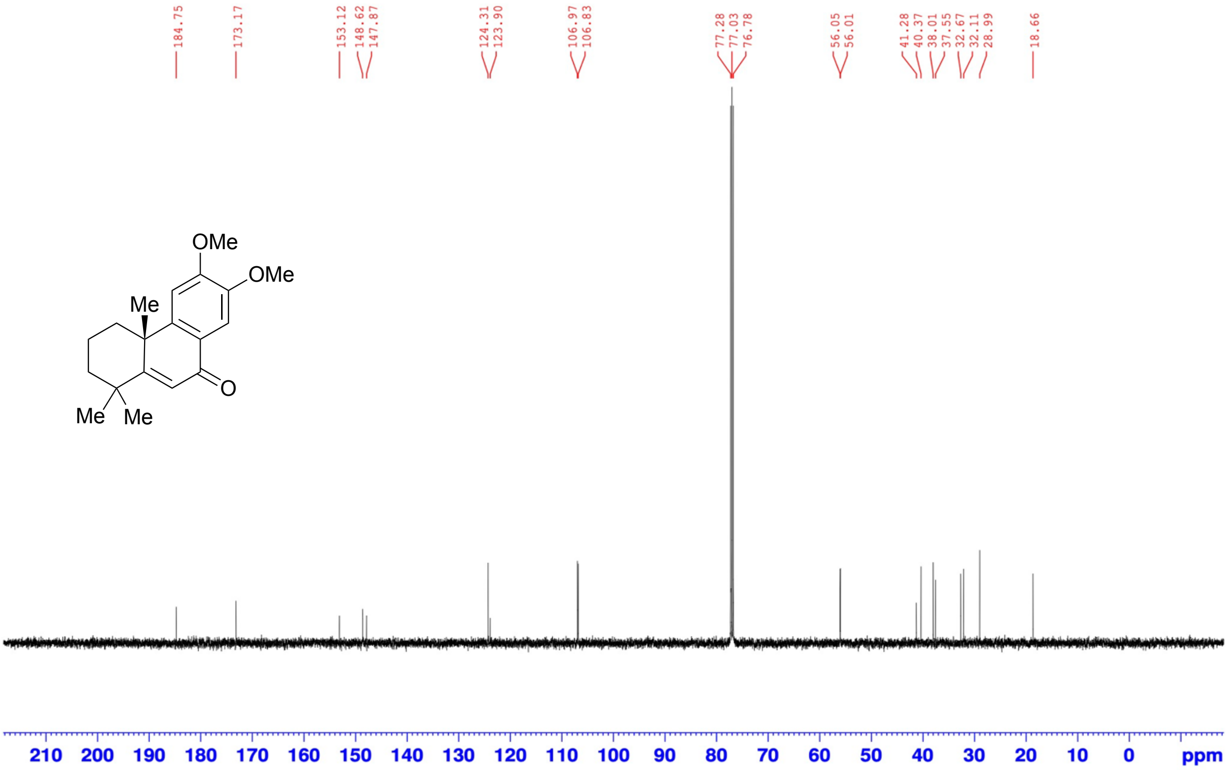

^{1}H and ^{13}C NMR spectra of **10d**

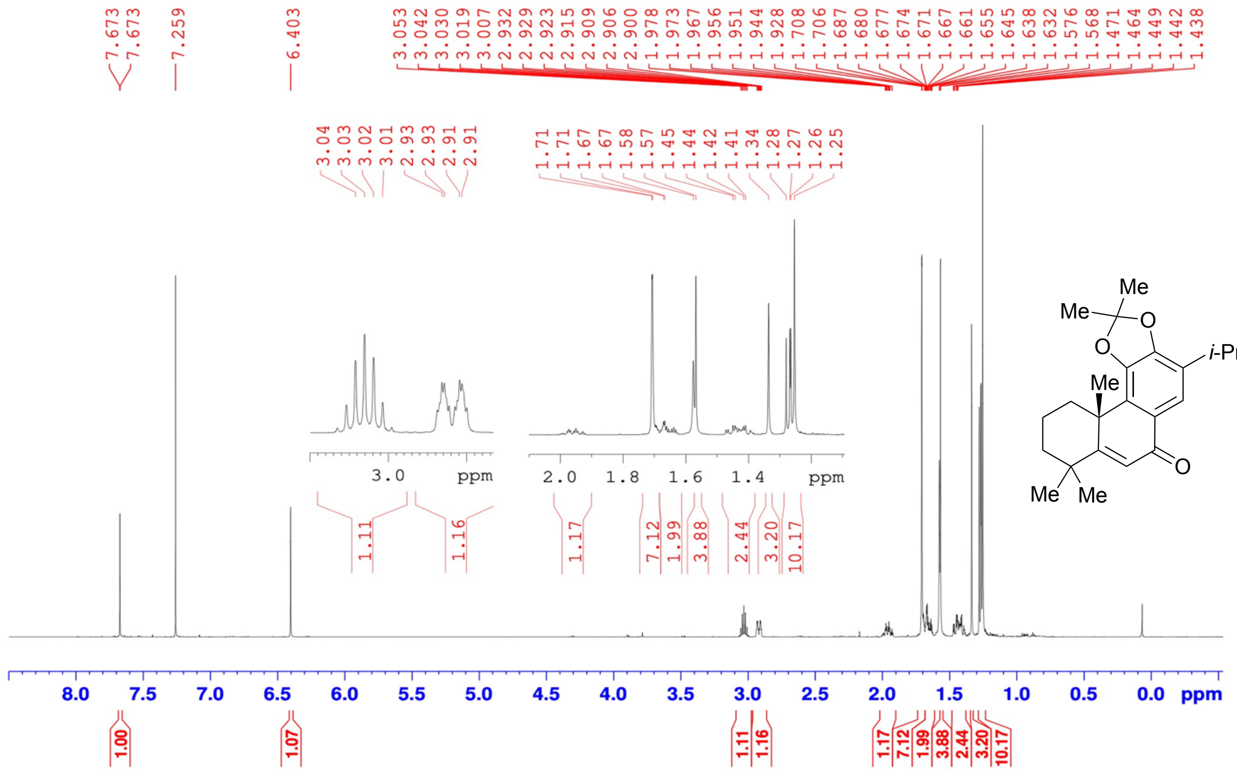

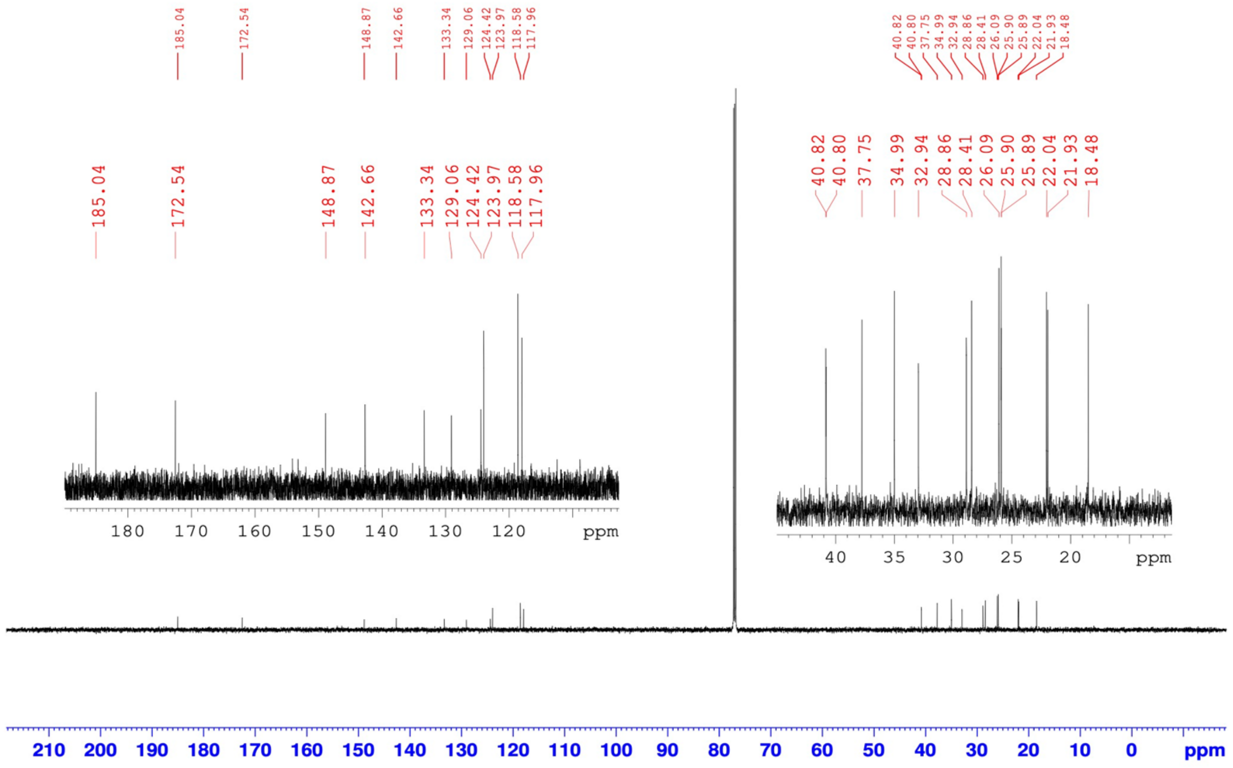

^{1}H and ^{13}C NMR spectra of **1a**

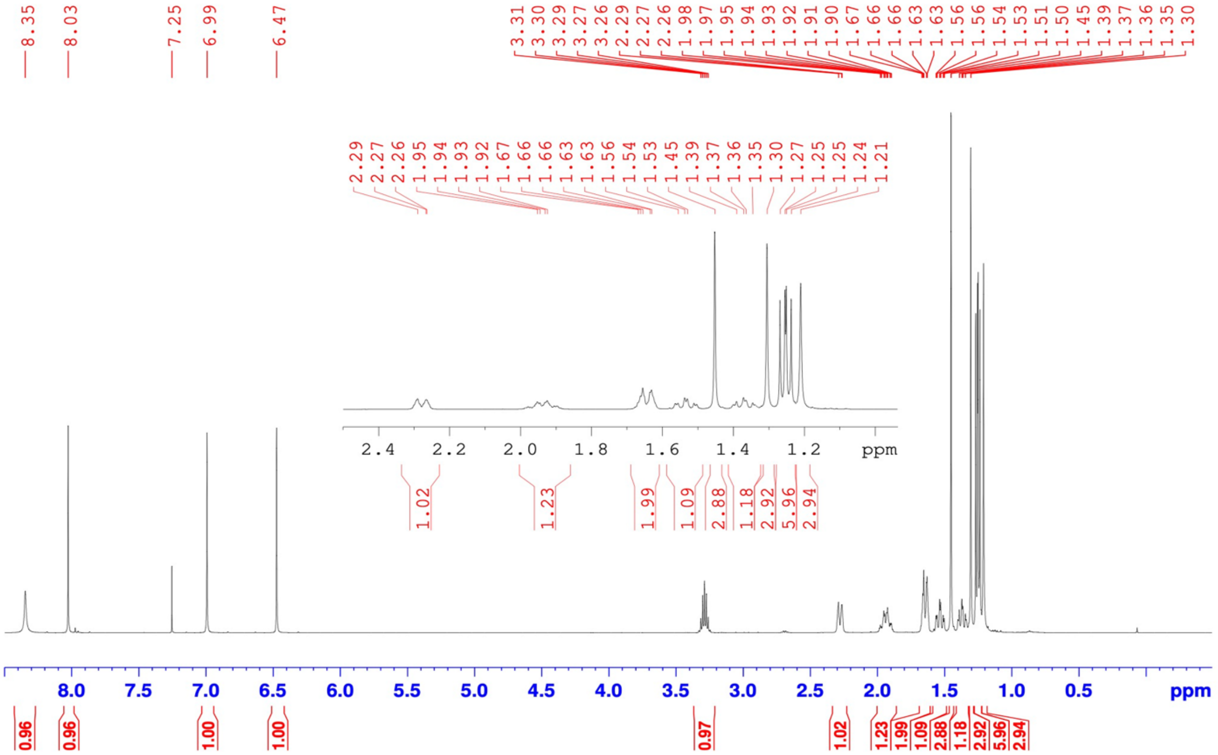

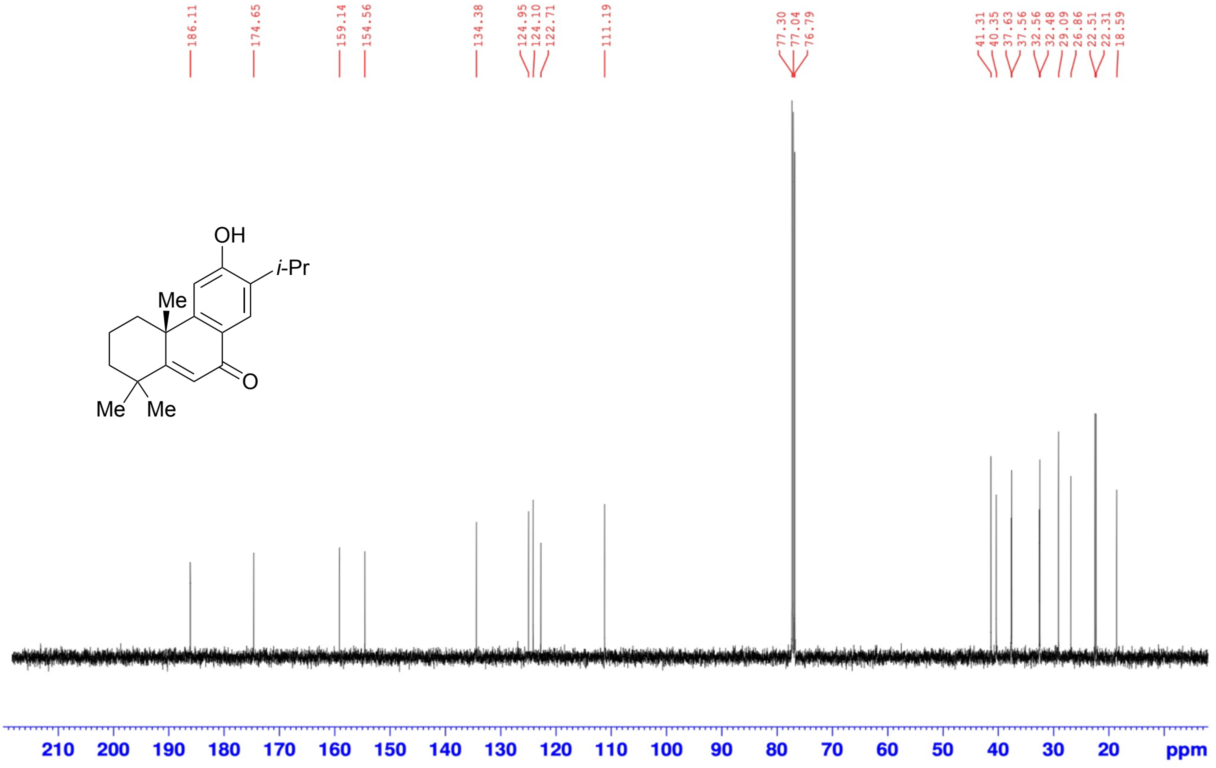

^{1}H and ^{13}C NMR spectra of **1b**

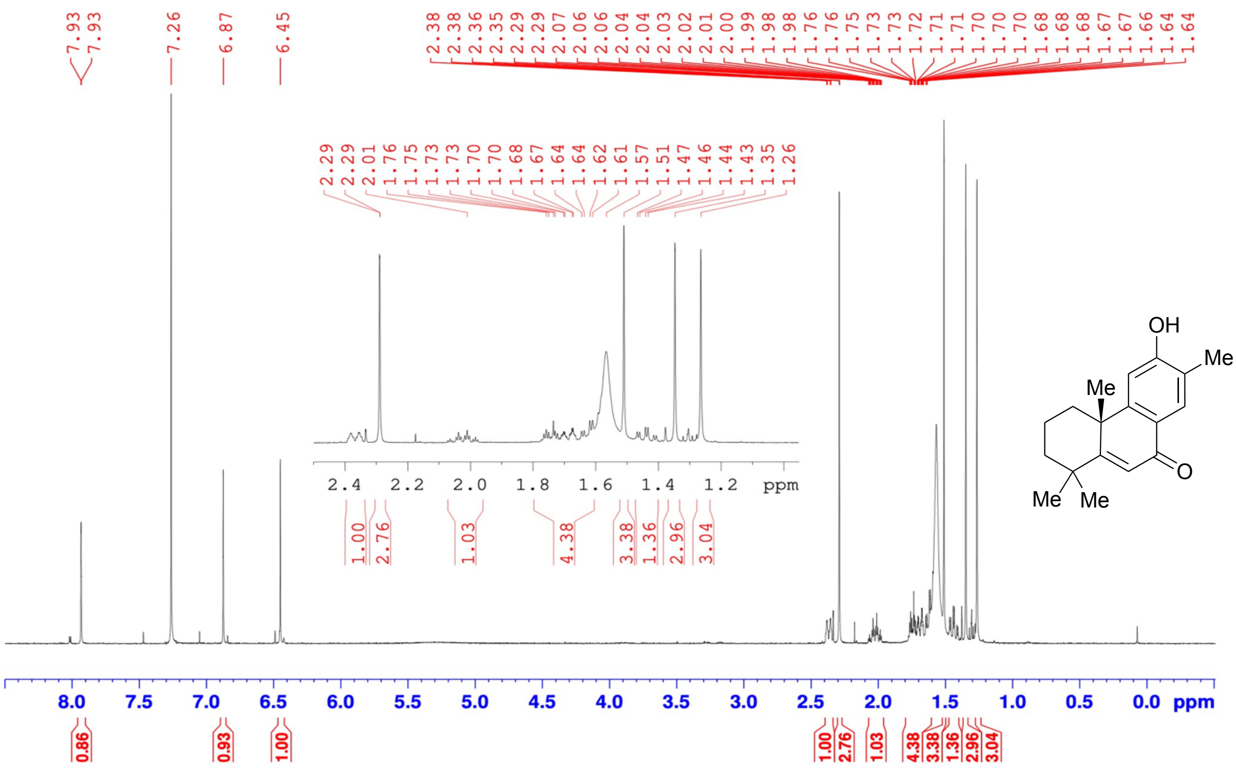

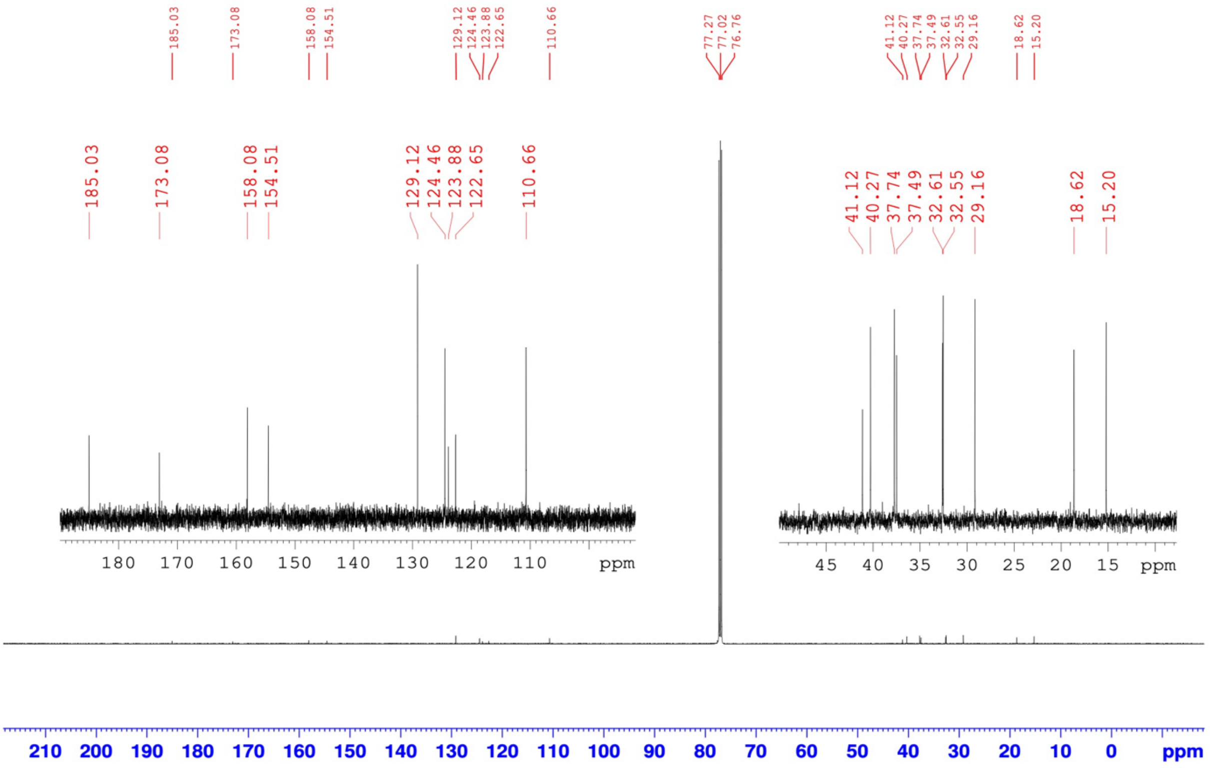

^{1}H and ^{13}C NMR spectra of **1c**

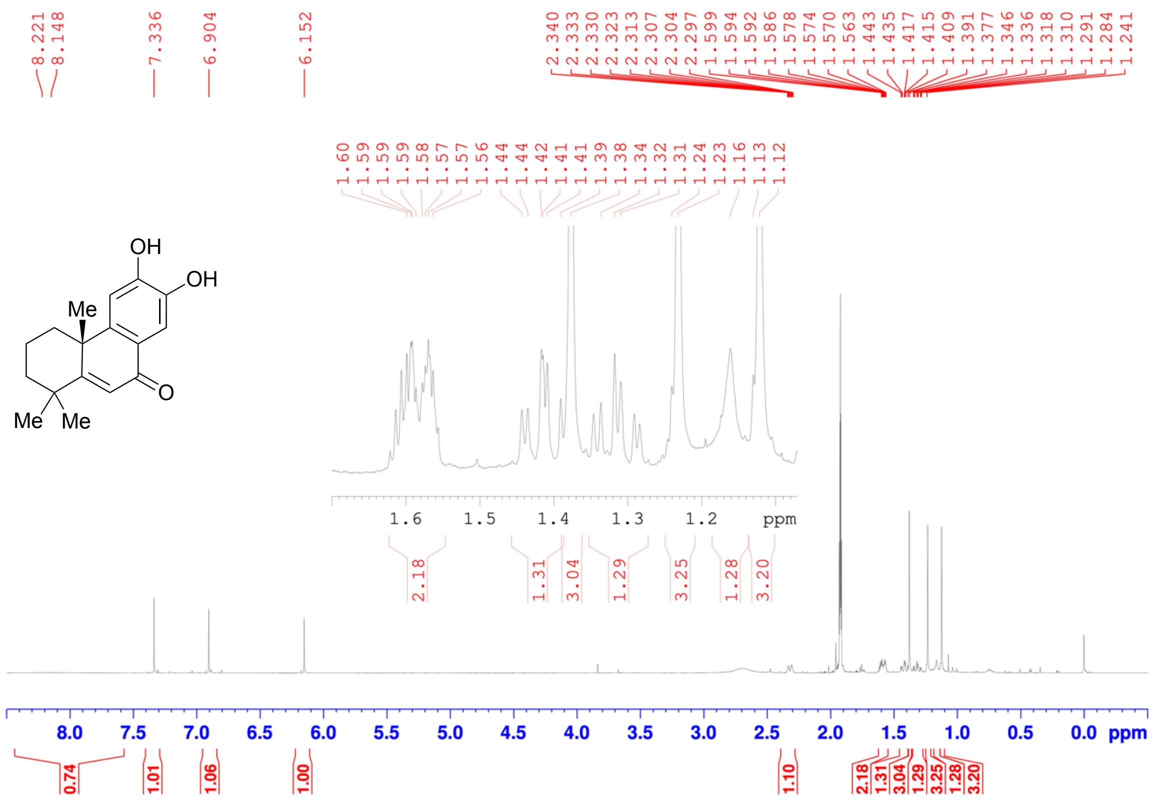

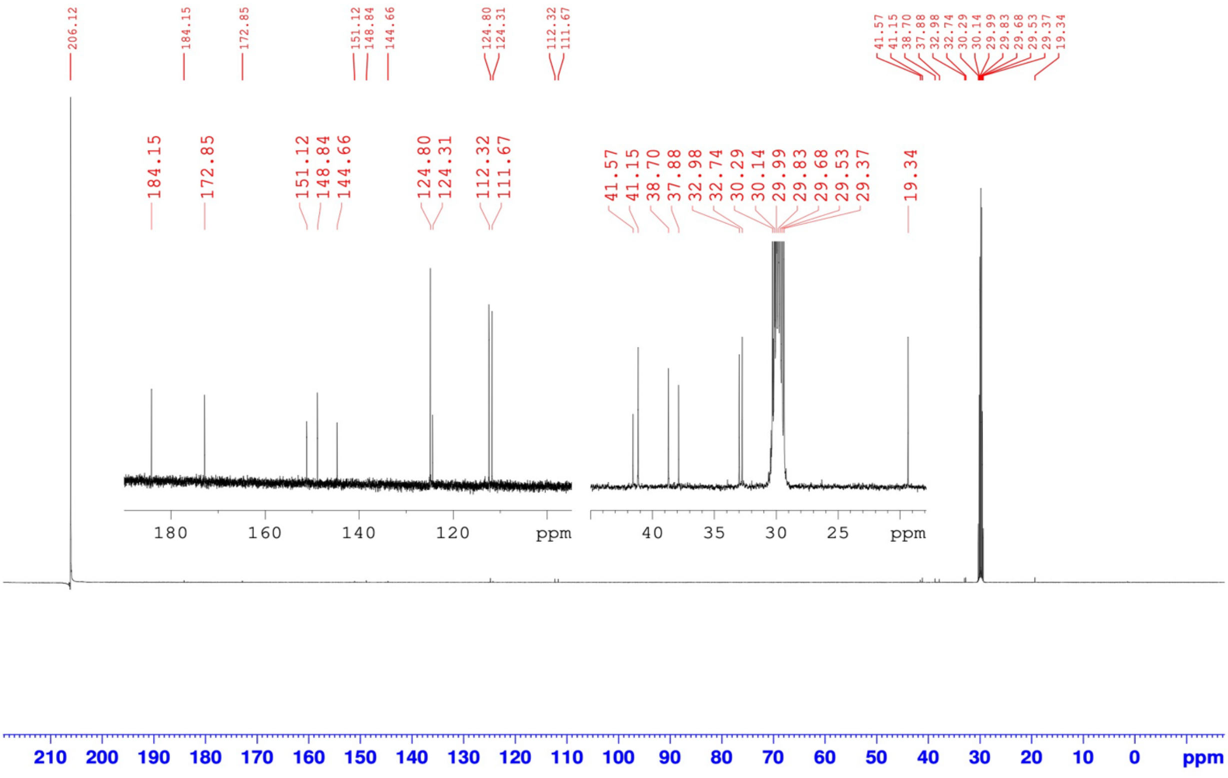

^{1}H and ^{13}C NMR spectra of **1d**

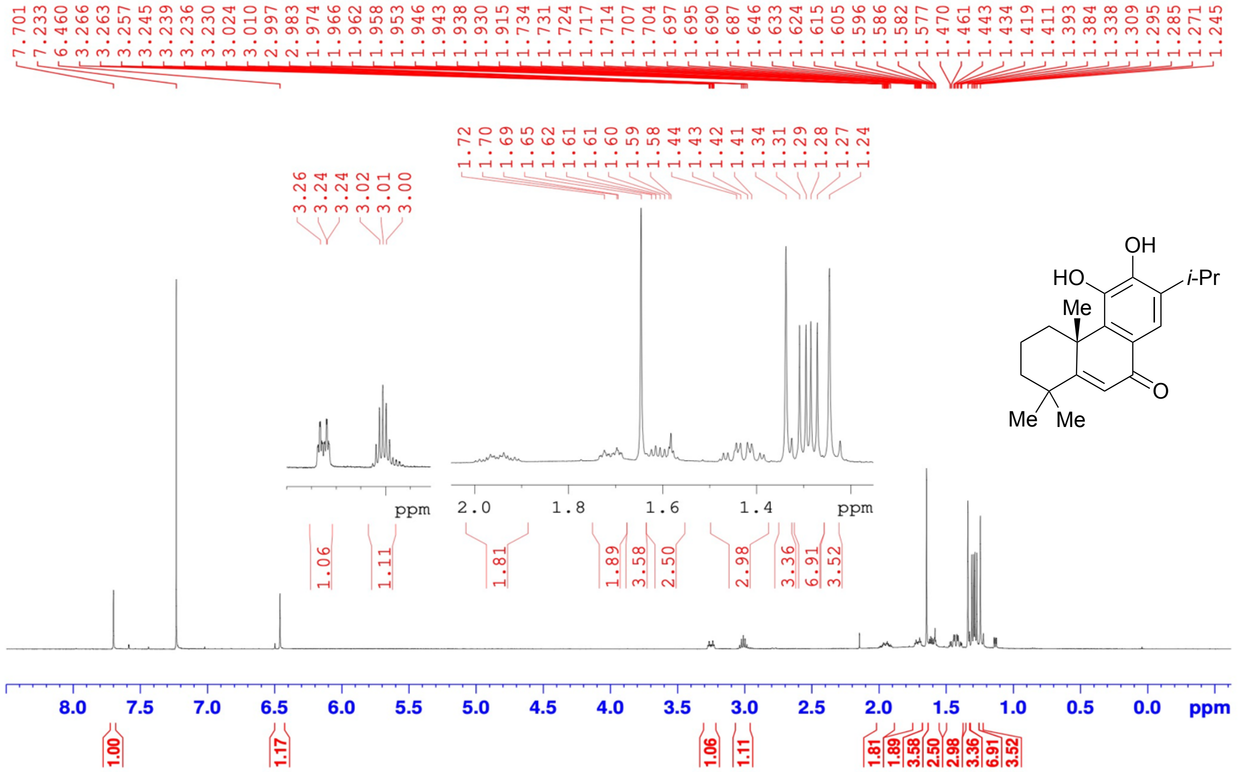

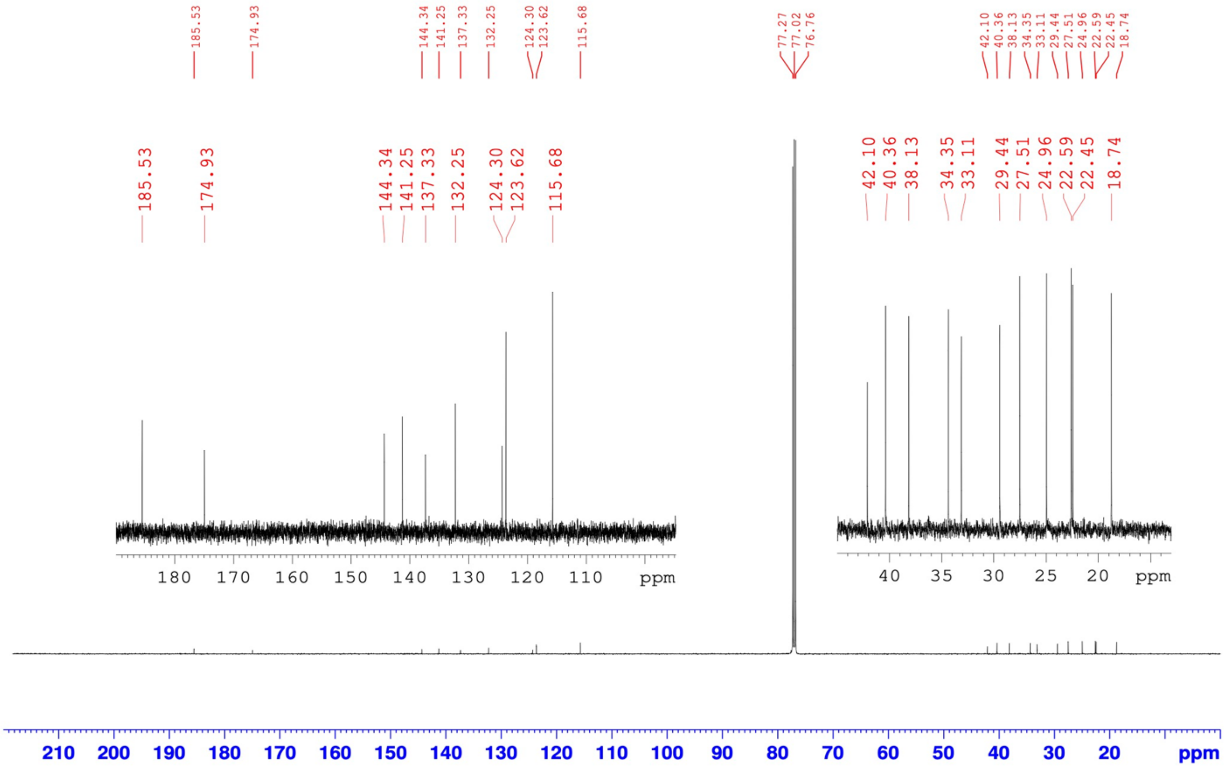

^{1}H and ^{13}C NMR spectra of **12**

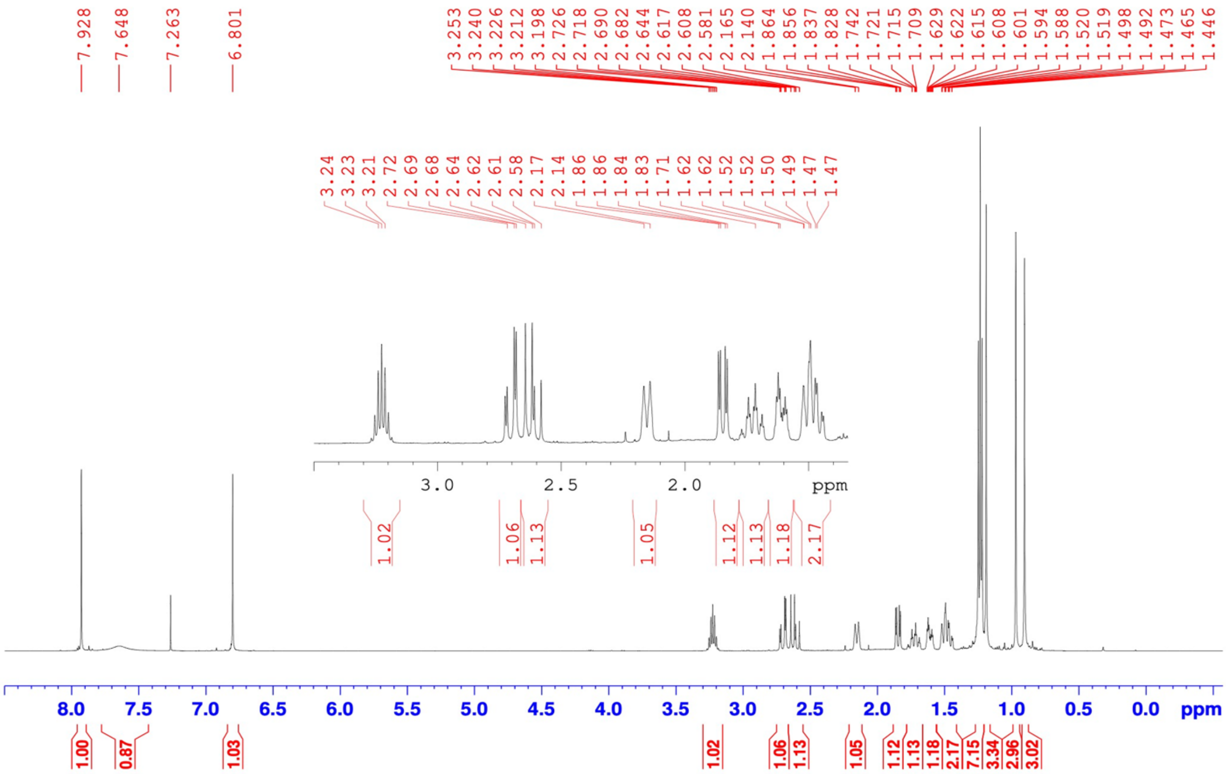

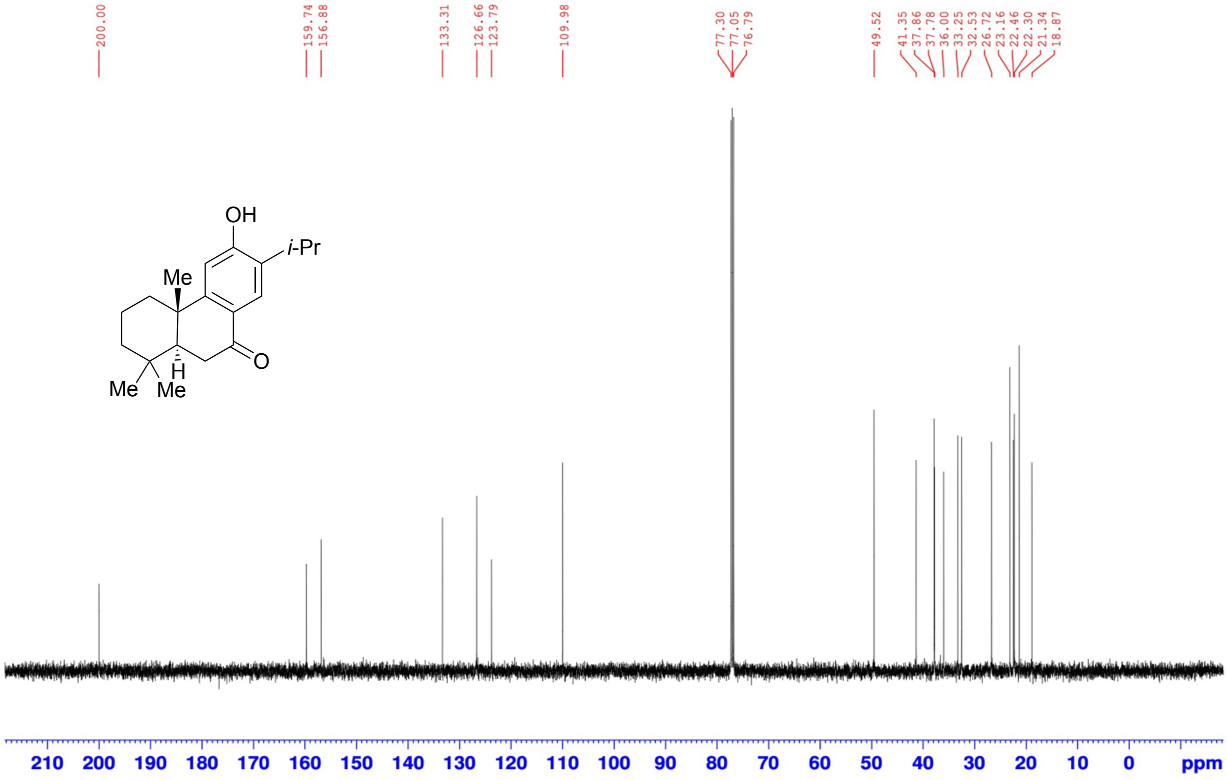

^{1}H and ^{13}C NMR spectra of **13**

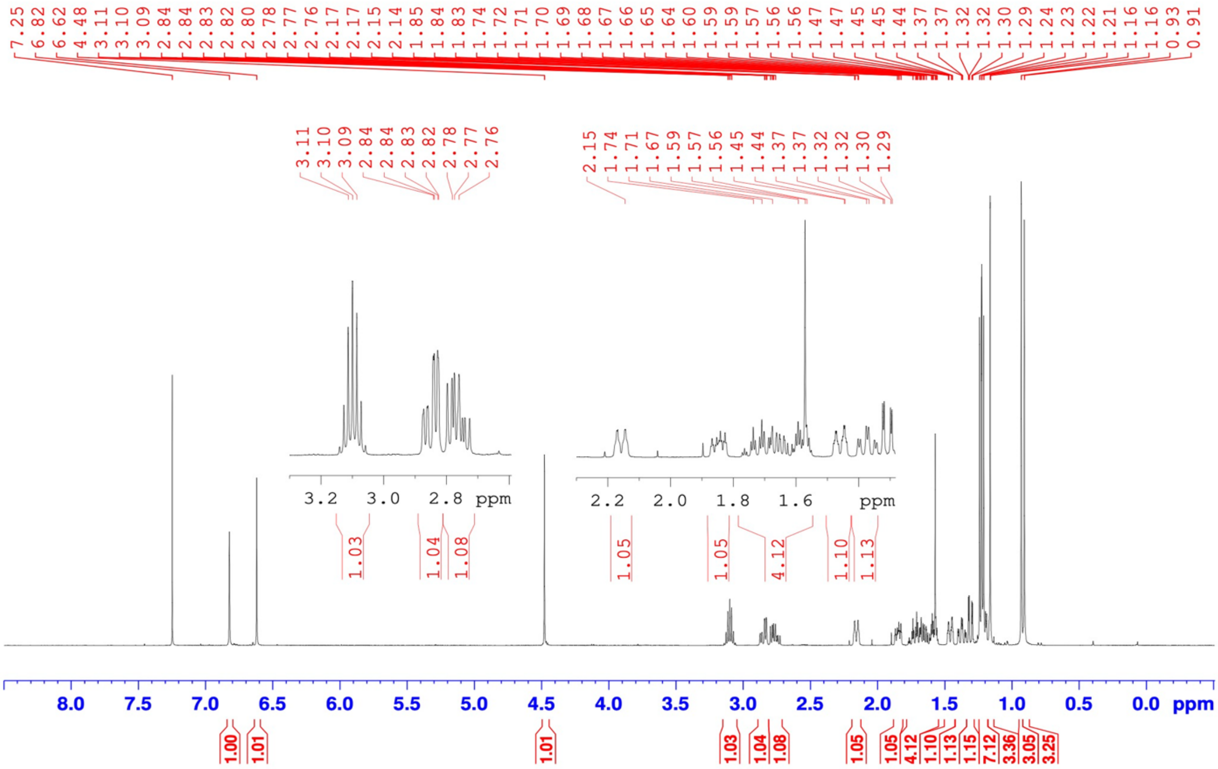

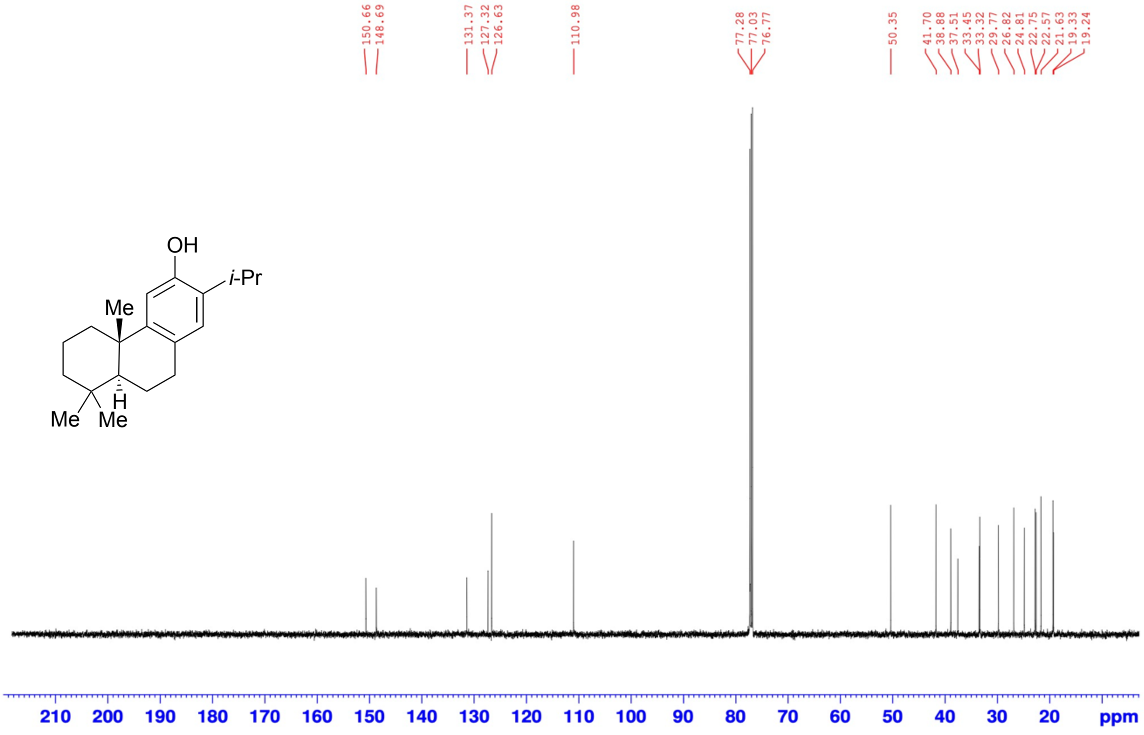

Supplement: Supplementary file 3 — Supplementary Data 1 [file 42004_2023_979_MOESM3_ESM.pdf]
