# Supplementary material for: Unified short syntheses of oxygenated tricyclic aromatic diterpenes by radical cyclization with a photoredox catalyst
Source: Commun Chem. 2023 Aug 21;6:169. doi: 10.1038/s42004-023-00979-2 (PMC10442340; doi:10.1038/s42004-023-00979-2)
Supplement: Supplementary file 4 — Supplementry Data 2 [file 42004_2023_979_MOESM4_ESM.pdf]

Supplementary Data 2

Unified short syntheses of oxygenated tricyclic aromatic diterpenes by radical cyclization with a photoredox catalyst

Riichi Hashimoto,[1]* Kengo Hanaya,[1] Takeshi Sugai,[1] and Shuhei Higashibayashi[1]*

[1]Faculty of Pharmacy, Keio University, 1-5-30 Shibakoen, Minato-ku, Tokyo 105-8512, Japan
E-mail: riichi8222hashimoto@keio.jp, higashibayashi-sh@pha.keio.ac.jp

Theoretical Calculation

DFT calculations were performed using the Gaussian 16 program package.[1] The structures were optimized at uM06-2X/6-311++G(d,p) level of theory with SMD solvation model (DMSO) and the vibrational frequency analyses were conducted on the optimized structures. The given energies are zero-point corrected.

A

$E = -967.459444$ a.u.

C	-0.54777445	-0.87582399	0.36874121
C	-0.23611990	0.45984855	0.29797919
C	-1.11270102	1.47707993	0.00671496
C	-2.44542683	1.11291238	-0.23086255
C	-2.85188221	-0.24593658	-0.17287773
C	-1.89800558	-1.20390384	0.11980226
H	-0.78687481	2.50921017	-0.03809468
H	-2.17471425	-2.25162951	0.16056581
O	-3.40968225	2.00369834	-0.53190250
C	-3.05126682	3.37895931	-0.62112207
H	-3.96591731	3.90897233	-0.87766372
H	-2.67169413	3.74591828	0.33582218
H	-2.30453332	3.53725215	-1.40329075
C	0.43305697	-1.96049191	0.65394111
O	0.08554745	-3.12508766	0.62057716
C	1.86577585	-1.62081928	1.06172245
H	2.45248488	-2.50486764	0.80226520
H	1.84347285	-1.58677282	2.15448075
C	2.50571191	-0.37284663	0.49535238
C	2.56728929	0.77573657	1.19318781
C	3.11290102	-0.52237085	-0.90490086
C	3.20277270	2.03380397	0.64853575
C	3.36588373	0.85711075	-1.53233277
C	4.07688403	1.80602328	-0.57715440
H	3.77106569	2.51649915	1.45141220
H	2.38828404	2.73062258	0.40167509
H	3.93794984	0.72488250	-2.45648343
H	2.39851052	1.29628190	-1.81016067
H	4.28913277	2.75878812	-1.06920766
H	5.03976519	1.38362753	-0.27456843
C	2.16874751	-1.29645861	-1.83789082
H	2.01448751	-2.32705488	-1.50663961
H	1.19450385	-0.80574607	-1.91746356
H	2.60650859	-1.33811043	-2.84000289
C	4.43370842	-1.30830216	-0.81053495
H	4.27323623	-2.30535213	-0.39173063
H	4.86457263	-1.43362001	-1.80915957
H	5.16521841	-0.79584738	-0.18179089
C	1.95995869	0.98701746	2.55615961
H	1.38587374	1.92003229	2.55298918
H	1.29199153	0.19099614	2.88048204
H	2.74823028	1.10770978	3.30783267
C	-4.31335766	-0.58541404	-0.39984695
H	-4.66830774	0.05049508	-1.21596020
C	-4.54355747	-2.04192721	-0.79672425
H	-3.93694451	-2.32750892	-1.66003486
H	-4.31132986	-2.72353272	0.02633539
H	-5.59496142	-2.18724070	-1.05630975
C	-5.13237854	-0.24272867	0.85245104
H	-5.00852123	0.80396804	1.13728292
H	-6.19558337	-0.42631008	0.67459385
H	-4.81414621	-0.86935927	1.69138415

TS$_{A-B}$

$E = -967.456129$ a.u.

C	-0.39995706	-0.95768114	0.41025906
C	-0.03222300	0.37616106	0.44357506
C	-0.91482113	1.40200020	0.16319002
C	-2.23720832	1.07273415	-0.16244702
C	-2.66841838	-0.27820504	-0.19936902
C	-1.73914826	-1.26122718	0.08742702
H	-0.59799108	2.43856536	0.19860302
H	-2.02805530	-2.30632234	0.05980700
O	-3.17617346	1.99412028	-0.46494506
C	-2.80190640	3.36698348	-0.46136506
H	-2.47351236	3.67983852	0.53306708
H	-2.01068429	3.55806451	-1.19079016
H	-3.69659652	3.91977556	-0.74012010
C	0.54661708	-2.07739030	0.67204210
O	0.17702202	-3.23592946	0.65828010
C	2.01321929	-1.75870625	0.98502214
H	2.59940438	-2.58134936	0.57241108
H	2.10552130	-1.82553626	2.07305230
C	2.09870730	0.70894110	1.15867918
C	3.04573644	-0.38734806	-0.93928613
C	2.50213736	2.09198430	0.68632510
C	3.01689544	1.04015116	-1.50983822
C	3.46944650	2.07752230	-0.49199108
H	2.94416242	2.62036138	1.53753122
H	1.59632723	2.65141638	0.42553006
H	3.63931552	1.06995616	-2.40979034
H	1.99010628	1.27477518	-1.81871826
H	3.51787750	3.06969044	-0.94758914
H	4.47750064	1.83936226	-0.13859502
C	2.24954432	-1.29090318	-1.89802027
H	2.30575634	-2.34578634	-1.61772324
H	1.19735217	-0.99593514	-1.94018228
H	2.66589238	-1.19585118	-2.90550742
C	4.49429464	-0.90933713	-0.88674313
H	4.53225366	-1.91842628	-0.46691506
H	4.91170771	-0.95000714	-1.89782127
H	5.13171074	-0.26669304	-0.27502204
C	1.67762724	0.69803610	2.61449238
H	1.05780714	1.57331122	2.82401740
H	1.11909016	-0.18756202	2.91153242
H	2.57257337	0.77482312	3.24303046
C	2.45830036	-0.42138806	0.46888306
C	-4.12130159	-0.58120408	-0.51686708
H	-4.42233164	0.09543902	-1.32214720
C	-4.35402263	-2.01535129	-0.98819414
H	-3.70862754	-2.27472132	-1.83140426
H	-4.17397960	-2.73513840	-0.18472002
H	-5.39303976	-2.13097030	-1.30617818
C	-5.00226672	-0.27757304	0.70362810
H	-4.87652870	0.75341411	1.03996816
H	-6.05716287	-0.43386106	0.46142606
H	-4.74016568	-0.94536614	1.53015822

B

$E = -967.511009$ a.u.

C	-0.51440697	-1.00884441	0.08826738
C	-0.10143357	0.32472096	0.17354275
C	-1.07904341	1.32232267	0.07621115
C	-2.41958927	1.00064712	-0.11671841
C	-2.84326406	-0.34564293	-0.22691587
C	-1.86977454	-1.31738310	-0.12041160
H	-0.78530557	2.36034340	0.14745183
H	-2.13868226	-2.36538793	-0.18615858
O	-3.39438900	1.93057652	-0.22248000
C	-3.03732726	3.30235033	-0.10489442
H	-3.96508165	3.86119625	-0.20875719
H	-2.59366101	3.50992502	0.87252071
H	-2.34352154	3.59741899	-0.89641759
C	0.44160011	-2.12560728	0.27781400
O	0.13958279	-3.28601510	0.07477907
C	1.80962731	-1.73528957	0.78733109
H	2.48736370	-2.57208749	0.62859064
H	1.70077290	-1.60805792	1.87828207
C	1.50732162	1.25487734	1.82109295
H	0.81602737	2.08604241	1.98587313
H	1.27411666	0.47147020	2.54617176
H	2.52050000	1.61048864	2.01392949
C	2.27011550	-0.46365829	0.14355566
C	1.36568875	0.73289633	0.36538318
C	3.72478150	-0.33599053	-0.26528868
C	1.71544114	1.87470939	-0.61611618
H	1.34669462	1.60668593	-1.61328196
H	1.19739204	2.78724442	-0.31162871
C	3.21594163	2.13213675	-0.70285149
H	3.40049117	2.95699912	-1.39670766
H	3.60783706	2.44829684	0.26929464
C	3.93040945	0.87846858	-1.18850865

H	5.00596260	1.05538896	-1.29367137
H	3.54749565	0.62561121	-2.18529771
C	4.18819515	-1.58400868	-1.03669060
H	5.17106764	-1.39004195	-1.47684720
H	4.28734084	-2.46004378	-0.39239739
H	3.49336071	-1.82540326	-1.84692079
C	4.61680212	-0.19932189	0.98825605
H	4.42241863	-1.01374576	1.69275922
H	5.67156930	-0.24832970	0.69829821
H	4.45221995	0.74675262	1.50756957
C	-4.31633248	-0.65681087	-0.40342080
H	-4.71962119	0.08034444	-1.10395774
C	-5.05333324	-0.48592282	0.93244909
H	-6.12658093	-0.64821078	0.79959211
H	-4.68576805	-1.21689739	1.65936886
H	-4.90608447	0.51435690	1.34431995
C	-4.57510754	-2.04987475	-0.97267537
H	-4.02751291	-2.21512113	-1.90419240
H	-4.28865290	-2.83169778	-0.26362440
H	-5.64158045	-2.16867785	-1.17902154

TS$_{A-C}$

$E = -967.456529$ a.u.

C	-0.52794923	-1.16878452	-0.07674271
C	0.03007580	0.08550616	-0.15690469
C	-0.71854239	1.24262041	-0.25450539
C	-2.11388708	1.10856661	-0.28507920
C	-2.73980029	-0.16702450	-0.22350539
C	-1.93096656	-1.28390946	-0.12308324
H	-0.24987490	2.21762371	-0.31814222
H	-2.36771558	-2.27625896	-0.07428441
O	-2.95136892	2.16139218	-0.38507286
C	-2.38846291	3.46669027	-0.45385899
H	-1.75319393	3.57283146	-1.33687213
H	-3.23114974	4.15093983	-0.52724921
H	-1.81207282	3.69171264	0.44725021
C	0.36059301	-2.33649941	0.07035502
O	-0.03937906	-3.48377835	0.09209451
C	1.85123041	-2.02807086	0.20940084
H	2.33659553	-2.52997251	-0.63080748
H	2.18663461	-2.55814482	1.10386230
C	2.24089594	-0.56237317	0.25838903
C	2.97864095	-0.01012923	-0.97092126
C	2.70901144	1.53277347	1.57851933
C	3.09494126	1.51907416	-0.88236325
C	3.63827838	1.99057733	0.46032177
H	3.17596324	1.67056476	2.55963690
H	1.80552392	2.16275905	1.58977850
H	3.72702462	1.87043399	-1.70443992
H	2.09730625	1.95027936	-1.03351864
H	3.72772647	3.07993064	0.47341206
H	4.64261571	1.58865400	0.62287164
C	2.27733146	-0.35235522	-2.29302211
H	2.10403413	-1.42505110	-2.41192625
H	1.31767753	0.16134654	-2.37987299
H	2.91017252	-0.02918434	-3.12541193
C	4.38275101	-0.64681775	-1.00647123
H	4.32198776	-1.72717866	-1.16448303
H	4.96075618	-0.22244128	-1.83328875
H	4.93102454	-0.47542385	-0.07775023
C	1.62357971	-0.43732679	2.70372534
H	2.37283699	-0.52876270	3.49822780
H	0.87061460	0.27286103	3.06701575
H	1.13724250	-1.40383479	2.57852254
C	2.25072759	0.10437655	1.45242757
C	-4.25537293	-0.24400385	-0.22863809
H	-4.61380874	0.49534849	-0.95069062
C	-4.79102723	-1.61275617	-0.64322391
H	-4.37917655	-1.93570921	-1.60291647
H	-4.55922133	-2.37716468	0.10375814
H	-5.87862630	-1.56614149	-0.73815674
C	-4.80718248	0.14204398	1.15090098
H	-4.46233512	1.13170866	1.45671726
H	-5.90067183	0.14901714	1.13678775
H	-4.48110157	-0.58472157	1.90147442

C

$E = -967.508594$ a.u.

C	0.53691765	1.36079043	-0.13732493
C	-0.23481652	0.20369883	-0.14747725
C	0.41040899	-1.03375134	-0.18793448
C	1.80526997	-1.06985264	-0.23720459
C	2.59696957	0.11158759	-0.23549423
C	1.93471687	1.32320095	-0.18085461
H	-0.14799321	-1.95642128	-0.17344054
H	2.48827039	2.25599644	-0.17317036
O	2.50102434	-2.22605225	-0.29057063
C	1.78003861	-3.45255468	-0.28983470
H	2.53078861	-4.23867161	-0.33640632
H	1.19299543	-3.56177288	0.62600693
H	1.12336953	-3.52230747	-1.16111730
C	-0.32785942	2.54459795	-0.03548646
O	0.02500444	3.69975440	0.09292185
C	-1.75839126	2.05806898	-0.14851277
H	-2.07884697	2.32808747	-1.15841984
H	-2.41785166	2.57366768	0.55062267
C	-1.73458627	0.51444209	0.02873902
C	-2.07452527	0.13229355	1.46497028
C	-2.69382599	-0.18385835	-1.00056049
C	-2.43215350	-1.29259022	1.76472634
C	-2.91809102	-1.67116902	-0.66306413
C	-3.41217464	-1.89747049	0.76106543
H	-2.83100953	-1.35547031	2.78224752
H	-1.50828376	-1.89898865	1.76896941
H	-3.64224440	-2.06853033	-1.38230649
H	-1.99420549	-2.23498289	-0.82149479
H	-3.52792224	-2.96774057	0.95223233
H	-4.39864286	-1.44209901	0.89243117
C	-2.12940942	-0.10118312	-2.42233390
H	-1.89390151	0.92617859	-2.71440944
H	-1.22138999	-0.70122568	-2.52650274
H	-2.86819472	-0.48495122	-3.13263970
C	-4.05977167	0.52699955	-0.97000855
H	-4.01683690	1.50255919	-1.45918106
H	-4.80060068	-0.07583424	-1.50362163
H	-4.42049228	0.67932595	0.05077155
C	-1.42820104	0.88208473	2.58831625
H	-0.42608725	0.47622261	2.80295802
H	-1.30903600	1.94987989	2.39296021
H	-2.01207028	0.77219713	3.50685166
C	4.10832501	-0.00805595	-0.24935092
H	4.36260422	-0.81462896	-0.94307188
C	4.80955689	1.26507724	-0.71782375
H	4.43551327	1.60055256	-1.68861193
H	4.68094161	2.08036451	-0.00030117
H	5.88187857	1.07773917	-0.81260109
C	4.61645647	-0.40901031	1.14290593
H	5.69974756	-0.55700330	1.12502254
H	4.39255705	0.38189889	1.86544753
H	4.14958900	-1.33373349	1.48722012

TS$_{C-D}$

$E = -967.487190$ a.u.

C	0.45297219	1.37654351	-0.11521644
C	-0.31918073	0.17343159	-0.03905926
C	0.37998849	-1.06271051	-0.24140750
C	1.75494791	-1.05520623	-0.30312538
C	2.53503765	0.15344663	-0.22517822
C	1.84989136	1.34985091	-0.16176590
H	-0.15475603	-1.99655041	-0.30827785
H	2.38586012	2.29306742	-0.18311440
O	2.49170440	-2.18257080	-0.46644332
C	1.80603891	-3.42255294	-0.54827055
H	2.57515740	-4.18443360	-0.65946043
H	1.23067904	-3.61442256	0.36245542
H	1.13998037	-3.44825727	-1.41560998
C	-0.41086134	2.53503188	-0.23061560
O	-0.08463508	3.71335864	-0.27441090
C	-1.84608929	2.02891879	-0.32861749
H	-2.18986081	2.23446381	-1.34650834
H	-2.50112870	2.57169586	0.35640737
C	-1.81087379	0.51624407	-0.06080244
C	-1.51103186	0.14924578	1.35193055
C	-2.83018676	-0.31898852	-0.86700249
C	-1.84921192	-1.23743610	1.85910670
C	-2.94416882	-1.76381743	-0.34210143
C	-3.07702001	-1.84079580	1.17422877
H	-2.02064403	-1.16436076	2.93612725
H	-0.99499089	-1.91409959	1.73802682
H	-3.81345181	-2.22330659	-0.82321345
H	-2.07647524	-2.34841162	-0.66234593
H	-3.19512462	-2.88091768	1.48921857
H	-3.97543547	-1.30630430	1.49829717
C	-2.42410677	-0.36627320	-2.34202968
H	-2.35624027	0.63322347	-2.77998303
H	-1.45670564	-0.86169904	-2.46578850

H	-3.16870967	-0.92933440	-2.91324932
C	-4.20571777	0.35875828	-0.73955571
H	-4.22499781	1.32375585	-1.25083785
H	-4.97319561	-0.27405472	-1.19428877
H	-4.47900241	0.52783509	0.30575008
C	-1.27874335	1.16776955	2.43075292
H	-2.23289883	1.37242369	2.93367571
H	-0.59478711	0.76752006	3.18463198
H	-0.88066968	2.11993276	2.08376690
C	4.04597344	0.05548700	-0.22652508
H	4.32408270	-0.69457180	-0.97299586
C	4.73816240	1.36804494	-0.58624719
H	4.38833646	1.76256729	-1.54375319
H	4.57208659	2.13091322	0.17987353
H	5.81614571	1.20477084	-0.65926689
C	4.54248650	-0.44249606	1.13954649
H	5.62718831	-0.58171167	1.12377455
H	4.30433274	0.29198481	1.91522072
H	4.07883216	-1.39299774	1.41003105

D

E = −967.488426 a.u.

C	0.44189837	1.37352956	0.00864464
C	-0.33924965	0.15713884	0.17588205
C	0.37876828	-1.08334165	-0.08797944
C	1.73809993	-1.06880494	-0.22887998
C	2.51792094	0.15080272	-0.19034039
C	1.82924508	1.34749971	-0.10830950
H	-0.15015892	-2.02292737	-0.10681174
H	2.35696610	2.29156091	-0.19543392
O	2.47999723	-2.18871731	-0.43546483
C	1.79840618	-3.43102648	-0.49949002
H	2.56371689	-4.18645302	-0.66708242
H	1.27601282	-3.64271546	0.43859941
H	1.08378048	-3.44598183	-1.32804368
C	-0.40956810	2.52580348	-0.21557228
O	-0.06831530	3.69823208	-0.29928125
C	-1.83733492	2.02387098	-0.39367236
H	-2.11245556	2.20866704	-1.43633355
H	-2.53048776	2.59043794	0.23366772
C	-1.83699256	0.52662521	-0.07973674
C	-1.47001695	0.17770114	1.32289062
C	-2.77899577	-0.34890491	-0.92159739
C	-1.89648463	-1.18367091	1.87803661
C	-2.93542212	-1.76572538	-0.33509445
C	-3.12542537	-1.76121600	1.17695558
H	-2.11111836	-1.05268979	2.94121331
H	-1.07212847	-1.90049842	1.82478845
H	-3.79180917	-2.23722881	-0.82732167
H	-2.06401422	-2.37631002	-0.59097148
H	-3.30706146	-2.77713565	1.53765966
H	-4.00764830	-1.16852440	1.43956458
C	-2.25439048	-0.46141479	-2.35612658
H	-2.16148110	0.51843763	-2.83264735
H	-1.27429325	-0.94704525	-2.37528807
H	-2.94318487	-1.06141309	-2.95910585
C	-4.16363365	0.32473911	-0.94526665
H	-4.13340998	1.28616773	-1.46256170
H	-4.87421622	-0.31570614	-1.47556024
H	-4.54737600	0.49793423	0.06377320
C	-1.37458773	1.22795208	2.41062513
H	-2.36498733	1.36419618	2.85830126
H	-0.69992445	0.88119306	3.19844824
H	-1.02398492	2.20336545	2.07752044
C	4.02491068	0.06162944	-0.26631718
H	4.27032548	-0.69095285	-1.02150952
C	4.69404295	1.37604197	-0.66071317
H	4.30071364	1.76561593	-1.60317380
H	4.55870330	2.14057327	0.10972678
H	5.76821130	1.21633964	-0.78126674
C	4.58439689	-0.43110359	1.07824935
H	5.66761349	-0.56552234	1.01157163
H	4.37913311	0.30463053	1.86189362
H	4.13758444	-1.38288678	1.37122512

$TS_{D\text{-}B}$

E = −967.480494 a.u.

C	-0.39955544	-1.31500012	0.36690230
C	0.29929032	-0.08534209	0.61441404
C	-0.43284654	1.12003321	0.37750753
C	-1.76628116	1.07820234	0.02458658
C	-2.48502280	-0.15105295	-0.11728540
C	-1.76327022	-1.31608664	0.04866256
H	0.03804527	2.07744490	0.53590393
H	-2.22915640	-2.27817553	-0.13782428
O	-2.50310057	2.19363550	-0.20137186
C	-1.84879367	3.45310363	-0.15102420
H	-2.60144082	4.19261629	-0.41742307
H	-1.47337968	3.66305913	0.85458825
H	-1.02375277	3.49583012	-0.86828116
C	0.42302396	-2.48883348	0.10173456
O	0.05084762	-3.65179066	0.08780979
C	1.84456403	-2.06334177	-0.27148823
H	1.99903700	-2.35288103	-1.31360478
H	2.55566222	-2.63496180	0.33460779
C	2.00217633	-0.58437484	-0.05782987
C	1.67202678	-0.09614140	1.30899391
C	2.57854373	0.27781957	-1.16472383
C	2.24908318	1.27305820	1.71774848
C	2.71390452	1.74500404	-0.72004446
C	3.23218185	1.86704706	0.70733551
H	2.76516385	1.13687090	2.67070517
H	1.44039003	1.97854889	1.92036001
H	3.38690430	2.25041364	-1.41951200
H	1.74374595	2.24495872	-0.80416473
H	3.41909218	2.91468212	0.95657606
H	4.19268395	1.34828798	0.79065459
C	1.69683660	0.21695752	-2.42224378
H	1.61906751	-0.79933782	-2.81714680
H	0.68922584	0.58407565	-2.20710762
H	2.13196869	0.84573901	-3.20533297
C	3.97390918	-0.27781722	-1.52238918
H	3.91150131	-1.31085942	-1.87259793
H	4.40870607	0.32342868	-2.32645020
H	4.65241939	-0.25101513	-0.66634729
C	1.79851422	-1.07647445	2.47368407
H	1.33535651	-0.63286280	3.35907996
H	1.32596136	-2.04312977	2.30267778
H	2.85698930	-1.24708619	2.69156234
C	-3.96308374	-0.11948641	-0.44581048
H	-4.11362344	0.66461321	-1.19409064
C	-4.48375840	-1.43506700	-1.02036759
H	-3.91216830	-1.74938176	-1.89753008
H	-4.44280337	-2.23865465	-0.27935929
H	-5.52807532	-1.31667441	-1.31954000
C	-4.77342276	0.26619187	0.80093453
H	-5.83513481	0.35659359	0.55398775
H	-4.66711139	-0.50469189	1.57060347
H	-4.43549887	1.21753571	1.21629240

1. Gaussian 16, Revision A.03, Frisch, M. J. *et al.* Gaussian, Inc., Wallingford CT, 2016.
